# Egg Yolk Protein Homologs Identified in Live-Bearing Sharks: Co-Opted in the Lecithotrophy-to-Matrotrophy Shift?

**DOI:** 10.1093/gbe/evad028

**Published:** 2023-02-20

**Authors:** Yuta Ohishi, Shogo Arimura, Koya Shimoyama, Kazuyuki Yamada, Shinya Yamauchi, Taku Horie, Susumu Hyodo, Shigehiro Kuraku

**Affiliations:** Laboratory for Phyloinformatics, RIKEN Center for Biosystems Dynamics Research (BDR), Kobe, Japan; Department of Biology, Graduate School of Science, Kobe University, Kobe, Japan; Laboratory of Physiology, Atmosphere and Ocean Research Institute, University of Tokyo, Kashiwa, Japan; Laboratory of Physiology, Atmosphere and Ocean Research Institute, University of Tokyo, Kashiwa, Japan; Marine Science Museum, Tokai University, Shimizu, Japan; Husbandry Department, Environmental Aquarium Aquamarine Fukushima, Iwaki, Japan; Department of Marine Biology, School of Marine Science and Technology, Tokai University, Shimizu, Shizuoka, Japan; Laboratory of Physiology, Atmosphere and Ocean Research Institute, University of Tokyo, Kashiwa, Japan; Laboratory for Phyloinformatics, RIKEN Center for Biosystems Dynamics Research (BDR), Kobe, Japan; Molecular Life History Laboratory, National Institute of Genetics, Mishima, Japan; Depertment of Genetics, Sokendai (Graduate University for Advanced Studies), Mishima, Japan

**Keywords:** vitellogenin, very low-density lipoprotein receptor (VLDLR), chondrichthyes, viviparity, yolk, frilled shark, spotless smooth-hound

## Abstract

Reproductive modes of vertebrates are classified into two major embryonic nutritional types: yolk deposits (i.e., lecithotrophy) and maternal investment (i.e., matrotrophy). Vitellogenin (VTG), a major egg yolk protein synthesized in the female liver, is one of the molecules relevant to the lecithotrophy-to-matrotrophy shift in bony vertebrates. In mammals, all VTG genes are lost following the lecithotrophy-to-matrotrophy shift, and it remains to be elucidated whether the lecithotrophy-to-matrotrophy shift in nonmammalians is also associated with VTG repertoire modification. In this study, we focused on chondrichthyans (cartilaginous fishes)—a vertebrate clade that underwent multiple lecithotrophy-to-matrotrophy shifts. For an exhaustive search of homologs, we performed tissue-by-tissue transcriptome sequencing for two viviparous chondrichthyans, the frilled shark *Chlamydoselachus anguineus* and the spotless smooth-hound *Mustelus griseus*, and inferred the molecular phylogeny of VTG and its receptor very low-density lipoprotein receptor (VLDLR), across diverse vertebrates. As a result, we identified either three or four VTG orthologs in chondrichthyans including viviparous species. We also showed that chondrichthyans had two additional VLDLR orthologs previously unrecognized in their unique lineage (designated as VLDLRc2 and VLDLRc3). Notably, VTG gene expression patterns differed in the species studied depending on their reproductive mode; VTGs are broadly expressed in multiple tissues, including the uterus, in the two viviparous sharks, and in addition to the liver. This finding suggests that the chondrichthyans VTGs do not only function as the yolk nutrient but also as the matrotrophic factor. Altogether, our study indicates that the lecithotrophy-to-matrotrophy shift in chondrichthyans was achieved through a distinct evolutionary process from mammals.

**Significance**

Vertebrate reproductive modes are classified into oviparity (egg-laying) and viviparity (live-bearing), and the transition from oviparity to viviparity occurred multiple times in vertebrate evolution. Some viviparous sharks and rays can supply maternal nutrition to their fetus, sometimes via the placenta, like mammals. In this study, we focused on viviparous shark homologs of egg yolk proteins that got lost after the switch to viviparity in placental mammals. Our analysis revealed the retention of egg yolk protein homologs in sharks and rays, equipped with a possibly new role, as well as secondary modification of the gene repertoires encoding their receptors. These findings suggest the different molecular-level configurations of viviparity between mammals and cartilaginous fishes.

## Introduction

**Fig. 1.**
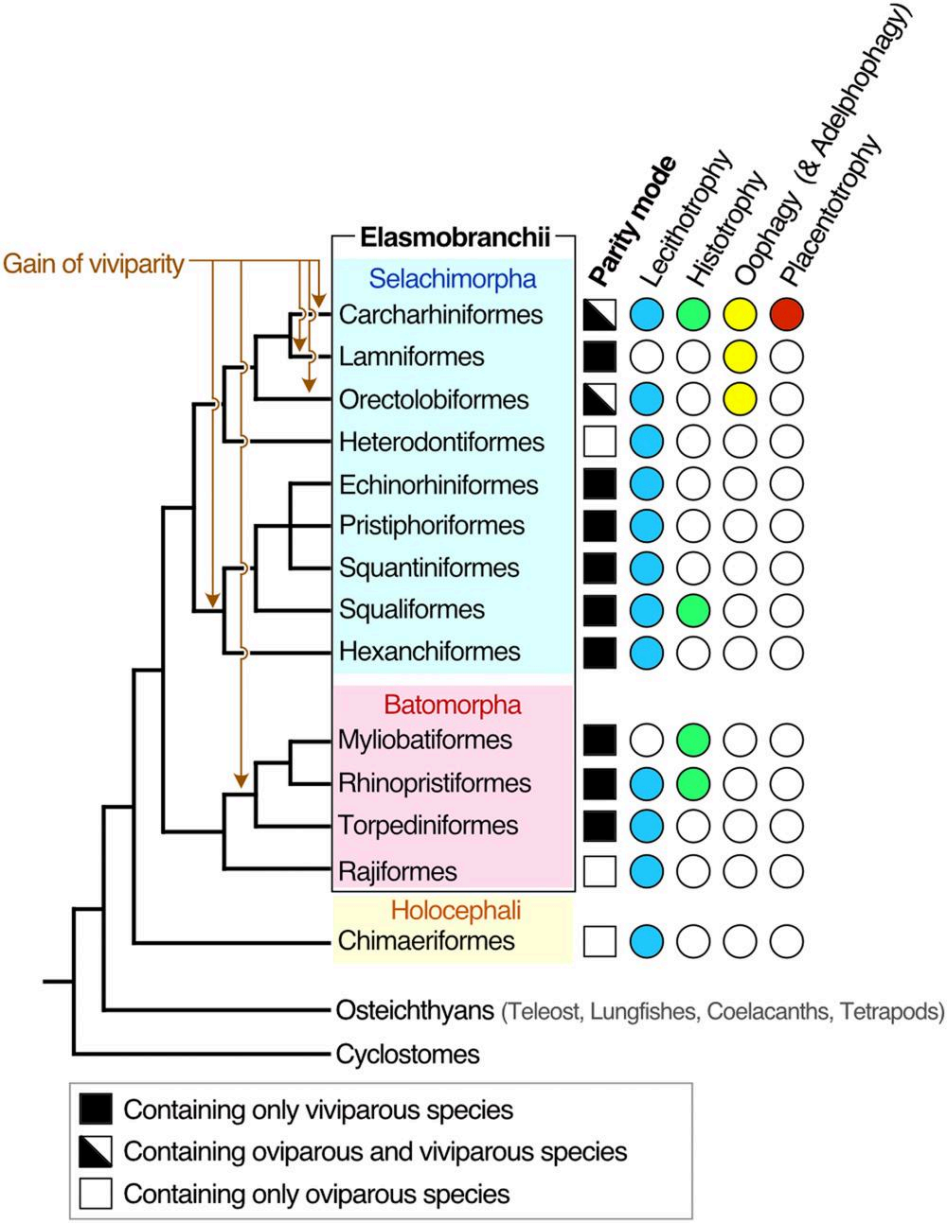
Phylogenetic tree of sharks and rays and the distribution of variable reproductive modes. Phylogenetic relationships are based on molecular studies (Aschliman et al. 2012; Naylor et al. 2012), and chondrichthyan reproductive modes are based on the literature (Buddle et al. 2019). Events in the acquisition of viviparous reproductive mode, shown in orange, are mapped on the basis of most parsimonious interpretation (Mull et al. 2022).

During vertebrate evolution, viviparity (i.e., live-bearing) occurred independently from oviparity (i.e., egg-laying) more than 150 times (Blackburn 2015). In many viviparous species, embryos develop by using nutrients deposited in their yolk sac, an embryonic nutrition mode called lecithotrophy (Wourms 1977; Blackburn 2015). In other viviparous species, embryos use various types of nutrient sources from pregnant females in addition to yolk nutrients to grow and these embryonic nutrition modes are collectively called matrotrophy (Wourms 1981). About 70% of cartilaginous fish species exhibit a viviparous reproductive mode (Wourms 1981; Blackburn 2015). In elasmobranchs (i.e., sharks, rays, and skates), a subgroup of cartilaginous fishes that excludes chimeras, viviparous reproductive patterns are classified into lecithotrophic viviparity and three types of matrotrophic viviparity: that is, histotrophy, oophagy (adelphophagy), and placentotrophy (Hamlett et al. 2005; Musick & Ellis 2005; Buddle et al. 2019; Penfold & Wyffels 2019) (fig. 1). In histotrophic species, the fetus develops by feeding on lipidic or mucous liquid nourishment secreted from the uterus, called uterine milk; this mode of reproduction is observed in Myliobatiformes and Lamniformes (Hamlett et al. 2005; Sato et al. 2016; Kina et al. 2021) (fig. 1). Oophagy is a mode of reproduction that supports development by feeding unfertilized eggs as nutrition to the fetus, and is observed in Lamniformes, Carcharhiniforms, and Orectolobiformes (Gilmore Jr et al. 2005; Sato et al. 2016) (fig. 1). Meanwhile, some carcharhiniform sharks have a placenta, a temporary fetal organ facilitating exchange nutrients, gas, and waste between mother and fetus (Hamlett 1989; Buddle et al. 2019) (fig. 1). The placenta of shark species shares physiological and functional characteristics with mammalian placenta, but few studies have compared them at the molecular level (Salmon et al. 2020; Foster et al. 2022).

Vitellogenin (VTG), a precursor of the major yolk protein characterized in various animals (Wahli et al. 1981; Wu et al. 2013), is one of the molecules related to animal reproductive modes. In oviparous vertebrates, this protein is synthesized in the female liver and transported via blood circulation to the ovaries, where it is internalized into growing oocytes by receptor-mediated endocytosis (Hara et al. 2016). After uptake into the oocyte, VTG is cleaved by proteases in five mature peptides and accumulates as the yolk platelet (Reading & Sullivan 2011). The genome of an oviparous vertebrate usually harbors multiple VTG genes that are derived from gene duplications (Silva 1989; Babin 2008; Biscotti et al. 2018; Yilmaz et al. 2018). Previous studies hypothesized that vertebrate VTG genes are phylogenetically classified into VTG1 (also called VTG of the S-region and teleost VTG-C) and VTG2 (also called VTG of the M-region and teleost VTG-A), and that VTG2 orthologs increased through independent gene duplications in bony fishes, amphibians, and amniotes (Biscotti et al. 2018; Carducci et al. 2019). Later in the amniote lineages, modern viviparous mammals (i.e., therians, including eutherians and marsupials) have lost all VTG genes from their genomes, concurrently with the acquisition of matrotrophic nutritional investment (Brawand et al. 2008). However, VTG repertoires of cartilaginous fishes that also exhibit matrotrophic viviparity of independent origins are still unclear because of the lack of genetic sequence information, particularly of viviparous species.

During the process of VTG uptake into oocytes, very low-density lipoprotein receptor (VLDLR) (also called VTGR or LR8) functions as a VTG receptor in various oviparous vertebrates (Bujo et al. 1994; Li et al. 2003; Hiramatsu et al. 2013). VLDLR belongs to the LDLR superfamily and is a single-spanning transmembrane protein containing eight repeating ligand-binding domains (Yang & Williams 2017). Bony vertebrates including viviparous mammals possess a single VLDLR ortholog (Morini et al. 2020), while VLDLR gene repertoires remain unknown in cartilaginous fishes. Gene repertoire information of VLDLR, as well as VTG, was largely limited by the scarcity of whole genome and transcriptome information for this taxon. Only recently, such large-scale sequence information for diverse cartilaginous fishes have been made available (Hara et al. 2018; Marra et al. 2019; Pearce et al. 2021; Rhie et al. 2021), which finally permits exhaustive searches of homologs and their phylogenetic profiling.

In this study, we focused on cartilaginous fishes and aimed to elucidate the possible modification of gene repertories and their functions upon the lecithotrophy-to-matrotrophy shift. For this purpose, we acquired novel tissue-by-tissue transcriptome data for two viviparous shark species, the frilled shark *Chlamydoselachus anguineus*, a lecithotrophic species, and the spotless smooth-hound *Mustelus griseus*, a matrotrophic species. We performed a molecular phylogenetic analysis covering major vertebrate taxa for comparative investigation of gene repertoires. This revealed a distinct set of gene repertoires in cartilaginous fishes, namely retained VTG orthologs and additional paralogs of VLDLR, the VTG receptor. To characterize their contribution to the lecithotrophy-to-matrotrophy shift, we compared gene expression profiles between viviparous and oviparous species, which unveiled extrahepatic expression of VTG orthologs only in viviparous sharks. This suggests an alteration of the VTG role in the lecithotrophy-to-matrotrophy shift in cartilaginous fishes.

## Results

### Transcriptome Sequencing of Two Viviparous Shark Species

We focused on two viviparous shark species sampled off the coast of Japan, the frilled shark and the spotless smooth-hound. The frilled shark belongs to the Hexanchiformes and exhibits lecithotrophic viviparity (Tanaka et al. 1990). The spotless smooth-hound belongs to the Carcharhiniforms and shows placental matrotrophic viviparity (Teshima 1975). To reconstruct as many transcript sequences as possible, sequencing libraries were prepared for multiple tissues for the frilled shark (i.e., telencephalon, metencephalon, medulla oblongata, eye, gill, heart, liver, and muscle) and the spotless smooth-hound (i.e., liver and uterus) (supplementary table S1, Supplementary Material online), and de novo assembly was performed using sequenced reads from all of these libraries of each species (Haas et al. 2013) (supplementary table S2, Supplementary Material online). The resulting contig sequences of the frilled shark contained orthologs of 3,291 genes (96.1%) out of the 3,354 BUSCO v5 vertebrate core genes, including those recognized as “fragmented.” On the other hand, orthologs of 3,052 vertebrate core genes were contained in the transcriptome of the spotless smooth-hound (91.0%) (supplementary table S2, Supplementary Material online). These transcriptome sequences were adopted for the downstream analysis.

### Identification of Chondrichthyan VTG Homologs

**Fig. 2.**
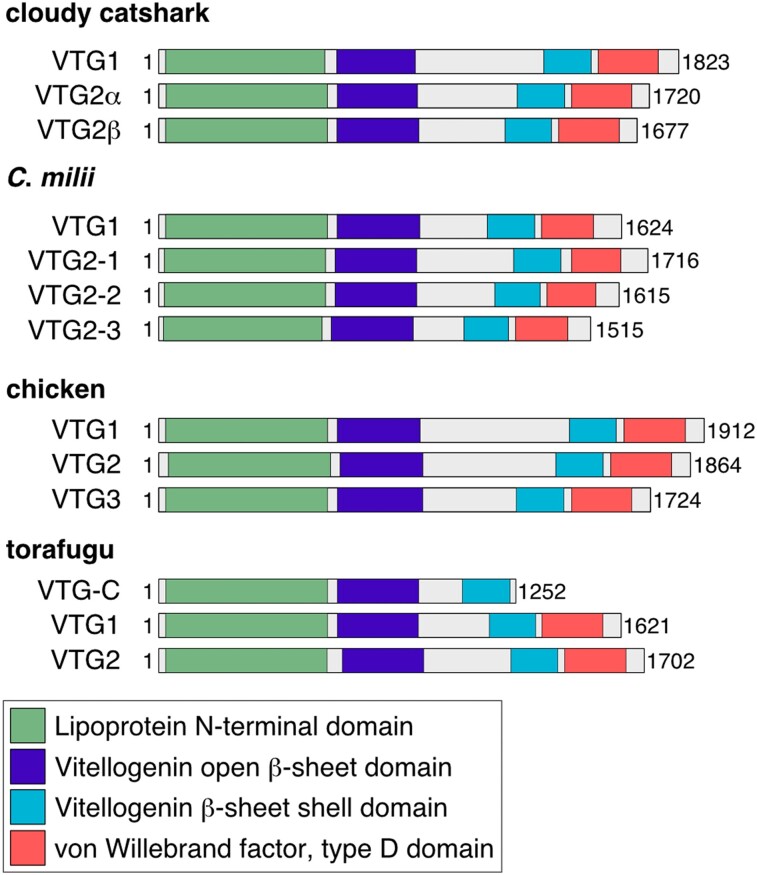
Structural properties of elasmobranch VTG ortholog products. Protein domain structures of the cloudy catshark and *C*. *milii* VTG genes in comparison with VTG homologs of the chicken and the torafugu (*Takifugu rubripes*), The four domains were identified by the webserver InterPro Search (Blum et al. 2021).

We first searched VTG orthologs in the genome and transcriptome sequence of cartilaginous fishes. Previous studies about chondrichthyan VTG genes reported one ortholog in the cloudy catshark in Elasmobranchii (Yamane et al. 2013), and three in *Callorhinchus milii* (elephant fish, elephant shark, or ghost shark) in Holocephali (Biscotti et al. 2018); however, the whole picture of VTG repertoires in cartilaginous fishes has been unclear (fig. 1). In the transcriptome assembly of the cloudy catshark, for which transcriptome information in various tissues was already obtained (Hara et al. 2018), we identified three VTG genes that show high similarity to amino acid sequences of the chicken VTG1 (NP_001004408.2) or VTG2 (NP_001026447.2) (fig. 2). We designated these previously reported and newly identified cloudy catshark genes as VTG1, VTG2α, and VTG2β. Multiple sequence alignment showed that VTG2α matched the previously reported catshark VTG gene (AEM05867.1), which differed from the VTG1 and VTG2β sequences (supplementary fig. S1, Supplementary Material online). The deduced amino acid sequences of three elasmobranch VTG genes harbored four protein domains (lipoprotein N-terminal domain, VTG open β-sheet domain, VTG β-sheet shell domain, and von Willebrand factor, and type D domain) (fig. 2), unlike those of actinopterygian VTG-C, which lack several C-terminal domains (Hara et al. 2016). We also identified partial sequences of potential VTG orthologs of the frilled shark and the spotless smooth-hound transcriptome assemblies (supplementary Supplementary data 1, Supplementary Material online). The continuity of these VTG sequences across different exons was validated by reverse transcription polymerase chain reaction performed using their liver tissues and assures their full-length open reading frames.

Furthermore, our search in the *C. milii* genome sequence revealed three VTG2 genes that correspond to ENSCMIT00000018226.1, ENSCMIT000000018289.1, and ENSCMIT000000018193.1 at the genomic region in which a previous study reported only two VTG genes (Biscotti et al. 2018). Although each of these VTG sequences was registered as a single transcribed gene in Ensembl, these sequences were recognized as separate genes by referring to their protein domain structures (fig. 2).

### Molecular Phylogenetic Analyses of Chondrichthyan VTG Genes

**Fig. 3.**
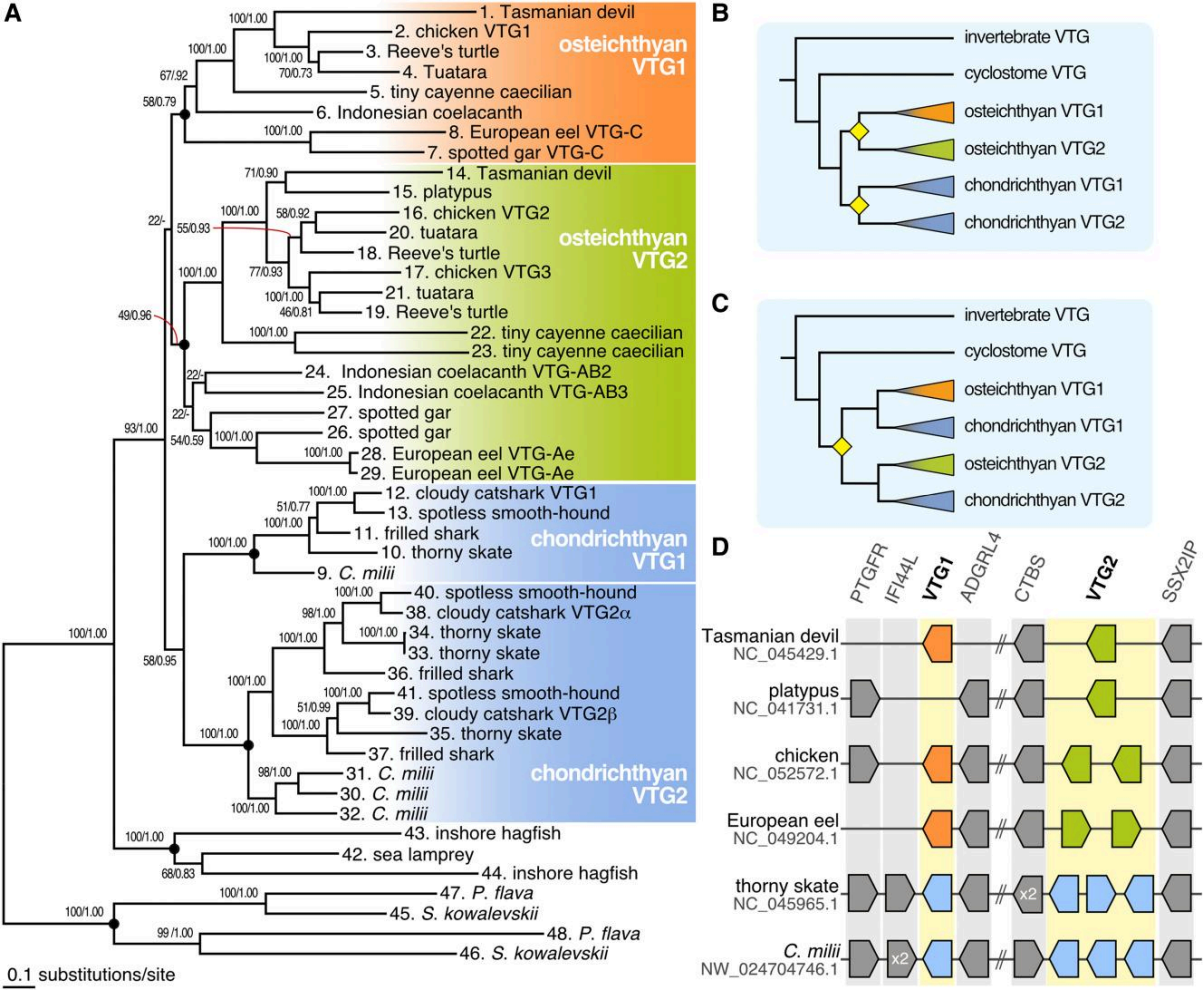
Molecular phylogeny and conserved synteny of elasmobranch VTG homologs. (*A*) Molecular phylogenetic tree of the VTG homologs. The tree was inferred with the ML method using 1,195 aligned amino acid sites. The JTT + I + G4 model was used in the analysis. The support values at nodes indicate bootstrap values and posterior probabilities based on the ML method and the Bayesian inference in order, respectively. The black circles plotted on each node represent the six groups fixed in the following statistical tree topology analysis (table 1). See Materials and Methods section for details. (*B*) The scenario in which gene duplications of VTG genes are postulated after the Chondrichthyes–Osteichthyes split is supported by our ML analysis. (*C*) The scenario in which gene duplications that gave rise to VTG1 and VTG2 preceded the Chondrichthyes–Osteichthyes split supported by the synteny analysis. The yellow rhombus in the phylogenetic tree of figures (C) and (D) indicates timings of VTG gene duplication. (*D*) Conserved synteny involving the VTG gene loci. Osteichthyan VTG1 orthologs are shown in orange, while osteichthyan VTG2 orthologs are shown in light green. Chondrichthyan VTG genes are shown in light blue.

To infer the orthology of elasmobranch VTG genes to osteichthyan VTG genes, we reconstructed molecular phylogeny with the maximum-likelihood (ML) method and the Bayesian approach. A previous study suggested that the first VTG gene duplication in the vertebrate lineage occurred in the common ancestor of gnathostomes based mainly on synteny conservations (fig. 3*C*) (Biscotti et al. 2018). However, the ML tree resulting from our inference displayed phylogenetic proximity of the chondrichthyan VTG1 genes to the chondrichthyan VTG2 genes (fig. 3*A*), indicating that VTG duplication occurred after the Osteichthyes–Chondrichthyes split (fig. 3*B*). Importantly, this phylogenetic proximity between the osteichthyan VTG1 and VTG2 was poorly supported in this ML tree (bootstrap value; 22).

To scrutinize the inconclusive result of the phylogenetic analysis, we performed an exhaustive phylogenetic analysis by evaluating all alternative tree topologies. In this analysis, internal relationships within six operational taxonomic units (OTUs) were constrained because the relationships among orthologs in them are consistent with the widely accepted species phylogeny. Out of 105 possible tree topologies, eight including the ML tree were revealed to be within 1σ of log-likelihood difference (table 1). However, the two above-mentioned scenarios (fig. 3*B*and*C*) could not be statistically rejected by approximately unbiased (AU) and Kishino–Hasegawa (KH) tests (Shimodaira & Hasegawa 1999; Shimodaira 2002). The result suggested that the molecular phylogenetic approach did not provide clear evidence about evolutionary history.

**Table 1:** Statistical Supports for Tree Topologies in ML-based Phylogenetic Analysis

Rank	Tree	Scenario	ΔlogL ± SE	ΔlogL/SE	*P*-value		
					AU	KH	SH
1	(((Ost-2,Ost-1),(Cho-1,Cho-2)),Cyc,OG);	figure 3 *B*	ML	–	0.771	0.655	1.000
2	((((Cho-1,Ost-1),Ost-2),Cho-2),Cyc,OG);	figure 3 *C*	3.20 ± 14.86	0.22	0.592	0.416	0.919
3	((((Cho-2,Cho-1),Ost-2),Ost-1),Cyc,OG);	figure 3 *B*	1.53 ± 4.58	0.33	0.495	0.345	0.939
4	((((Ost-2,Cho-2),Ost-1),Cho-1),Cyc,OG);	figure 3 *C*	6.75 ± 15.37	0.44	0.424	0.333	0.804
5	(((Ost-2,Cho-2),(Cho-1,Ost-1)),Cyc,OG);	figure 3 *C*	8.35 ± 15.66	0.53	0.259	0.296	0.767
6	((((Cho-2,Cho-1),Ost-1),Ost-2),Cyc,OG);	figure 3 *B*	2.48 ± 4.22	0.59	0.317	0.256	0.922
7	((((Ost-2,Cho-2),Cho-1),Ost-1),Cyc,OG);	figure 3 *C*	8.83 ± 15.04	0.59	0.228	0.275	0.749
8	((((Ost-2,Ost-1),Cho-1),Cho-2),Cyc,OG);	figure 3 *B*	7.83 ± 8.85	0.88	0.231	0.189	0.799
9	((((Cho-1,Ost-1),Cho-2),Ost-2),Cyc,OG);	figure 3 *C*	14.98 ± 14.51	1.03	0.008	0.151	0.542
10	((((Cho-1,Ost-1),Ost-2),Cyc),Cho-2,OG);	–	21.64 ± 17.27	1.25	0.030	0.107	0.313

Ost-1, osteichthyan VTG1; Ost-2, osteichthyan VTG2; Cho-1, chondrichthyan VTG1; Cho-2, chondrichthyan VTG-2; Cyc, cyclostome VTG; OG, outgroups; Δlog*L*, difference of log-likelihood deviated from the ML tree; SE, standard error of log-likelihood; AU, approximate unbiased test (Shimodaira 2002); KH, one-sided Kishino–Hasegawa test (Kishino & Hasegawa 1989); SH, Shimodaira–Hasegawa test (Shimodaira & Hasegawa 1999). For tree topologies supporting the scenarios hypothesized in figure 1*C*and*D*. All 105 tree topologies were sorted by Δlog*L*/SE.

To further investigate the orthology of vertebrate VTG genes, we examined gene synteny conservation across different vertebrate taxa. We first scanned the cloudy catshark genome assembly Storazame_v1.0 (GCA_003427355.1). While VTG1 is located on a 591 kb-long scaffold (scf_scyto00001130), full-length coding sequences of VTG2α and VTG2β were not included in this catshark genome assembly, which did not allow us to include this species in this analysis. Instead, we searched for VTG orthologs of the thorny skate, because its genome assembly exhibited an unprecedentedly high continuity among elasmobranchs (Rhie et al. 2021). As a result, four thorny skate VTG orthologs (VTG1; XP_032883733.1, VTG2; XP_032883739.1, XP_032883740.1, and XP_032883741.1) were identified on the approximately 70 Mbp-long sequence designated as chromosome 10 (NC_045965.1). We compared the composition of the genes flanking the thorny skate VTG genes with that in four non-elasmobranch vertebrate species (platypus, chicken, European eel, and *C*. *milii*) (fig. 3*D*). Our phylogenetic analyses on the flanking genes ADGRL4 (supplementary fig. S2*A*, Supplementary Material online) and SSX2IP (supplementary fig. S2*B*, Supplementary Material online) supported one-to-one orthology among these species, indicating that gene synteny in those genomic regions is conserved among the jawed vertebrates, which supported the previous hypothesis (Biscotti et al. 2018). Altogether, our analyses suggest that VTG gene duplication most likely occurred before the split between the osteichthyan and chondrichthyan lineages (fig. 3*B*). In a comprehensive search for vertebrate VTGs, we identified marsupial VTG orthologs, that is, Tasmanian devil VTG1 (XP_031825161.1) and VTG2 (XP_023357473.2), on the 464 Mbp-long scaffold designated as chromosome 4 (NC_045429), although marsupials were thought to have retained no VTG orthologs (Brawand et al. 2008) (fig. 3*A*). Remarkably, the Tasmanian devil VTG1 is not contained in the above-mentioned synteny blocks containing VTG orthologs in other vertebrates, suggesting that this genomic translocation may have facilitated the retention of the VTG1 gene (fig. 3*D*).

### Molecular Phylogenetic Analyses of Chondrichthyan VLDLR Genes

**Fig. 4.**
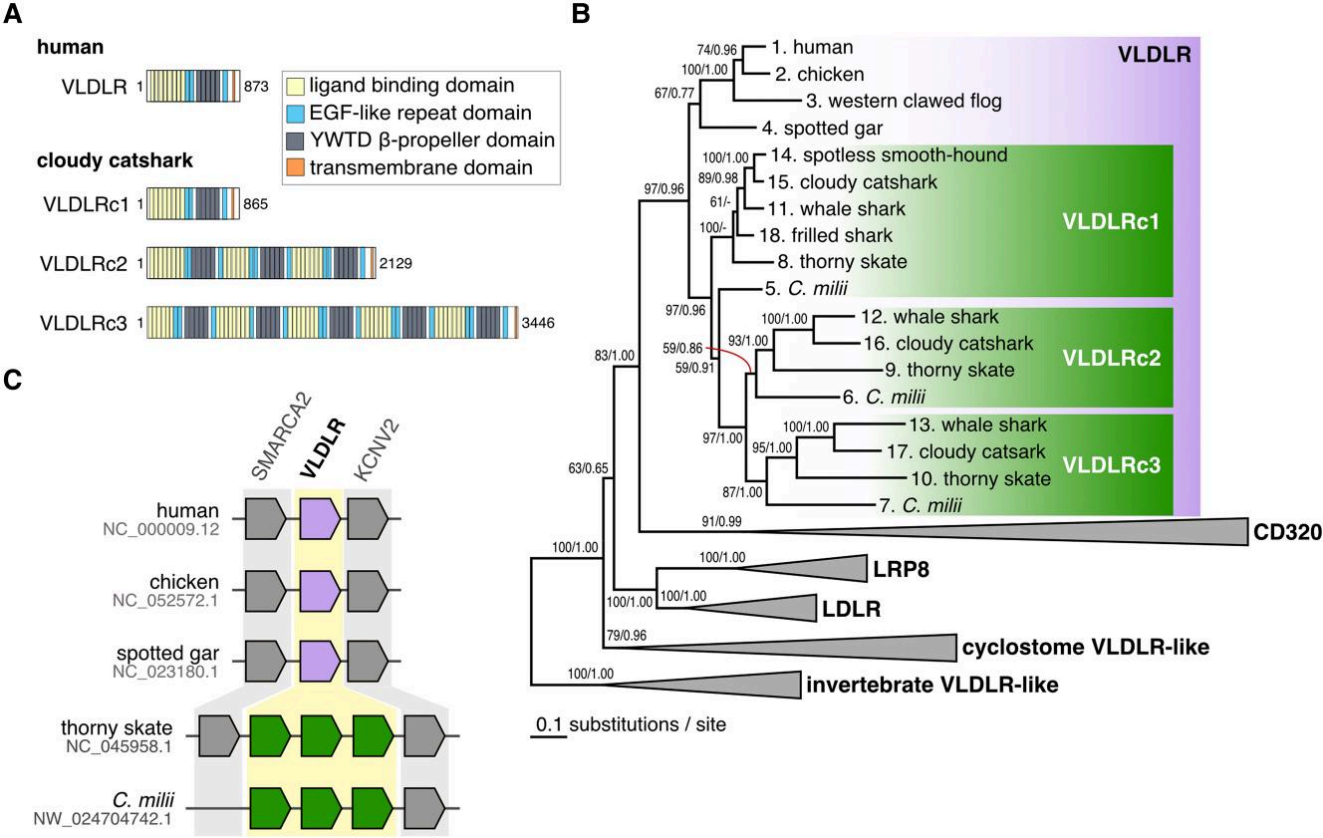
Structural and phylogenetic properties of chondrichthyan VLDLR homologs. (*A*) Protein domain structures of the cloudy catshark VLDLR orthologs in comparison with VLDLR ortholog of human. All domains were identified by the web server InterPro Search (Blum et al. 2021). (*B*) Molecular phylogenetic tree of the VLDLR genes and their relatives. The tree was inferred with the ML method using 615 aligned amino acid sites. The support values at nodes indicate bootstrap values and posterior probabilities based on the ML method and Bayesian inference in order, respectively. See the Materials and Methods section for details. (*C*) Conserved synteny involving the VLDLR gene loci between human, chicken, spotted gar, thorny skate, and *C*. *milii*. Osteichthyan VLDLR genes are shown in purple, while chondrichthyan VLDLR orthologs are shown in green.

In the genes predicted from the genome assembly of the cloudy catshark (GCA_003427355.1), we identified three VLDLR orthologs (Scyto0020726, Scyto0010396, and Scyto0010397) that showed high similarity to amino acid sequences of the human VLDLR (NP_003374.3) (fig. 4*A*). We also identified three VLDLR orthologs of the thorny skate (XP_032873693.1, XP_032874680.1, XP_032874678.1) from its genome assembly (GCF_010909765.2). Using these sequences and their relatives, we reconstructed phylogenetic trees with the ML method and the Bayesian approach. The ML tree displayed a phylogenetic proximity of the three chondrichthyans VLDLR genes to the osteichthyan VLDLR genes, indicating their orthologous relationships (fig. 4*B*). Furthermore, the tree topology and genomic locations of VLDLR genes on the thorny skate genome scaffold designated as chromosome 3 (NC_045958.1) (fig. 4*C*) suggest the occurrence of tandem gene duplication of the chondrichthyan VLDLR gene. These three genes are designated the VLDLR chondrichthyan type-1 (VLDLRc1), VLDLRc2, and VLDLRc3. The domain structure of VLDLRc1 resembled that of the human VLDLR, while VLDLRc2 and VLDLRc3 contained three and five repetitive units of the extracellular domain of VLDLRc1, respectively (fig. 4*A*).

### Chondrichthyan VTG and VLDLR Gene Repertoires

**Fig. 5.**
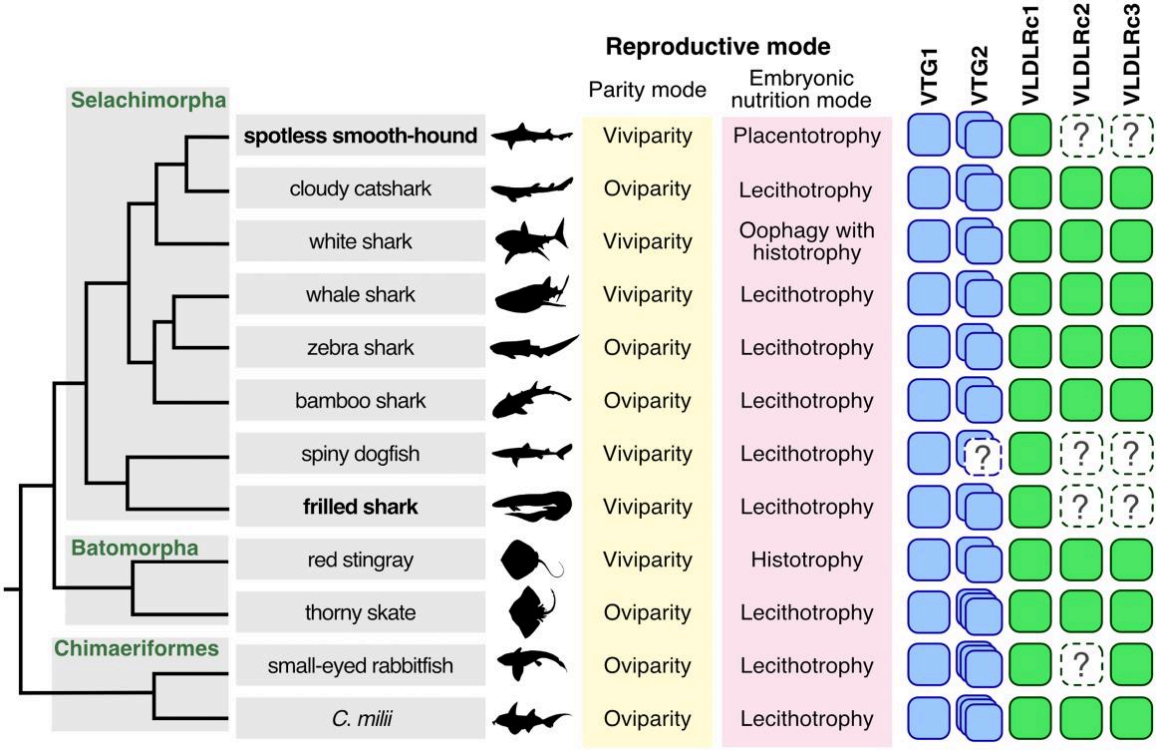
Cross-species comparison of VTG and VLDLR gene repertoires in Chondrichthyes. The colored box number on the right shows the number of orthologs identified in their genomes or transcriptome assemblies for individual species. Question marks indicate possible orthologs that could not be identified but are inferred only with transcriptome data and remain to be confirmed with whole genome sequencing. The reproductive mode of each species is shown to the right of the species name.

In the earlier analyses, we investigated molecular phylogenies for the whole Vertebrata (figs. 3 and 4). We next focused on the VTG and VLDLR gene repertoires of cartilaginous fishes. To examine a whole picture of chondrichthyan VTG and VLDLR gene repertoires, we searched for their homologs in 12 chondrichthyan species using their publicly available genomic or transcriptomic sequences (supplementary table S6, Supplementary Material online). Our molecular phylogenetic analysis of the VTG family detected four subgroups of chondrichthyan VTG genes, designated chondrichthyan VTG1, elasmobranch VTG2α, elasmobranch VTG2β, and holocephalan VTG2 (supplementary fig. S3, Supplementary Material online). This analysis supported the scenario that gene duplication of VTG2 occurred after the split between Chondrichthyes and Holocephali. Our scan missed a VTG2α ortholog of the spiny dogfish among the 12 chondrichthyan species, possibly due to incomplete transcriptome sequencing, but except for this, all examined species possessed consistent VTG gene repertoires (fig. 5). Similarly, our homolog search and molecular phylogeny reconstruction for the VLDLR family supported consistent gene repertoires, with three subtypes of chondrichthyan VLDLR, except for several cases where the ortholog could possibly not be identified yet (supplementary fig. S4, Supplementary Material online).

### Cross-species Comparison of VTG and VLDLR Gene Expression

**Fig. 6.**
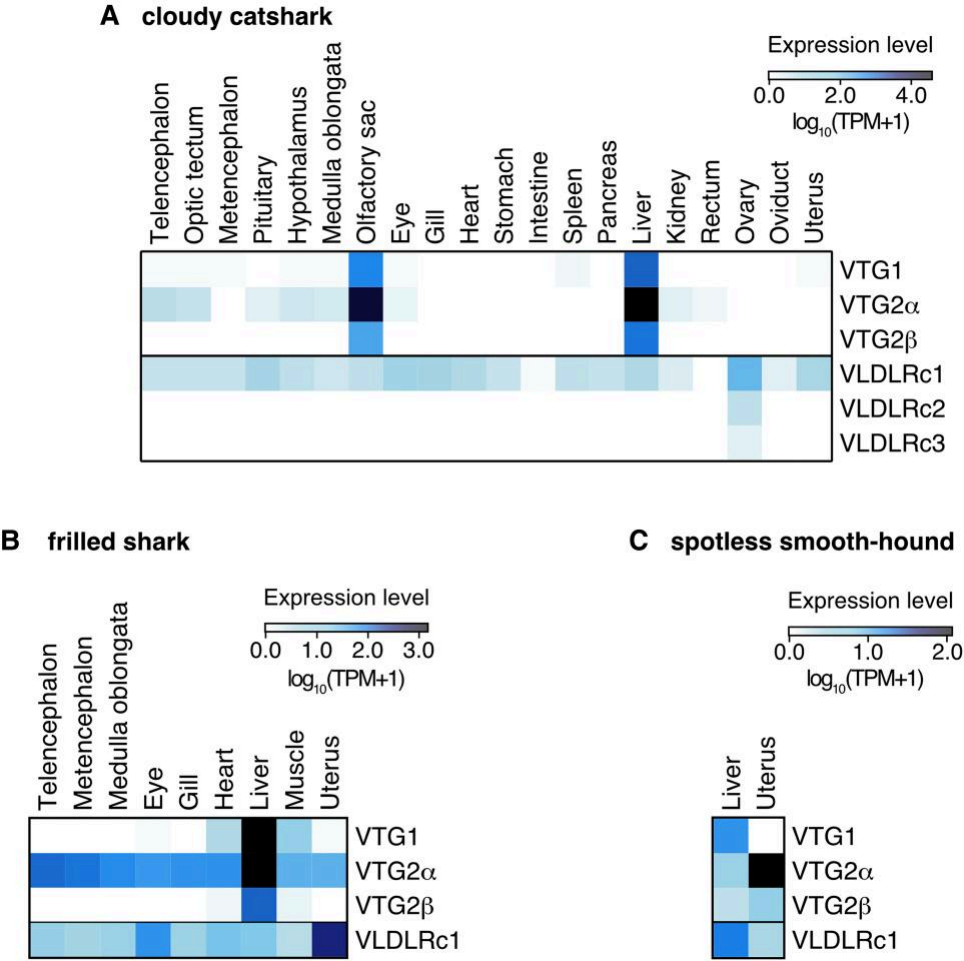
Cross-species comparison of expression profiles of elasmobranch VTG genes and VLDLR genes. (*A*) Heatmap for expression levels of VTG genes and VLDLR genes in tissues sampled from female adult catsharks. RNA-seq data for oviduct and uterus tissues were newly acquired in this study, while those of other tissues were obtained in our previous study (Hara et al. 2018). (*B*) Heatmap for expression levels of VTG genes and VLDLR genes in various tissues of adult female frilled shark. (*C*) Heatmap for expression levels of VTG genes and VLDLR genes from liver and uterus of adult female spotless smooth-hound. Their expression levels were shown as log_10_(TPM + 1). The genes whose sequences were not included in the transcriptome assembly (VLDLRc2 and -c3 of both frilled shark and spotless smooth-hound) are not included in (*B*) and (*C*). See the Materials and Methods section for technical details about RNA-seq data processing.

To examine the relationships between chondrichthyan VTG expression profile and their reproductive modes, we compared the tissue-by-tissue expression levels of the oviparous sharks with those of the viviparous sharks. We selected the frilled shark and the spotless smooth-hound as representatives of viviparous reproductive mode. Meanwhile, as a representative species of oviparous cartilaginous fishes, we selected the cloudy catshark, for which we previously released RNA-seq data (Hara et al. 2018). Expression of VTG orthologs was observed in the liver and olfactory sac of the adult female cloudy catshark (fig. 6*A*), whereas in adult male catsharks, these VTG orthologs were lowly expressed as reported in other vertebrates (supplementary fig. S5, Supplementary Material online). However, expression of VTG orthologs in the two viviparous sharks was observed in the liver and various other tissues, including the uterus (fig. 6*B*and*C*). Notably, the VTG2α ortholog of the spotless smooth-hound was more highly expressed in the uterus than in the liver. Taken together, these results in viviparous sharks suggest some physiological roles of their VTG gene products distinct from vitellogenesis.

We also quantified the expression levels of the shark VLDLR genes. The cloudy catshark VLDLRc1 was widely expressed in various tissues including the ovary, while the VLDLRc2 and VLDLRc3 orthologs were only expressed in the ovary (fig. 6*A*). In the frilled shark and the spotless smooth-hound, VLDLRc1 was expressed in a variety of tissues. In particular, the frilled shark VLDLRc1 was highly expressed in the uterus. On the other hand, VLDLRc2 and VLDLRc3 sequences were not identified in the transcriptome contigs; thus, their expression levels could not be quantified.

## Discussion

Previous studies tackled the origin of VTG genes in vertebrates (Finn & Kristoffersen 2007; Biscotti et al. 2018; Carducci et al. 2021). However, due to a lack of genetic information, elasmobranch VTGs were not included in those analyses. Our molecular phylogenetic analysis did not provide significant support for the hypothesis in a previous study (Biscotti et al. 2018) that the vertebrate VTG gene first duplicated before the Chondrichthyes–Osteichthyes split (fig. 3*A*and*B*). In general, molecular phylogenetic tree inferences are susceptible to systematic errors (Kapli et al. 2020) and can yield only ambiguous results. Such ambiguity in molecular phylogenetic analysis can be compensated by synteny investigation in judging orthology, as demonstrated for the orthology of CTCFL genes between mammals, nonmammalian tetrapods, and cartilaginous fishes (Kadota et al. 2020). In this study, we employed synteny analysis (fig. 3*D*) to scrutinize possible VTG orthology between chondrichthyans and osteichthyans that was not supported in our molecular phylogenic analysis. For this purpose, we inferred the molecular phylogeny of two neighboring genes SSX2IP and ADRL4 that are expected to share a common evolutionary history with VTG genes (supplementary fig. S2, Supplementary Material online) and concluded that the duplication between VTG1 and VTG2 genes occurred before the Chondrichthyes–Osteichthyes split (fig. 3*C*) as the gene duplication in those two neighboring gene families (supplementary fig. S2, Supplementary Material online). The inconsistency in the evolutionary scenarios between molecular phylogeny and synteny data may be reconciled by the phenomenon referred to as gene conversion. Gene conversion is a unidirectional transfer of genetic material from an intact homologous sequence to regions containing double-strand breaks. (Chen et al. 2007; Daugherty & Zanders 2019). Nevertheless, we could not find sufficient evidence for the VTG1–VTG2 gene conversion, probably because of the decay of nucleotide sequence-level traces that typically serve as evidence (e.g., the human globin genes, Slightom & Blechl 1980). Phylogenetic analysis of VTG2s in teleost fishes shows short branch lengths among paralogs of some species (supplementary fig. S4*A*, Supplementary Material online) and high conservation at the DNA sequence-level (supplementary fig. S4*B*, Supplementary Material online), suggesting gene conversion among the paralogous loci of VTG1 and VTG2. Furthermore, a previous study has also indicated a case of gene conversion between VTG2 genes in the amphibian lineage(s) (Carducci et al. 2021). These results may indicate a general susceptibility of vertebrate VTG genes to gene conversion.

Previously, the VTG gene repertoires of cartilaginous fishes were only partially uncovered (Yamane et al. 2013; Biscotti et al. 2018). Our analysis revealed that Holocephali and Elasmobranchii possess four and three VTG orthologs, respectively (fig. 5). In eutherians, the loss of VTGs occurred concurrently with the gain of matrotrophic nourishment, such as lactation and placentation (Brawand et al. 2008). In chondrichthyans, however, our results revealed no difference in VTG gene repertoire between matrotrophic species and lecithotrophic species (fig. 5). One possible explanation for the differences in the VTG gene fate between eutherians and chondrichthyans is their dependency on yolk nutrition for early development. Eutherian oocytes are smaller than those of other vertebrates because the former have no yolk content (Rothchild 2003; Frankenberg 2018). In contrast, most species of cartilaginous fishes have a yolk component in their oocytes, and even matrotrophic viviparous species grow using the yolk nourishment in their early development (Gilmore et al. 2005; Buddle et al. 2019; Furumitsu et al. 2019). In other words, the matrotrophic viviparity of cartilaginous fishes is achieved through a combination of yolk and maternal nutrition to support embryonic development.

Some viviparous bony vertebrates utilize VTG as a matrotrophic nutrient. For example, in the redtail split *Xenotoca eiseni*, the intraovarian embryos uptake VTG proteins through a pseudoplacenta, called the trophotaeniae, as one of the matrotrophic factors (Iida et al. 2019). Chondrichthyan VTG proteins may not only function as yolk nutrients but also as various matrotrophic nutrients. Our expression profiling of VTG genes in the two viviparous sharks revealed that VTG genes are expressed not only in the liver but also in the uterus (fig. 6*B*and*C*). In a previous study on the uterine milk of the red stingray *Hemitrygon akajei*, a histotrophic chondrichthyan species, the VTG2 protein was detected in the uterine milk (Kina et al. 2021). This report prompts us to postulate that VTGs expressed in the uterus may be co-opted as histotrophic nutrition in some cartilaginous fishes. Furthermore, oophagic chondrichthyan species also use VTG proteins as matrotrophic factors since unfertilized egg “yolk” is fed to the fetus (Gilmore, Jr et al. 2005). Taken together, such a co-option of VTG to matrotrophic nutrition may have also occurred in the lecithotrophy-to-matrotrophy shift in chondrichthyans.

Our transcriptome analysis revealed the VTG expression in various tissues other than the liver and uterus, such as the olfactory sac in the cloudy catshark (fig. 6*A*) and the brain in the frilled shark (fig. 6*B*). Extrahepatic VTG expression is also reported in zebrafish, although its physiological function is not yet clear (Wang et al. 2005). Several studies have demonstrated that VTG proteins have antibacterial functions via the VTG open β-sheet domain (DUF1943) (Shi et al. 2006; Liu et al. 2009; Sun et al. 2021) and activity to promote phagocytosis of microbes on interaction with the phagocytosis receptor plgR (Li et al. 2008; Liu et al. 2009; Sun et al. 2021). Such nonnutritive VTG functions may be the physiological significance of the extrahepatic expression in the cloudy catshark and the frilled shark.

Our phylogenetic analysis revealed that cartilaginous fishes have increased the number of VLDLR orthologs in their genomes from one to three by tandem gene duplications (fig. 4*B*). Furthermore, our transcriptome analysis showed that those VLDLR orthologs are highly expressed in the ovaries of the cloudy catshark (fig. 6*A*). These results imply that the VLDLRs likely regulate vitellogenesis. In addition, the VLDLRc1 expression in the uterus of two viviparous sharks suggests that their VTG is transported into the uterus for matrotrophic nutrition or other roles. Chondrichthyan VLDLR protein sequences harbor a repetitive extracellular domain that is not found in the homologs of other vertebrates, which may allow them to simultaneously bind multiple VTG ligands and support the maturation of yolk-rich oocytes (fig. 4*A*). Experimental characterization of ligand binding for the three shark VLDLRs belongs to future work.

## Conclusion

We conducted the first comprehensive investigation into the phylogeny of the gene encoding egg yolk protein and its receptor, encompassing all major vertebrate taxa. While therian VTG genes were lost after the shift of developmental nourishment resources, viviparous cartilaginous fishes have retained the VTG ortholog with novel expression in the uterus. We also discovered the VLDLR gene multiplicity in cartilaginous fishes, which may have enabled the maturation of their yolk-rich eggs. Altogether, our study showed a distinct evolutionary process of the lecithotrophy-to-matrotrophy shift in cartilaginous fishes from the counterpart in mammals.

## Materials and Methods

### Animals

Animal experiments were conducted in accordance with the guidelines approved by the Institutional Animal Care and Use Committee (IACUC), RIKEN Kobe Branch. The frilled shark tissues were sampled from frozen specimens (female 1: total length, 158 cm; female 2: total length, 168 cm) captured in Suruga Bay under relevant local fishery regulations and stored at −20 °C at the Marine Science Museum of Tokai University. The spotless smooth-hound tissues were sampled from a female (total length, 70 cm) captured in the Seto Inland Sea under relevant local fishery regulations. For both species, the individuals from which the uterus tissues were extracted were pregnant with eggs or fetuses. For the cloudy catshark, the oviduct and uterus were sampled from a captive mature female under the approval of the Animal Ethics Committee of Atmosphere and Ocean Research Institute of the University of Tokyo (P19-2).

### RNA-seq and *de novo* Transcriptome Assembly

Total RNA was extracted using a Direct-zol RNA extraction kit (Zymo Research). Quality control was performed with Bioanalyzer 2000 (Agilent Technologies) to ensure size distribution and quantity of the extracted RNA. The TruSeq Stranded mRNA Library Prep kit (Illumina) or Illumina Stranded mRNA Prep kit was used to create the mRNA libraries (see supplementary table S1, Supplementary Material online for details on which one was used). The libraries were sequenced with HiSeq X (Illumina) through outsourcing to Azenta Life Sciences. The obtained sequence reads were trimmed with TrimGalore version 0.6.7 (https://github.com/FelixKrueger/TrimGalore) with the options “–quality 30 –stringency 2 –length 25 –clip_R1 1 –clip_R2 1 –paired,” and de novo transcriptome assembly was performed with the Trinity program version 2.13.2 (Haas et al. 2013). The BUSCO program version 5.1.2 (Manni et al. 2021) was used to run a completeness assessment of transcriptome assemblies on the gVolante2 web server (Nishimura et al. 2017). Protein-coding sequences were predicted with the TransDecoder pipeline version 5.5.0 (https://github.com/TransDecoder/TransDecoder) and clustered by CD-HIT (Li & Godzik 2006).

### RT-PCR for cDNA Sequencing

Total RNA was reverse transcribed into cDNA using the SMARTer RACE 5′/3′ kit (Takara Bio). This cDNA was used as a template for PCR with sequence-specific primers designed based on the putative VTG contigs from de novo transcriptome assemblies and the putative VLDLR gene sequences from gene predictions of the cloudy catshark genome assembly (GCA_003427355.1). The primers are listed in supplementary table S5, Supplementary Material online.

### Molecular Phylogenetic Analysis

Protein sequences used for phylogenetic analysis were collected from the NCBI and the Ensembl databases using aLeaves (Kuraku et al. 2013), with the exception of those manually curated (supplementary Supplementary data 1, 2, Supplementary Material online). The accession IDs of the sequences used for the phylogenetic analysis are included in supplementary tables S3 and S4, Supplementary Material online. The predicted amino acid sequences were aligned with MAFFT version 7.487 (Katoh & Standley 2013) using the L-INS-i method. The aligned sequences were trimmed with trimAl version 1.4.rev22 (Capella-Gutierrez et al. 2009) using the “-automated1” option. This was followed by a trimAl run in the tree inference for figure 3*A*, supplementary figures S3 and S6*A*, Supplementary Material online with the option “-nogaps.” The ML tree was inferred with RAxML-ng version 1.1.0 (Kozlov et al. 2019) using the best model selected by modeltest-ng version 0.1.7 (Darriba et al. 2020). To evaluate the confidence of the nodes, the rapid bootstrap resampling with 1,000 replicates was performed. The molecular phylogenetic tree employing the Bayesian framework was inferred with PhyloBayes version 4.1b (Lartillot et al. 2009).

Evaluation of tree topologies in table 1 was performed with IQ-Tree version 2.1.4-beta (Minh et al. 2020) using JTT + I + G4 model. For all possible tree topologies and statistical tests, the internal relationships of the sequences used in the phylogenetic analysis were constrained to the following six OTUs; osteichthyan VTG1, osteichthyan VTG2, chondrichthyan VTG1, chondrichthyan VTG2, Cyc, cyclostome VTG, and the outgroup.

### Synteny Analysis

Gene ortholog names around VTG and VLDLR encoding genes on the genomes were obtained from NCBI RefSeq gene models for human (GCF_000001405.39), Tasmanian devil (GCF_902635505.1), platypus (GCF_004115215.2), chicken (GCF_016699485.2), European eel (GCF_013347855.1), spotted gar (GCF_000242695.1), *C*. *milii* (GCF_018977255.1), and thorny skate (GCF_010909765.2). Evolutionary conservation of synteny was confirmed by performing phylogenetic analysis of surrounding genes using the method described above.

### Gene Expression Quantification

We used previously published (Hara et al. 2018) and newly sequenced RNA-seq reads data in our analysis. For quantification of gene expression levels, the trimmed RNA-seq reads were mapped by bowtie2 version 2.3.5.1 (Langmead & Salzberg 2012) against de novo transcriptome assemblies of the cloudy catshark, the frilled shark, and the spotless smooth-hound to which full coding sequences of VTG and VLDLR were added. The mapping results were processed with RSEM version 1.3.3 (Li & Dewey 2011) to compute transcripts per million mapped reads (TPM).

## Supplementary Material

evad028_Supplementary_Data

## Data Availability

Sequencing read data were deposited in the DNA Data Bank of Japan (DDBJ) under accession number DRA014745. All newly identified cloudy catshark VTG and VLDLR sequences were deposited in DDBJ under accession numbers LC726232–LC726236.
