# Supplementary material for: Egg Yolk Protein Homologs Identified in Live-Bearing Sharks: Co-Opted in the Lecithotrophy-to-Matrotrophy Shift?
Source: Genome Biol Evol. 2023 Feb 20;15(3):evad028. doi: 10.1093/gbe/evad028 (PMC10015161; doi:10.1093/gbe/evad028)
Supplement: evad028_Supplementary_Data [file evad028_supplementary_data.pdf]

Supplementary Information

Egg Yolk Protein Homologs Identified in Live-Bearing Sharks: Co-opted in the Lecithotrophy-to-Matrotrophy Shift?

Yuta Ohishi[1,2], Shogo Arimura[3], Koya Shimoyama[3], Kazuyuki Yamada[4], Shinya Yamauchi[5], Taku Horie[6], Susumu Hyodo[3], Shigehiro Kuraku[1,7,8*]

[1]RIKEN Center for Biosystems Dynamics Research (BDR), Kobe, Japan.
[2]Department of Biology, Graduate School of Science, Kobe University, Kobe, Japan.
[3]Laboratory of Physiology, Atmosphere and Ocean Research Institute, University of Tokyo, Kashiwa, Japan.
[4]Marine Science Museum, Tokai University, Shimizu, Japan
[5]Environmental Aquarium Aquamarine Fukushima, Iwaki, Japan
[6]Department of Marine Biology, School of Marine Science and Technology, Tokai University, Shimizu, Shizuoka, Japan
[7]Molecular Life History Laboratory, National Institute of Genetics, Mishima, Japan
[8]Depertment of Genetics, Sokendai (Graduate University for Advanced Studies), Japan

[*]Correspondence should be directed to: skuraku@nig.ac.jp

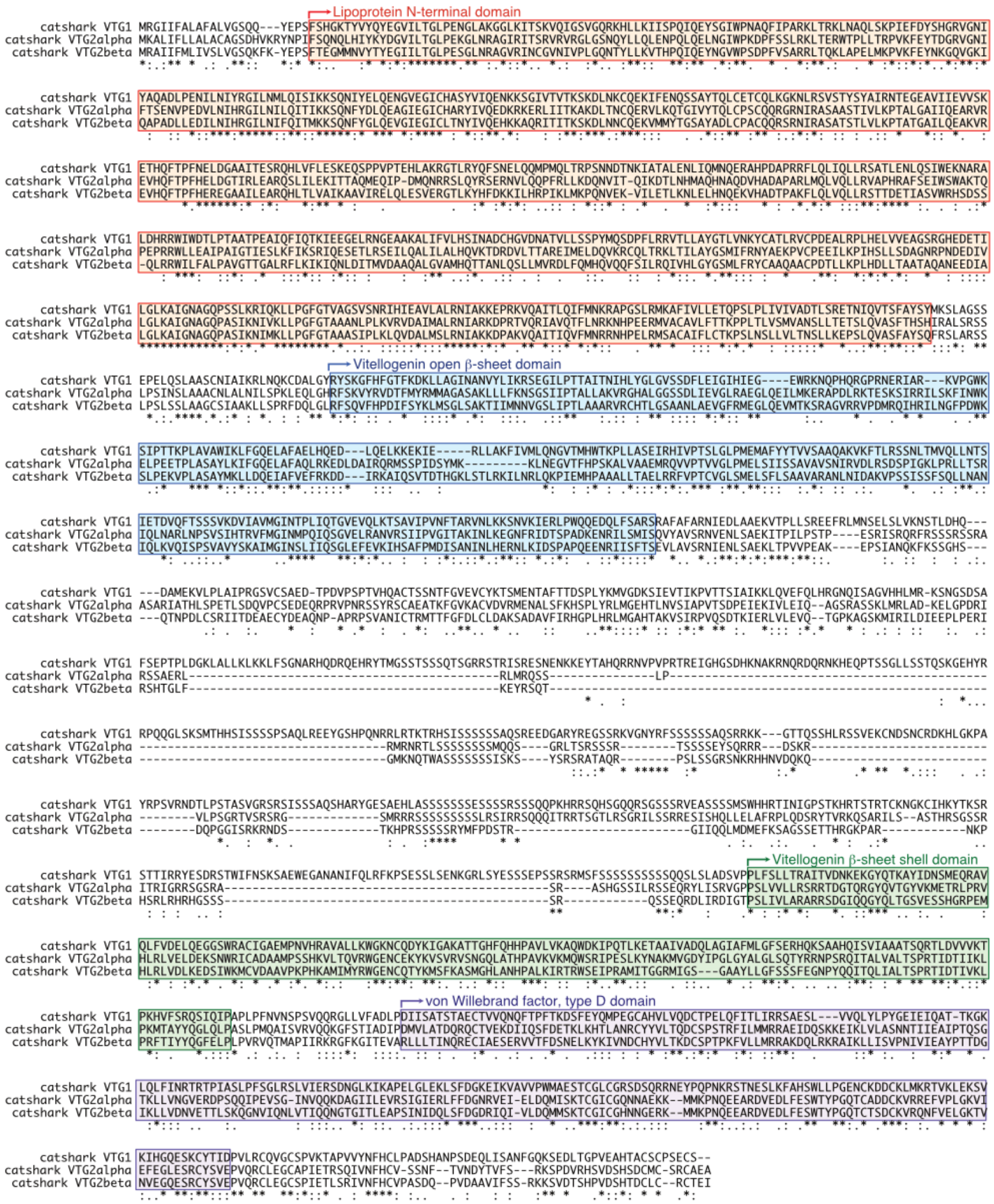

Supplementary fig. S1.—Alignment of deduced amino acid sequences of the cloudy catshark VTGs. Multiple alignments were performed with the iterative refinement method (L-INS-i) by the MAFFT program version 7.387 (Katoh & Standley 2013). Colored boxes indicate conserved domains inferred by the webserver InterPro Search (Blum et al. 2021). The asterisk, colon, and period shown below the alignment mean perfect match, strong property similarity (> 0.5), and weak property similarity (< 0.5) of the residues at that position, based on the PAM 250 matrix score, respectively.

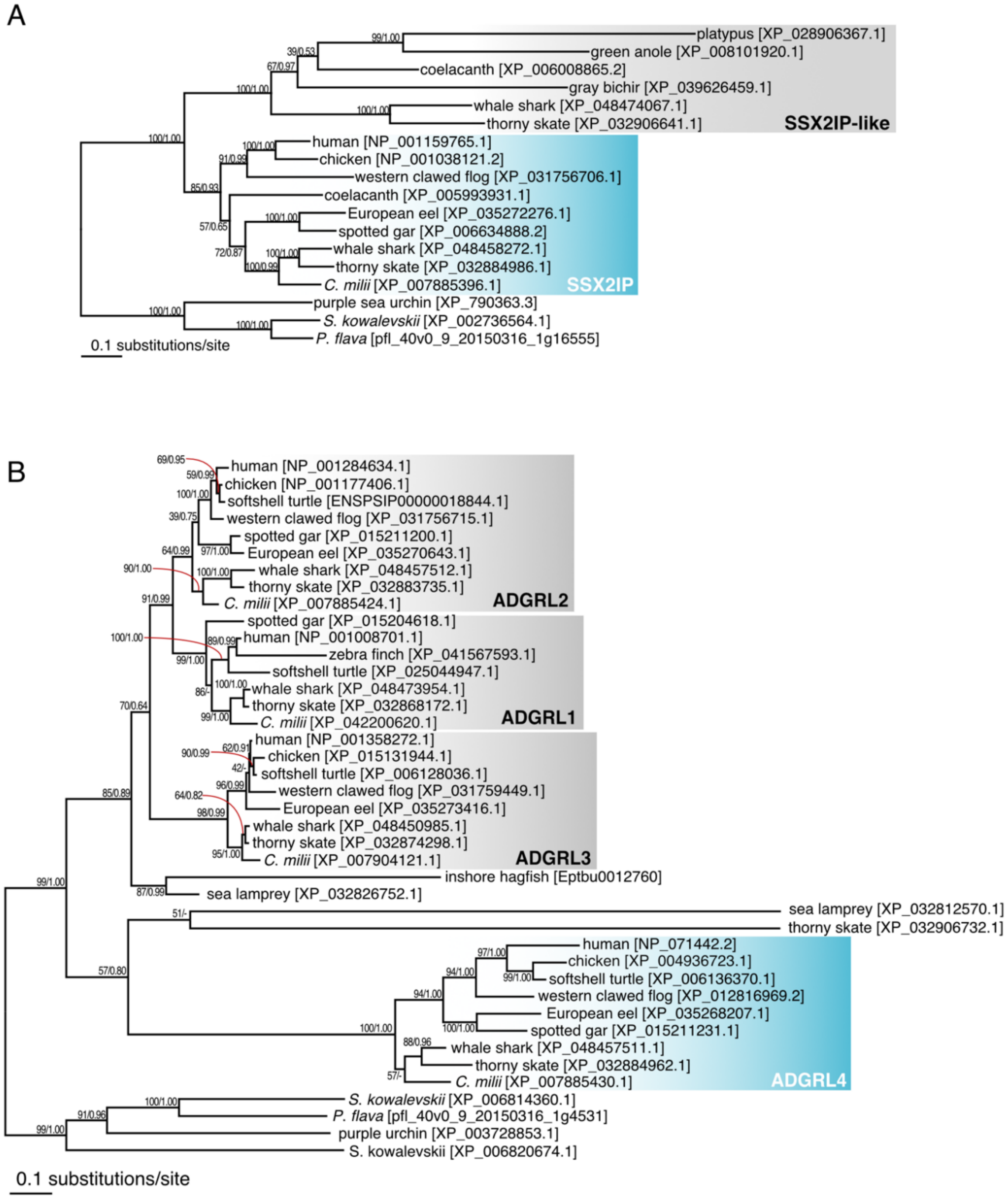

Supplementary fig. S2.—Molecular phylogenies of the SSX2IP and ADGRL4 genes. (A) Molecular phylogenetic tree of the SSX2IP genes and their relatives. The tree was inferred with the maximum-likelihood method using 417 aligned amino acid sites based on the JTT+I+G4. (B) Molecular phylogenetic tree of the ADGRL4 genes and their relatives. The tree was inferred with the maximum-likelihood method using 471 aligned amino acid sites based on the LG+I+G4. The support values at nodes of both trees indicate bootstrap values and posterior probabilities based on the maximum-likelihood method and Bayesian inference in order, respectively.

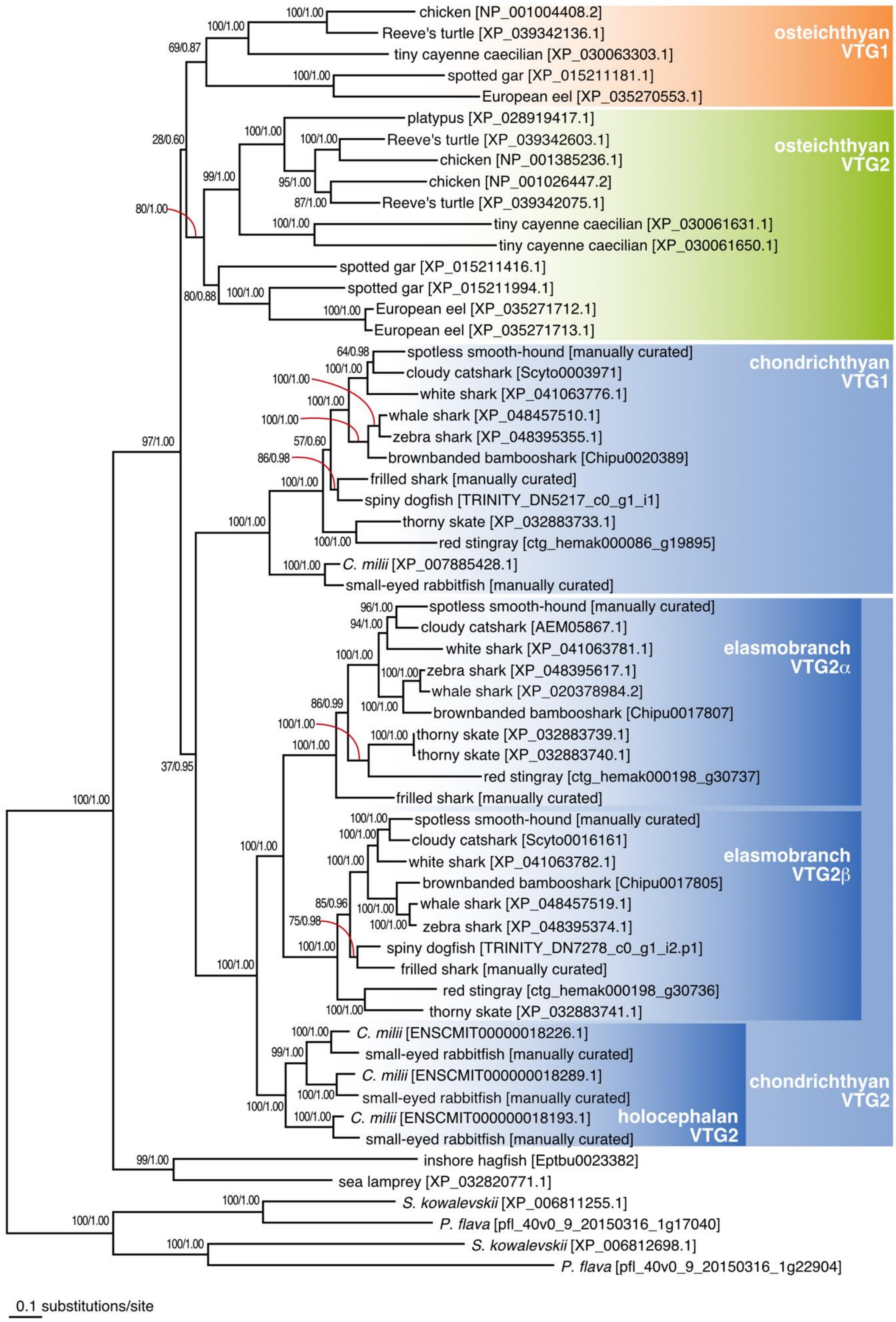
chicken [NP_001004408.2]
Reeve's turtle [XP_039342136.1]
tiny cayenne caecilian [XP_030063303.1]
spotted gar [XP_015211181.1]
European eel [XP_035270553.1]
osteichthyan VTG1
platypus [XP_028919417.1]
Reeve's turtle [XP_039342603.1]
chicken [NP_001385236.1]
chicken [NP_001026447.2]
Reeve's turtle [XP_039342075.1]
tiny cayenne caecilian [XP_030061631.1]
tiny cayenne caecilian [XP_030061650.1]
spotted gar [XP_015211416.1]
spotted gar [XP_015211994.1]
European eel [XP_035271712.1]
European eel [XP_035271713.1]
osteichthyan VTG2
spotless smooth-hound [manually curated]
cloudy catshark [Scyto0003971]
white shark [XP_041063776.1]
whale shark [XP_048457510.1]
zebra shark [XP_048395355.1]
brownbanded bambooshark [Chipu0020389]
frilled shark [manually curated]
spiny dogfish [TRINITY_DN5217_c0_g1_i1]
thorny skate [XP_032883733.1]
red stingray [ctg_hemak000086_g19895]
C. milii [XP_007885428.1]
small-eyed rabbitfish [manually curated]
chondrichthyan VTG1
spotless smooth-hound [manually curated]
cloudy catshark [AEM05867.1]
white shark [XP_041063781.1]
zebra shark [XP_048395617.1]
whale shark [XP_020378984.2]
brownbanded bambooshark [Chipu0017807]
thorny skate [XP_032883739.1]
thorny skate [XP_032883740.1]
red stingray [ctg_hemak000198_g30737]
frilled shark [manually curated]
elasmobranch VTG2α
spotless smooth-hound [manually curated]
cloudy catshark [Scyto0016161]
white shark [XP_041063782.1]
brownbanded bambooshark [Chipu0017805]
whale shark [XP_048457519.1]
zebra shark [XP_048395374.1]
spiny dogfish [TRINITY_DN7278_c0_g1_i2.p1]
frilled shark [manually curated]
red stingray [ctg_hemak000198_g30736]
thorny skate [XP_032883741.1]
elasmobranch VTG2β
C. milii [ENSCMIT00000018226.1]
small-eyed rabbitfish [manually curated]
C. milii [ENSCMIT000000018289.1]
small-eyed rabbitfish [manually curated]
C. milii [ENSCMIT000000018193.1]
small-eyed rabbitfish [manually curated]
holocephalan VTG2
chondrichthyan VTG2
inshore hagfish [Eptbu0023382]
sea lamprey [XP_032820771.1]
S. kowalevskii [XP_006811255.1]
P. flava [pfl_40v0_9_20150316_1g17040]
S. kowalevskii [XP_006812698.1]
P. flava [pfl_40v0_9_20150316_1g22904]
0.1 substitutions/site

Supplementary fig. S3.—Molecular phylogenetic tree of the chondrichthyan VTG homologs. The tree was inferred with the maximum-likelihood method using 928 aligned amino acid sites based on the JTT+F+I+G4. The support values at nodes of both trees indicate bootstrap values and posterior probabilities based on the maximum-likelihood method and Bayesian inference in order, respectively. VTG peptide sequences of the small-eyed rabbitfish are manually curated on the genomic scaffold sequence GCA_012026655.1, included in Supplementary data 1 and 2.

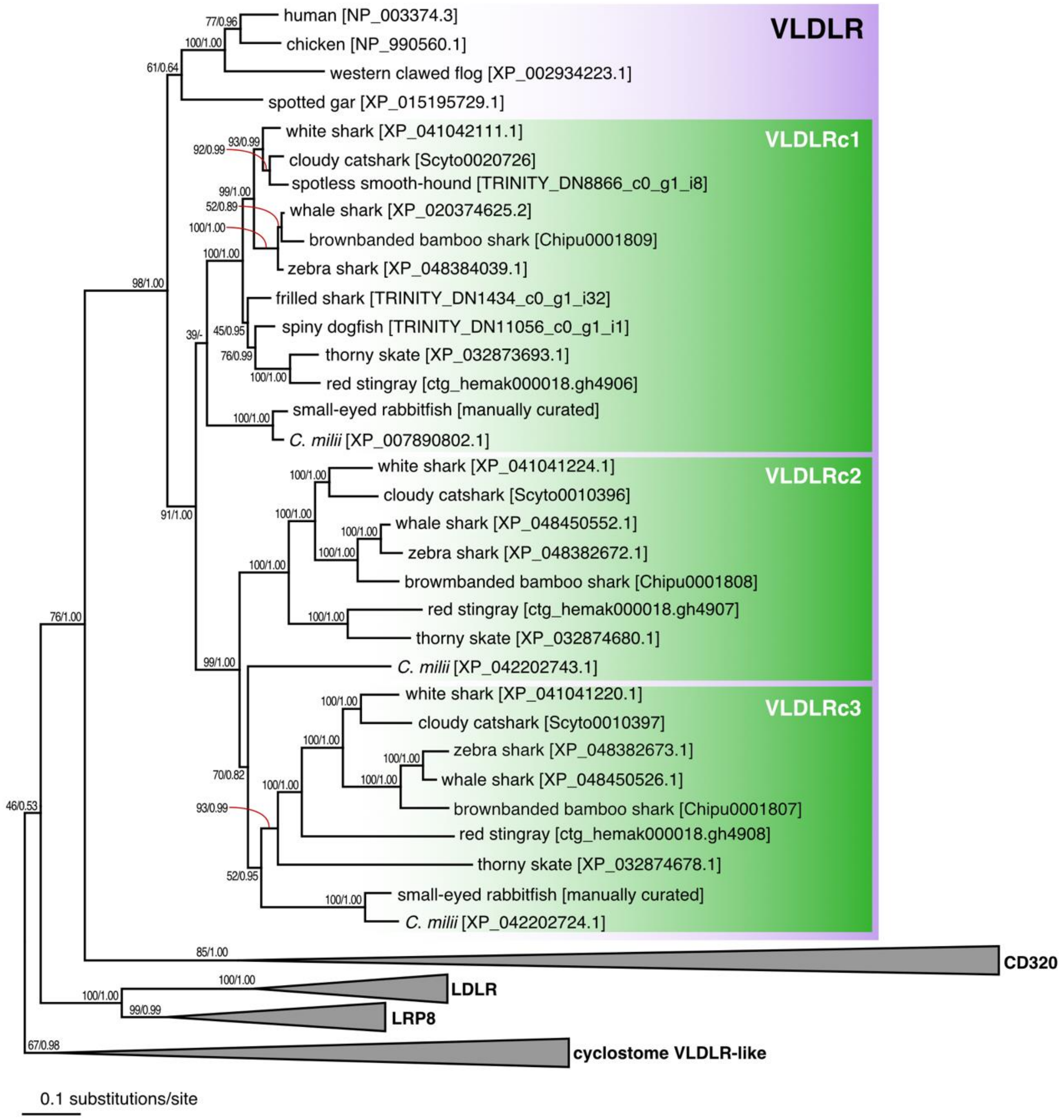

Supplementary fig. S4.—Molecular phylogenetic tree of the chondrichthyan VLDLR genes and its relatives. The tree was inferred with the maximum-likelihood method using 1,835 aligned amino acid sites based on the JTT+I+G4. The support values at nodes of both trees indicate bootstrap values and posterior probabilities based on the maximum-likelihood method and Bayesian inference in order, respectively. VTG peptide sequences of the small-eyed rabbitfish are manually curated using TBLASTN searching, as shown in Supplementary data 2.

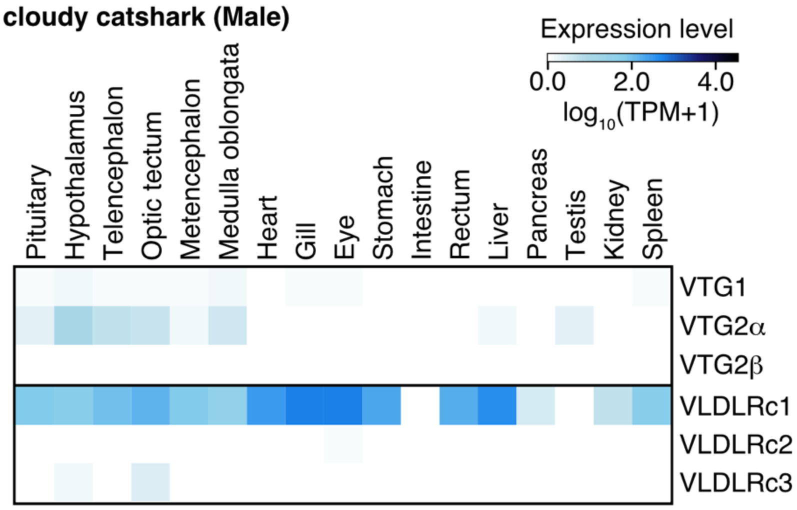

Supplementary fig. S5.—Expression profiles of VTG and VLDLR genes in male adult cloudy catshark. Heatmap for expression levels of VTG genes and VLDLR genes in tissues sampled from adult male catsharks previously released (Hara et al. 2018). Their expression levels were shown as log10(TPM+1). The expression level gradient is based on the scale of the female in fig. 6. See Materials and Methods section for technical details about RNA-seq data processing.

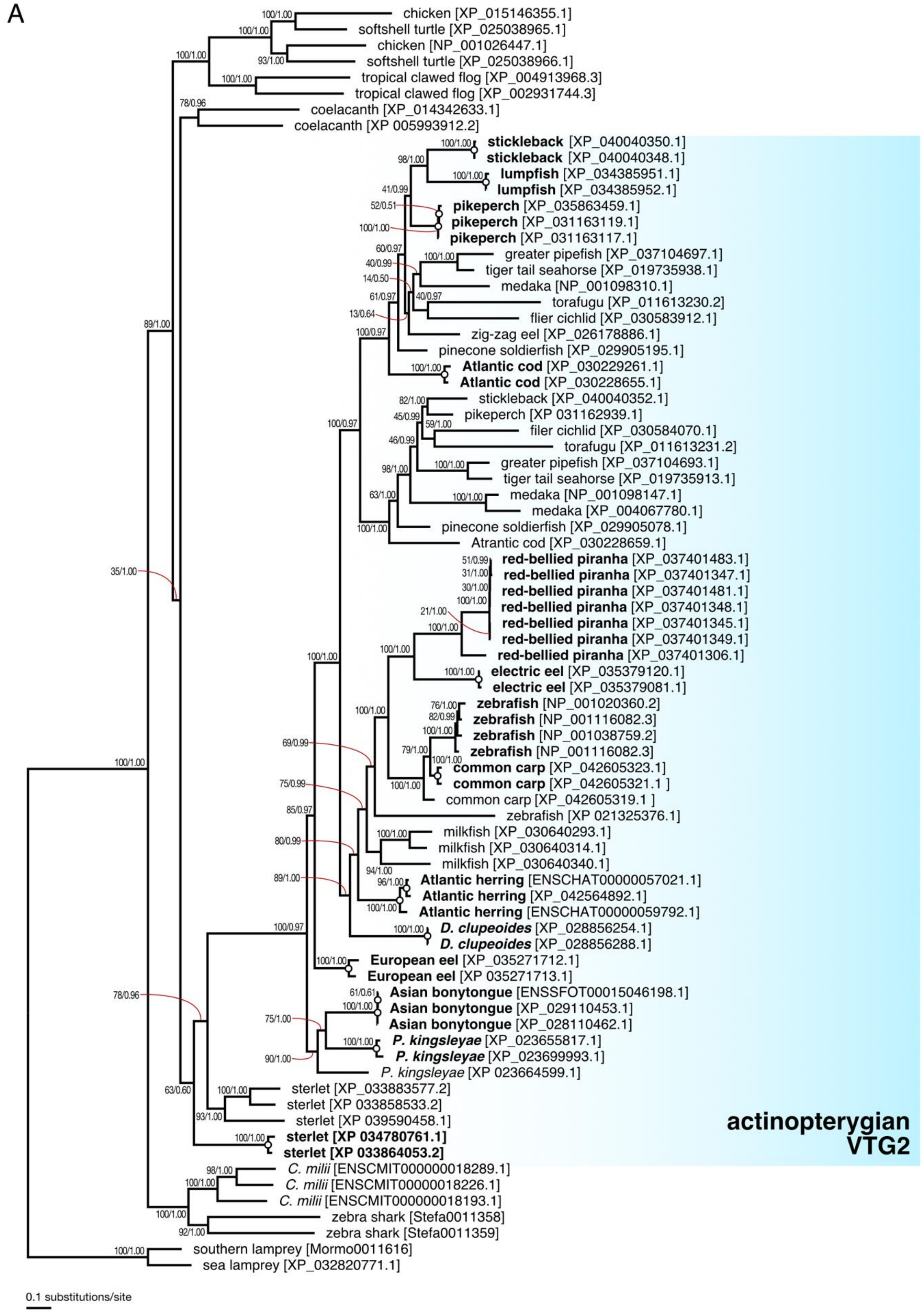

Supplementary fig. S6A.—Molecular phylogenetic tree of the actinopterygian VTG2 homologs. The tree was inferred with the maximum-likelihood method using 1,301 aligned amino acid sites based on the LG+I+G4. The support values at nodes of both trees indicate

bootstrap values and posterior probabilities based on the maximum-likelihood method and Bayesian inference in order, respectively. The open circles plotted on each nodes represent the traces of suspected gene conversion.

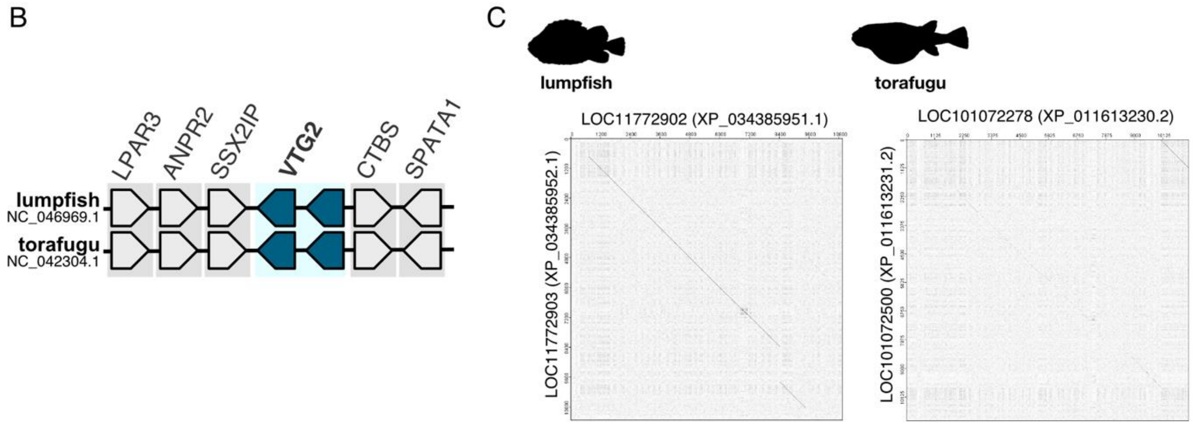

Supplementary fig. S6B and C.—Conserved gene synteny of actinopterygian VTG2 of the lumpfish and the torafugu. (B) Conserved synteny involving the VTG2 gene loci. The actinopterygian VTG2 orthologs are shown in dark blue. (C) Nucleotide sequence conservation of two VTG2 gene loci in the lumpfish and the torafugu, confirmed by the dot matrix method using JDotter (Brodie et al. 2004).

Supplementary Table 1. Properties of the RNA-seq libraries prepared in this study.

Organism	Tissues	Library prep kit	Purpose	Number of raw read-pairs	Number of qualified read pairs	Starting RNA amount (μg)	PCR cycles	Peak insert length (bp)	accession ID
cloudy catshark	Oviduct	Illumina Stranded mRNA Prep	E	37,029,467	35,785,290	0.245	10	291	DRR400763
	Uterus		E	47,805,413	45,860,412	0.245	10	292	DRR400764
frilled shark	Telencephalon	TruSeq Stranded mRNA Library Prep	A/E	28,085,764	27,548,139	1	7	284	DRR400765
			A	28,754,065	28,153,740	0.5	8	277	DRR400766
	Metencephalon		A/E	28,595,309	28,051,936	1	7	296	DRR400767
			A	29,397,372	28,949,636	1	7	293	DRR400768
	Medulla		A/E	32,645,289	32,121,080	1	7	290	DRR400769
	oblongata		A	30,761,392	30,280,028	1	8	289	DRR400770
	Eye		A/E	34,946,264	34,302,314	0.5	10	294	DRR400771
			A	31,446,647	30,806,149	0.5	10	289	DRR400772
	Gill		A	31,519,047	30,992,892	1	8	290	DRR400773
			A/E	31,156,721	30,647,321	1	7	294	DRR400774
	Heart		A/E	33,422,117	32,675,187	0.5	8	287	DRR400775
			A	27,837,341	27,347,509	0.1	11	279	DRR400776
	Liver		A/E	26,281,202	25,830,939	1	10	279	DRR400777
			A	31,168,000	30,587,297	0.3	12	278	DRR400778
	Muscle		A/E	30,892,214	30,271,145	1	7	291	DRR400779
			A	26,066,494	25,586,752	0.5	10	278	DRR400780
	Uterus		E	137,445,669	133,901,965	1	10	269	DRR400781

spotless	Liver	Illumina Stranded mRNA Prep	A/E	35,904,094	34,414,812	0.5	8	297	DRR400782
smooth-hound	Uterus	Illumina Stranded mRNA Prep	A/E	33,340,361	31,968,755	0.5	8	296	DRR400783

For the frilled shark, the uterus was sampled from Female 2, and the other tissues were sampled from Female 1 (see Materials and Methods). †Purpose of data production is listed with the following abbreviations: A, *de novo* transcriptome assembly, E, tissue-by-tissue gene expression quantification.

Supplementary Table 2. Transcriptome assembly results.

Organism	Total number of Contigs	Coding contigs	Number of conserved single copy orthologs			N50 contig length (bp)
			Only Complete	Including Fragmented	Missing	
frilled shark	884,087	96,931	3,223 (96.09 %)	3,291 (98.12 %)	63 (1.88%)	1,642
spotless smooth-hound	178,907	37,700	2,890 (86.17 %)	3052 (91.00%)	302(9.00%)	2,009

*Measured with BUSCO v3 using Vertebrata core orthologs (3,354 genes).

Supplementary Table 3. Identifiers of the sequences used for VTG phylogeny inference.

Group	No. in fig. 3A	Species	Gene name	Accession ID	Data source
osteichthyan VTG1/C	1	Tasmanian devil	vtg1	XP_031825161.1	NCBI
	2	chicken	vtg1	NP_001004408.2	NCBI
	3	Reeve's turtle	vtg1	XP_039342136.1	NCBI
	4	tuatara	vtg1	see Supplementary Data 2	This study
	5	tiny cayenne caecilian	vtg1	XP_030063303.1	NCBI
	6	Indonesian coelacanth	vtgABI	CCG55372.1	NCBI
	7	spotted gar	vtg3/C	XP_015211181.1	NCBI
	8	European eel	vtg3/C	XP_035270553.1	NCBI
chondrichthyan VTG1	9	*C. milii*	vtg3	XP_007885428.1	NCBI
	10	Thorny skate	vtg1	XP_032883733.1	NCBI
	11	frilled shark	vtg1	See Supplementary Data 1	This Study
	12	cloudy catshark	vtg1	See Supplementary Data 1	This Study
	13	spotless smooth-hound	vtg1	See Supplementary Data 1	This Study
osteichthyan VTG2	14	Tasmanian devil	vtg2	XP_023357473.2	NCBI
	15	platypus	vtg2	XP_028919417.1	NCBI
	16	chicken	vtg2	NP_001026447.2	NCBI
	17	chicken	vtg3	NP_001385236.1	NCBI
	18	Reeve's turtle	vtg2	XP_039342075.1	NCBI
	19	Reeve's turtle	vtg2	XP_039342603.1	NCBI
	20	tuatara	vtg2	see Supplementary Data 2	This study
	21	tuatara	vtg3	see Supplementary Data 2	This study
	22	tiny cayenne caecilian	vtgA2	XP_030061631.1	NCBI
	23	tiny cayenne caecilian	vtgA2	XP_030061650.1	NCBI
	24	Indonesian coelacanth	vtgABII	CCG55373.1	NCBI
	25	Indonesian coelacanth	vtgABIII	CCG55374.1	NCBI
	26	spotted gar	vtg	XP_015211994.1	NCBI
	27	spotted gar	vtg	XP_015211416.1	NCBI
	28	European eel	vtg2	XP_035271712.1	NCBI
	29	European eel	vtg	XP_035271713.1	NCBI
chondrichthyan VTG2	30	*C. milii*	vtg	ENSCMIT00000018226.1	Ensembl
	31	*C. milii*	vtg	ENSCMIT000000018289.1	Ensembl
	32	*C. milii*	vtg	ENSCMIT000000018193.1	Ensembl
	33	thorny skate	vtg2α-1	XP_032883739.1	NCBI
	34	thorny skate	vtg2α-2	XP_032883740.1	NCBI
	35	thorny skate	vtg2β	XP_032883741.1	NCBI

	36	frilled shark	vtg2α	see Supplementary Data 1	This Study
	37	frilled shark	vtg2β	see Supplementary Data 1	This Study
	38	cloudy catshark	vtg2α	AEM05867.1	NCBI
	39	cloudy catshark	vtg2β	see Supplementary Data 1	This Study
	40	spotless smooth-hound	vtg2α	see Supplementary Data 1	This Study
	41	spotless smooth-hound	vtg2β	see Supplementary Data 1	This Study
cyclostome VTG	42	sea lamprey	vtg	XP_032820771.1	NCBI
	43	inshore hagfish	vtg	Eptbu0023382	Yamaguchi et al. 2020
	44	inshore hagfish	vtg	ENSEBUG00000003292.1	Ensembl
invertebrate VTG	45	*S. kowalevskii*	vtg	XP_006811255.1	NCBI
	46	*S. kowalevskii*	vtg	XP_006812698.1	NCBI
	47	*P. flava*	vtg	pfl_40v0_9_20150316_1g17040	Simakov et al. 2015
	48	*P. flava*	vtg	pfl_40v0_9_20150316_1g22904	Simakov et al. 2015

Supplementary Table 4. Identifiers of the sequences used for VLDLR phylogeny inference.

Group	No. in fig.4B	Species	Gene name	Accession No.	Data source
VLDLR	1	human	VLDLR	NP_003374.3	NCBI
	2	chicken	VLDLR	NP_990560.1	NCBI
	3	western clawed flog	VLDLR	XP_002934223.1	NCBI
	4	spotted gar	VLDLR	XP_015195729.1	NCBI
	5	*C. milii*	VLDLRc1	XP_007890802.1	NCBI
	6	*C. milii*	VLDLRc2	XP_042202743.1	NCBI
	7	*C. milii*	VLDLRc3	XP_042202724.1	NCBI
	8	thorny skate	VLDLRc1	XP_032873693.1	NCBI
	9	thorny skate	VLDLRc2	XP_032874680.1	NCBI
	10	thorny skate	VLDLRc3	XP_032874679.1	NCBI
	11	whale shark	VLDLRc1	Rhity1003076	Squalomix†
	12	whale shark	VLDLRc2	Rhity1003075	Squalomix†
	13	whale shark	VLDLRc3	Rhity1003074	Squalomix†
	14	spotless smooth-hound	VLDLRc1	see Supplementary Data 1	This study
	15	cloudy catshark	VLDLRc1	Scyto0020726	Squalomix†
	16	cloudy catshark	VLDLRc2	Scyto0010396	Squalomix†
	17	cloudy catshark	VLDLRc3	Scyto0010397	Squalomix†
	18	frilled shark	VLDLRc1	see Supplementary Data 1	This study
CD320	-	human	CD320	NP_057663.1	NCBI
	-	western clawed flog	CD320	XP_002938609.3	NCBI
	-	spotted gar	CD320	XP_015220751.1	NCBI
	-	*C. milii*	CD320	XP_007909401.1	NCBI
	-	zebra shark	CD320	Stefa0023857	Squalomix†
LRP8	-	mouse	LRP8	NP_444303.2	NCBI
	-	chicken	LRP8	NP_990517.1	NCBI
	-	western clawed flog	LRP8	XP_004914072.1	NCBI
	-	spotted gar	LRP8	XP_015211098.1	NCBI
	-	whale shark	LRP8	Rhity1033667	Squalomix†
	-	thorny skate	LRP8	XP_032884011.1	NCBI
LDLR	-	human	LDLR	NP_000518.1	NCBI
	-	three-toed box turtle	LDLR	XP_024055786.2	NCBI
	-	western clawed flog	LDLR	XP_002942891.1	NCBI
	-	spotted gar	LDLR	XP_015204644.1	NCBI
	-	*C. milii*	LDLR	XP_042200596.1	NCBI

	-	whale shark	LDLR	Rhity1033440	Squalomix[†]
	-	frilled shark	LDLR	see Supplementary Data 1	This study
cyclostome	-	sea lamprey	VLDLR	XP_032826857.1	NCBI
VLDLR	-	sea lamprey	VLDLR	XP_032822198.1	NCBI
	-	inshore hagfish	VLDLR	Eptbu0022314	Squalomix[†]
	-	inshore hagfish	VLDLR	Eptbu0034890	Squalomix[†]
invertebrate	-	Florida lancelet	VLDLR	XP_006824968.1	NCBI
VLDLR	-	*P. flava*	VLDLR	pfl_40v0_9_20150316_1g8564	Simakov et al. 2015
	-	*S. kowalevskii*	VLDLR	XP_006824968.1	NCBI

[†]The sequence was obtained from the elasmobranch sequence archive Squalomix (https://transcriptome.riken.jp/squalomix/).

Supplementary Table 5. Oligonucleotide primers for VTG cDNA amplification.

Organism	Gene	Forward primer	Reverse primer
cloudy catshark	VTG2α	GGCTGCCGGAGAAAGGCTTG	AGCCAGCGCCTTGGTTCAGG
		CAGCCAGCGCGAGAATTGCC	TAGCCTCGCTGTGTGCCGTC
		GCCGATGCAGCAATGCCCAG	TGCGCAGGTTTGCCCTGGAT
	VTG2β	GCGACGTGCGAAGGATCAGC	TGAACACCCTTCCAGGCACCG
	VLDLRc1	TCCTTGCTCATCCTTTTGCT	GGGTCGATGAAAAGACTCCA
	VLDLRc2	AGTGATGAACCCTTCCGATG	CCAGCCGTGCCTTTATATCT
		TTGATGTGGAAAGCCAAACA	GTTGCAGCAGCTCATATCCA
	VLDLRc3	ATGAACTGTTGCAAACATTTGG	TACACCAGCCATGGTCAAGA
frilled shark	VTG2α	TGCCCATTGTACCAGTTGATTGGAGAG	TGCACCCTAGGCAGTTGAGATGAAATC
	VTG2β	GACCAGAAGCTCCTCATTCAGTTCCAG	GAAACAGTTCTGGAATCTCAGCAACGC
		TTTAAAGCATCGATGGGATATCTTGCAAATCACC	GCATCGATGGGATATCTTGCAAATCACCCGGC
spotless smooth-hound	VTG2β	GCAAGACACCTACCTCCTTAAGATCGC	ATCGACCTGGAGTTTCAGTGGGATACT

Supplementary Table 6. Sources of chondrichthyan genome and transcriptome sequences.

Organism	Data type	Accession ID	Data source
spotless smooth-hound	transcriptome	-	This study
cloudy catshark	genome	GCA_003427355.1	NCBI
white shark	genome	GCF_017639515.1	NCBI
whale shark	genome	GCF_021869965.1	NCBI
zebra shark	genome	GCF_022316705.1	NCBI
brownbanded bamboo shark	genome	GCA_003427335.1	NCBI
spiny dogfish	transcriptome	-	Chana-Munoz *et al.* 2017
frilled shark	transcriptome	-	This study
red stingray	genome	-	Squalomix†
thorny skate	genome	GCF_010909765.2	NCBI
small-eyed rabbitfish	genome	GCA_012026655.1	NCBI
C. milii	genome	GCF_018977255.1	NCBI

†The sequence was obtained from the elasmobranch sequence archive Squalomix (https://transcriptome.riken.jp/squalomix/)

Supplementary data 1.—Manually curated protein-coding nucleotide sequences of the genes analyzed in this study. See Supplementary table S1 for other sequences used for the phylogenetic analysis in this study.

> cloudy catshark VTG1

ATGATGAGGGGCATCATTTTTGCACTGGCGTTCGCTCTTGTGGGAAGCCAACAATATGAGCCTTCTTTCAGCCACGGCAAGACCTACGTCTATC

AGTACGAAGGGGTGATTCTGACCGGCCTGCCTGAAAATGGCTTGGCTAAGGGAGGTCTCAAGATAACCAGCAAAGTGCAAATTGGTTCAGTCGG

TCAGAGGAAACATCTACTCAAGATTATCTCACCTCAAATTCAAGAGTACAGCGGGATTTGGCCAAATGCTCAATTCATCCCAGCTCGGAAGCTA

ACTCGAAAGCTGAACGCACAACTGAGCAAGCCTATTGAGTTTGACTACAGCCATGGCCGAGTGGGAAATATATACGCCCAGGCAGACCTGCCAG

AAAATATCCTCAACATCTACAGGGGCATCCTGAATATGTTGCAGATCAGTATTAAAAAGTCGCAGAATATTTACGAGTTGCAAGAGAATGGAGT

GGAAGGCATCTGCCACGCAAGCTATGTAATTCAGGAAAACAAGAAGAGTGGGATTGTCACAGTCACAAAATCCAAGGATCTGAACAAGTGCCAG

GAGAAAATCTTCGAGAACCAGAGTTCAGCTTATACTCAGCTCTGTGAAACCTGCCAGCTGAAAGGTAAGAACCTGCGGAGTGTCTCCACCTACT

CATATGCTATCAGAAATACGGAAGGCGAGGCAGTGATTATCGAAGTCGTAAGTAAAGAGACACATCAATTCACACCATTTAATGAACTTGATGG

TGCGGCCATCACAGAGTCCAGGCAGCACCTCGTCTTCTTGGAAAGCAAAGAGCAGTCGCCACCCGTGCCAACCGAGCACCTGGCGAAGCGGGGA

ACTCTGCGGTATCAGTTTTCCAATGAACTGCAGCAGATGCCCATGCAGTTGACCAGACCTTCGAATAACGACACTAATAAGATTGCCACAGCTT

TGGAAAACCTGATTCAAATGAACCAAGAGAGGGCTCATCCCGACGCTCCGCGGAGATTCCTGCAGCTCATTCAACTCCTGCGTTCGGCAACTTT

GGAAAACCTTCAAAGCATTTGGGAAAAGAATGCACGCGCATTGGATCACAGGCGCTGGATATGGGATACTTTGCCGACCGCAGCAACCCCTGAA

GCAATTCAGTTCATCCAGACCAAAATTGAGGAGGGAGAACTCAGGAACGGGGAAGCAGCCAAGGCCTTGATTTTTGTGCTACATTCCATCAACG

CCGACTGCCATGGTGTGGATAATGCAACGGTCCTTCTGTCCAGCCCTTACATGCAGAGCGACCCCTTCCTTCGCAGGGTCACGTTGCTGGCTTA

CGGAACGTTGGTCAACAAATACTGTGCCACACTCCGGGTTTGCCCCGATGAAGCTCTCCGGCCACTCCACGAGCTCGTTGTTGAAGCTGGCAGC

AGAGGCCATGAAGATGAAACTATCCTCGGCCTCAAAGCCATCGGCAATGCAGGACAGCCGTCCAGTCTGAAGCGCATCCAGAAACTCCTGCCGG

GGTTTGGCACAGTCGCCGGCAGCGTCTCGAACAGGATCCACATTGAGGCCGTGCTGGCATTACGCAACATCGCCAAGAAGGAGCCACGGAAGGT

GCAAGCCATCACCCTTCAGATATTCATGAACAAACGGGCCCCTGGTAGCTTACGGATGAAAGCCTTCATCGTCTTGCTGGAAACCCAGCCATCC

TTGCCTTTAATTGTAATAGTTGCTGACACACTGTCCAGAGAGACCAACATACAAGTGACCAGTTTCGCTTATTCCTACATGAAGTCTCTAGCGG

GATCCTCGGAACCAGAACTCCAGTCGCTGGCTGCTAGTTGCAATATCGCCATCAAACGTTTGAACCAAAAGTGCGACGCGCTCGGTTATCGGTA

CAGCAAAGGCTTCCATTTTGGAACGTTTAAAGATAAGCTTTTAGCTGGAATCAATGCCAATGTTTACCTCATAAAGAGGTCAGAGGGCATTCTG

CCAACGACAGCCATCACCAACATCCACCTTTATGGCCTTGGAGTCTCCTCGGATTTTCTGGAGATCGGTATCCATATAGAGGGAGAATGGAGAA

AAAATCAGCCCCACCAGAGGGGGCCGAGGAATGAACGTATAGCGAGGAAGGTGCCTGGATGGAAATCCATACCCACAACTAAGCCACTCGCAGT

CGCCTGGATCAAGTTGTTTGGTCAAGAACTTGCTTTTGCTGAACTTCACCAGGAAGATTTACAAGAGCTGAAGAAAGAAAAAATTGAGAGATTA

CTGGCGAAGTTCATAGTCATGCTTCAGAATGGAGTAACAATGCACTGGACTAAACCCTTGCTGGCATCCGAGATTCGGCACATTGTGCCAACAT

CGCTTGGCCTGCCAATGGAGATGGCGTTTTATTACACCGTCGTTTCAGCAGCACAGGCCAAAGTGAAGTTCACTCTACGCTCTTCCAATTTGAC

CATGGTTCAACTCCTGAATACCAGCATTGAGACTGATGTCCAGTTCACCTCAAGCTCAGTCAAAGATGTTATTGCCGTTATGGGAATAAACACA

CCTCTGATTCAGACAGGAGTAGAAGTGCAGTTGAAAACGAGCGCCGTTATCCCTGTGAACTTCACCGCAAGAGTAAACTTGAAGAAGAGCAATG

TCAAAATTGAAAGACTCCCATGGCAACAGGAAGATCAGCTGTTTTCGGCTAGGTCTCGGGCATTTGCATTCGCAAGAAATATTGAAGATTTGGC

TGCAGAGAAAGTCACTCCACTGCTATCGAGAGAAGAGTTTCGATTAATGAACAGTGAATTGAGTTTAGTGAAGAATTCAACATTGGATCACCAG

GATGCGATGGAGAAAGTGTTGCCTCTTGCTATACCACGGGGATCTGTGTGCTCTGCTGAAGATACACCAGACGTACCAAGCCCAACAGTCCACC

AGGCTTGTACAAGCTCCAACACATTCGGAGTCGAGGTTTGTTACAAGACAAGTATGGAAAACACCGCTTTCACCACTGACTCTCCACTGTACAA

AATGGTTGGAGATAAATCGATTGAAGTCACAATCAAACCAGTTACCACATCGATTGCAATCAAAAAGTTACAAGTCGAATTCCAGTTGCACAGA
GGCAATCAAATCAGTGCCGGTGTTCATCACTTGATGAGGAAGAGTAATGGGAGTGACTCAGCTTTCTCAGAACCCACACCGCTCGACGGCAAAT
TAGCCCTTCTGAAATTGAAGAAACTCTTTTCTGGAAATGCTCGGCATCAGGATAGGCAAGAACACAGATATACCATGGGCAGCTCGACATCAAG
TTCCCAGACATCGGGCAGAAGGTCAACGCGTATCAGCAGAGAATCAAATGAAAATAAGAAAGAGTACACAGCACATCAGCGCAGAAACGTTCCA
GTACCAAGGACCAGAGAAATTGGGCATGGGAGTGACCATAAGAATGCTAAACGCAATCAGCGGGATCAGCGTAACAAGCACGAGCAACCTACGT
CATCAGGGTTGTTATCTTCAACTCAATCAAAGGGTGAGCATTATAGGCGTCCTCAGCAAGGGTTGTCGAAGTCAATGACTCATCATTCGATCTC
CTCCAGCTCACCATCAGCACAATTAAGGGAAGAATATGGTTCGCATCCTCAAAACAGAAGGTTAAGAACAAAGACTCGTCATTCGATCTCCTCC
AGCTCGTCATCAGCACAGTCAAGGGAAGAGGATGGTGCACGTTACCGTGAAGGATCATCAAGGAAAGTCGGTAACTATCGATTCTCCAGCAGTT
CATCTTCAGCTCAGTCAAGGCGCAAGAAAGGAACCACCCAGTCATCTCATCTCCGCAGCAGCGTCGAAAAGTGTAATGACAGCAACTGCAGGGA
CAAACACCTCGGCAAACCTGCCTACAGGCCCTCGGTCAGAAACGACACCTTGCCTTCAACTGCCTCCGTTGGCCGATCCAGGTCCATTTCGTCC
TCGGCCCAGTCGCACGCAAGGTACGGTGAAAGTGCTGAGCATCTAGCATCAAGCTCATCATCTTCGAGTGAATCTAGCTCCAGCCGCTCATCGT
CACAGCAGCCAAAACATCGGCGCAGCCAACACTCCGGGCAACAGAGATCTGGCTCTTCGTCAAGAGTTGAAGCTTCCAGTAGTTCCATGTCTTG
GCACCACAGGACGATAAACATAGGGCCATCCACAAAGCATCGCACCAGCACCAGAACATGTAAAAACGGTAAATGTATCCACAAGTATACCAAA
TCACGTTCAACCACTATTCGCCGATATGAATCAGACCGCAGTACCTGGATCTTCAACTCAAAATCAGCAGAATGGGAAGGAGCCAATGCAAACA
TCTTCCAGCTTCGTTTCAAGCCATCTGAAAGTAGCCTGTCAGAAAACAAAGGTCGATTAAGCTACGAGTCATCATCTGAACCCAGTTCGAGGTC
CAGAATGAGTTTCTCCAGCTCATCTTCGTCTTCATCTTCGTCACAGCAGTCCTTGTCCCTTGCAGACTCAGTGCCACCACTATTCTCATTGCTA
ACCCGAGCCATCACGGTGGATAACAAGGAGAAAGGTTACCAGACAAAGGCCTACATAGACAATTCAATGGAGCAGCGAGCAGTACAACTATTTG
TGGACGAACTTCAAGAAGGAGGCAGCTGGAGAGCCTGTATTGGTGCTGAGATGCCCAATGTGCACAGAGCAGTGGCATTATTGAAATGGGGGAA
AAATTGCCAAGATTACAAGATTGGAGCAAAGGCAACTACTGGTCACTTTCAGCATCATCCTGCGGTACTGGTCAAAGCACAATGGGACAAGATA
CCTCAAACCCTAAAGGAAACGGCAGCAATTGTGGCTGACCAGTTGGCAGGGATCGCCTTCATGCTGGGATTCTCGGAGAGACATCAGAAGAGTG
CTGCTCACCAGATTTCAGTGATTGCTGCCGCAACGTCTCAACGCACACTTGATGTTGTCGTGAAAACCCCCAAGCATGTATTTAGTCGGCAATC
GATTCAAATCCCAGCACCATTACCTTTTAATGTAAACTCTCCCTCAGTGCAGCAAAGAGGATTACTTGTCTTTGCGGATTTACCTGACATCATT
TCAGCAACATCCACAGCCGAATGCACAGTTGTGCAAAACCAATTCACCCCATTTACCAAGGATAGCTTTGAGTACCAAATGCCTGAAGGGTGTG
CTCATGTGTTGGTCCAAGACTGTACACCTGAACTGCAGTTCATCACACTAATAAGACGCAGTGCAGAATCGCTGGTTGTACAGCTGTATTTACC
TTATGGCGAAATAGAAATACAAGCCACAACAAAAGGAAAACTCCAGCTGTTCATCAACAGGACCAGGACGCCAATCGCAAGCCTTCCATTTTCA
GGTCTAAGATCCTTGGTCATTGAGAGAAGTGACAATGGACTGAAGATAAAAGCACCAGAACTCGGCCTTGAGAAGCTCTCCTTCGATGGCAAAG
AAATCAAGGTTGCCGTGGTGCCATGGATGGCTGAGAGCACATGCGGATTGTGCGGCCGAAGTGATTCCCAGAGGAGGAATGAGTATCCACAGCC
AAATAAACGCAGCACCAACGAAAGTCTCAAATTTGCTCACTCGTGGTTGCTGCCCGGCGAAAACTGCAAAGACGATTGTAAACTGATGAAAAGA
ACTGTGAAGCTGGAGAAATCCGTTAAGATACACGGACAGGAATCTAAATGCTACACCATTGACCCGGTTTTACGCTGTCAGGTTGGATGTTCAC
CAGTGAAAACAGCCCCAGTTGTCTACAATTTCCATTGCCTCCCAGCCGATTCTCATGCGAATCCGAGCGACGAGCAGCTGATATCCGCCAACTT
TGGTCAGAAGAGTGAAGATTTGACCGGTCCTGTTGAAGCCCATACTGCCTGTTCATGTCCTTCTGAATGTAGTTAA

> cloudy catshark VTG2β

ATGAGGGCTATCATATTCATGTTGATTGTATCCCTTGTGGGCAGTCAAAAGTTTAAGTATGAACCCAGTTTTACCGAAGGCATGATGAATGTTT
ACACGTACGAAGGTATTATTCTAACTGGACTGCCAGAGAGTGGCTTAAACAGAGCTGGTGTGAGAATTAATTGTGGAGTGAATATTGTTCCATT
GGGGCAGAACACGTACCTTCTTAAGGTCACGCACCCTCAGATTCAGGAGTACAATGGCGTCTGGCCCAGCGACCCCTTCGTCTCTGCACGTCGA
CTCACTCAGAAACTGGCTCCAGAACTGATGAAGCCCGTGAAATTTGAGTACAACAAGGGCCAAGTGGGAAAGATCCAAGCCCCAGCAGACTTAC

TGGAAGATATCCTGAACATCCACAGAGGAATCCTCAACATTTTTCAAATCACCATGAAGAAGTCACAAAATTTCTATGGGTTGCAAGAGGTTGG

AATTGAAGGTATTTGCCTCACAAATTACATTGTTCAGGAACACAAGAAAGCTCAACGGATTACGATCACCAAATCAAAAGACTTGAACAACTGT

CAGGAGAAGGTTATGATGTACACGGGATCAGCTTATGCAGATCTCTGCCCTGCCTGCCAACAGAGAAGTAGAAATATACGGGCATCTGCTACTT

CCACCCTTGTCCTGAAACCTACCGCAACTGGGGCAATTCTTCAGGAAGCCAAGGTTCGAGAAGTGCATCAGTTTACACCATTCCATGAACGTGA

GGGAGCCGCCATATTGGAAGCAAGGCAACACCTCACGCTGGTTGCTATCAAGGCTGCAGTAATACGCGAACTGCAACTTGAGTCTGTGGAACGA

GGAACATTGAAATATCACTTTGACAAGAAAATACTGCACAGACCAATAAAATTAATGAAACCCCAGAATGTGGAAAAAGTGATTCTGGAGACCC

TGAAAAACTTGGAATTGCACAACCAGGAGAAGGTCCATGCTGACACACCCGCTAAATTTCTGCAGCTTGTACAACTTCTTCGCTCCACAACAGA

TGAGACCATTGCTTCCGTATGGAGACATTCCGACAGCAGCCAGTTACGGCGCTGGATTCTGTTTGCACTTCCTGCTGTCGGAACTACTGGCGCA

CTGAGATTTCTCAAAATCAAAATTCAAAATTTGGACATCACCATGGTTGACGCAGCTCAGGCGTTAGGCGTTGCAATGCACCAAACCACAGCCA

ACCTTCAAAGCCTATTGATGGTCAGAGACCTATTTCAAATGCATCAGGTGCAGCAGTTCTCAATTCTTCGCCAGATCGTTCACCTTGGATATGG

CTCTATGCTTTTCAGATACTGTGCAGCGCAGGCCGCATGTCCTGATACTCTGCTGAAGCCACTCCACGACTTGCTGACTGCAGCTACTGCTCAG

GCTAATGAGGAAGACATCGCTCTCGGTCTCAAAGCGATCGGCAATGCAGGGCAGCCAGCCAGCATCAAGAATATCATGAAACTATTACCTGGAT

TCGGCACGGCAGCTGCCAGTATCCCACTGAAACTCCAGGTTGATGCGCTCATGTCGCTGCGCAACATTGCAAAGAAAGACCCAGCAAAGGTACA

AGCCATCACTATTCAGGTCTTCATGAATCGACGGAATCACCCTGAATTACGAATGTCTGCTTGTGCAATATTCCTGTGCACCAAGCCGTCTTTG

AACTCATTGTTAGTCCTAACTAACTCACTGTTAAAGGAGCCCAGTTTGCAGGTGGCAAGCTTTGCTTATTCGCAATTCAGATCGCTTGCAAGGA

GCTCACTCCCATCCCTCAGTTCTTTGGCGGCTGGCTGCAGTATAGCTGCGAAACTCTTAAGTCCCAGATTTGACCAGCTTGGCTTACGATTCAG

CCAAGTATTTCACCCTGACATTTTTAGCTACAAGCTGATGTCGGGACTATCTGCCAAAACCATCATAATGAACAATGTTGGGAGCCTCATCCCG

ACATTAGCAGCAGCCAGAGTGAGATGTCACACGTTGGGATCTGCCGCCAATCTTGCCGAGGTTGGATTCCGAATGGAAGGTCTCCAGGAGGTCA

TGACGAAAAGTCGTGCAGGAGTCAGACGAGTTCCTGATATGAGACAAATTCATCGTATTTTAAATGGGTTCCCAGACTGGAAATCCTTGCCAGA

AAAAGTACCGCTAGCTTCAGCGTACATGAAACTACTTGATCAAGAGATAGCTTTTGTGGAGTTCCGGAAGGACGATATTCGCAAAGCCATTCAA

TCAGTGACTGATACACACGGGAAACTCAGCACTTTGAGAAAGATACTCAACCGGCTCCAGAAACCAATTGAAATGCATCCTGCGGCAGCATTAC

TGACAGCTGAGCTACGACGCTTCGTCCCCACATGTGTAGGTCTTTCCATGGAGCTGTCCTTCCTGTCTGCTGCCGTGGCAAGAGCCAATCTCAA

CATTGATGCGAAAGTCCCTTCCTCCATCTCTTCCTTTTCTCAATTGCTCAATGCCAACATCCAGCTGAAGGTTCAGATAAGCCCAAGTGTGGCT

GTATACAGCAAAGCTATCATGGGAATAAATTCGCTCATCATCCAATCTGGTTTGGAATTCGAGGTAAAAATCCACTCCGCTTTCCCGATGGATA

TATCTGCAAATATAAATCTTCATGAAAGGAATTTGAAGATTGACAGTCCAGCACCTCAGGAGGAGAATCGAATTATATCTTTCACGTCAGAGGT

TCTCGCTGTTTCAAGAAATATTGAGAATCTGTCTGCAGAGAAATTGACTCCAGTTGTGCCTGAAGCAAAGGAACCGAGCATTGCAAATCAAAAA

TTCAAGTCATCCGGACATAGTCAAACAAATCCAGATCTTTGTTCAAGAATCATAACAGATGAAGCTGAATGTTATGATGAAGCACAGAATCCCG

CACCAAGGCCCTCTGTGGCTAACATTTGTACGAGGATGACTACCTTTGGCTTTGACCTGTGCCTGGATGCAAAGTCGGCAGATGCTGTTTTCAT

TCGTCATGGACCACTGCATAGATTGATGGGAGCGCACACTGCCAAAGTTTCAATCAGGCCAGTCCAGTCAGATACAAAAATTGAAAGGTTGGTG

TTGGAAGTACAGACAGGCCCCAAAGCAGGTTCCAAAATGATTCGAATCCTGGACATAGAGGAACCACTACCTGAGAGAATCCGTAGCCATACCG

GCCTGTTCAAAGAATATCGCTCACAGACCGGAATGAAAAACCAGACTTGGGCCAGCAGCTCCTCGTCCAGTTCGATTTCCAAAAGCTATTCAAG

AAGCAGGGCTACCGCTCAAAGGCCTTCATTATCGAGTGGCCGATCAAACAAGCGACACCACAACGTTGACCAGAAGCAAGATCAACCGGGTGGG

ATTTCCAGAAAGCGAAACGACAGCACAAAACACCCTCGCAGTTCCAGCTCCAGCAGATACATGTTTCCCGACAGCACTCGGGGAATAATCCAAC

AACTCATGGATATGGAGTTCAAATCAGCCGGAAGCAGTGAGACGACTCACAGGGGGAAACCAGCCAGGAACAAGCCACACTCCAGACTAAGACA

CAGACATGGTAGCTCGAGCAGTAGACAATCTTCTGAACAGCGTGATCTCATCAGGGACATTGGTACTCCCTCACTCATCGTTCTTGCTCGGGCT

AGAAGGTCTGATGGAATACAGCAAGGATATCAGCTCACTGGATCTGTGGAAAGCTCCCATGGCAGGCCTGAAATGCACCTGCGACTTGTCGATC

TGAAGGAAGACAGCATATGGAAAATGTGTGTGGATGCTGCTGTCCCTAAGCCACACAAAGCAATGATAATGTACAGATGGGGTGAAAACTGCCA

GACGTATAAGATGTCTTTTAAAGCATCGATGGGACATCTCGCAAATCACCCAGCTTTGAAAATTCGAACTAGGTGGTCGGAAATTCCTCGTGCG

ATGATAACCGGCGGAAGGATGATAGGATCTGGAGCTGCTTATCTGTTAGGATTTTCCAGTAGCTTTGAAGGTAACCCTTATCAGCAGATAACGC

AGCTGATAGCACTAACATCACCCCGGACCATTGATACAATTGTTAAACTACCCAGGTTTACTATCTATTATCAAGGCTTCGAGCTCCCTTTGCC

AGTCCGCGTCCAAACAATGGCTCCAATCATACGGAAACGCGGATTCAAAGGCATCACTGAAGTTGCACGTCTGCTTCTGACCATCAACCAGCGG

GAATGTATTGCTGAGAGTGAGCGAGTTGTTACATTCGATAGCAATGAACTGAAATATAAAATTGTTAATGATTGCCACTACGTCCTCACCAAAG

ATTGCTCACCAACTCCAAAGTTTGTTCTACTGATGCGACGTGCGAAGGATCAGCTGAGAAAGAGGGCAATCAAGCTGCTGATATCAGTGCCCAA

TATCGTAATTGAAGCATATCCTACAACAGATGGAATAAAACTCTTGGTGGATAACGTTGAAACCACTCTGAGTAAACAAGGAAATGTTATCCAA

AATCTTGTAACGATCCAACAGAATGGGACTGGAATTACTTTAGAAGCACCCTCAATCAATATTGACCAATTGTCCTTTGACGGAGACAGAATAC

AGATTGTACTTGATCAGATGATGAGTAAGACATGTGGTATTTGTGGACATAATAATGGTGAGCGTAAAATGATGAAACCAAATCAAGAGGAAGC

CAGAGATGTTGAGGATCTTTTTGAATCATGGACATATCCAGGGCAAACCTGCACAGATGATTGCAAAGTTAGACAAAATTTTGTGGAACTAGGA

AAAACTGTCAACGTTGAAGGGCAGGAATCCAGATGTTACTCAGTTGAACCAGTTCAACGGTGCCTGGAAGGGTGTTCACCCATTGAAACACTCT

CGCGCATTGTCAATTTCCACTGTGTACCAGCCAATCAACCTGTGGATGCAGCCGTCATATTCAGTTCCCGAAAGAAGTCTGTGGATACGAGTCA

CCCTGTGGATTCTCACACGGACTGTTTGTGTCGCTGTACAGAAATATGA

>frilled shark VTG1

ATGAGGGGGATTATTTTTGCACTGGCCTTTGTCCTCGTGGGGAGCCAACAAGTGCACCACGAGCCTGTTTTCAGTCAAAGGAAGACATACATTT

ACCAGTATGAAGGGATCATTCTGACCGGCCTTCCAGAAGATGGCTTGGCTAAGGGAGGTCTTAAGATAACCAGCAAAGTGCAAATTAGTTCAAT

GGGGCAGCAGAACCACCTCCTTAAGATGATCTCACCTCAAATTCAAGAGTACAGCGGGATTTGGCCAGACAATCAATTTATTCCAGCTCGGAAG

CTGACTCGGAAGCTGAGCGTGCAACTGAACAAACCTATAAAGTTTGAATACAGCCATGGCCGGGTTGGAAACATTTACGCCCCTCCAGACCTAT

CCGAGAATATTCTCAACATCTACAGAGGCATTCTGAACATCCTGCAGATCAGTATTAAAAAGGCGCAGGATATTTATGAGTTGCAAGAGAATGG

GGTGGAAGGCATCTGTCACGCAAGCTATGTAATTCAGGAAGATAAGAAGGCCGGACGCATCACTATCACAAAATCCAAGGATCTGAACCGCTGT

CACGAGAAAATCTCAGAGACCAAGGGTGCTTCTTATACTCACTCATGTGAAATCTGCCAACTGAAAGGCAAGAACCTGCGGAGTGTTTCCACTT

ACACTTATGCCATGAAAAGTACAGAAGACGGGGCCGAGATTTCCGAAGTCATAGGCCAGGAAACACATCAGTTCACCCCGTTTAATGAACTTGA

TGGTGCCGCCAGTACAGAGTCAAGGCAGCATCTTTTCTTGTTGGAACTCAAAAATCAGTCGCCATCCATGCCATCTGAGCCCATGGAGAAGCGG

GGGACTCTGAGATATCAATTTTCCAATGAATTATTGCAGATGCCAATGCACTTAGTCAAGACCTCACATAATGATACTCAGATTGCCGAAGTAC

TGGAAAACTTAGTTCAAATCAACCAAGAGAAAGCTCATCCTGACGCTCCGCATAAGTTCCTGCAGTTCATTCAGCTCCTGCGTTCGGCAAATTC

GGAAAACCTTCAAAGCATTTGGAAAAAGAATGCACCCAAACAAAACCACAGGCGCTGGATTTTGGATACCCTACCAACTACAGCGACACCTGAA

GCAATTCATTTCATCCAAAGGAGAATTGAGCAGGGGGAGCTCACACAGGTTGAAGCAGCTCAGGCTTTGGTTTTTGTATTATATTCCATCAAAG

CCGACTGCCATGGTGTGGATAATGCTACGATGCTTTTGTCCAGCCCTTACATGCAGAGAATGCCCTTCCTTCGCAGAATTACGTTGCTCGCTTA

CGGATCGTTGATCCAAAGGTATTGCATGACTCTCCGGACTTGCCCTGACGAAGCTCTCCAGCCACTCCACGAGCTCATCGTTGAAGCCAGGAGC

AGATCCCAGGAAGGAGAAATGATTCTTGGCCTCAAAGCGATCGGCAACGCAGGGCAGCCAGCGAGTATAAAGCACATCCAGAAACTCCTGCCGG

GATTTGGAAATGGCGCCAGCGGCATCCCGAGCAGAATCCAAGGGGAGGCCGTGATGGCCTTGTGCAACATCGCTAAGAAGGAGCCGCGCAAGGT

GCAAGGCATCACCATACAGTTACTTATGGACAAAAGGATCCATGCCGAAGTGCGAATGAGGGCTTTCATCGTCTTGCTGCAAACCAAACCATCC

TTGGCTTTAGTAGCAACCGTGACTGACTCGCTGGCCAGAGAGTCCAATTTACACTTGACCAGCTTCGTTTATTCCTACATGAAATCTCTAGCAG

GAACCTCGCTGCCCGATCTCCAGTCACTGGCCGCTACCTGCAACATCGCAGTCAAACGATTGAATCAAAGGTGCGACAGGCTCAGTTATCGGTA

CAGCAAAGGCCTGCATTTCGGCGCGTTTAAAGATAAGTTTTTGGCTGGGATCGATGCCAACTTTTACTTAATAAAGAGGTCAGAGGGTATTCTC

CCGACTGCAGCCGTGGCCAATTTGAAGGTTTATGGCCTTGGAGTCTCCACAGATTTTCTGGAGATCGGTATCCAAGCAGAAGGACTGCAACAGG

CCTTGTGGAGAAACAGTCCACCCTACAGGAGAGGATCGAGGAACAACAGCTCTCAACGCATTATGGGGAAGGTGTCTGGATGGAAACCAATGCC

CACAATTAAGCCCTTGGCGATCGCCTACATCAAGTTGTTTGGTCAAGAGCTTGCTTTTGTCGAACTTAACCAGAATGATATTCAAGAGGCTGTA

AAGTTAATGAACAACCAAGCAAGAAAGGACGGACTAATAAAGAAATTCATAAACCAGCTACAGCGTGGAATCACAACACAATTGACCAAACCCT

TGCTGGCAACCGAGATTCGGCACATTGTGCCAACAGCACTCGGCCTGCCAATGGAGCTGGCGTTTTACTATACTGTCGTCTCAGCAGCTGCAGT

ACAGGCCAAAGTGCGGTTCACACCAGTCCCTTCCGATTTTACACTACCTCAGCTCCTGAATACCAGCATTCAGCTTGATGCTCAGCTCACCCCA

AGTTCAGTCAAAGATGTTATAGCCGTCATGGGAATAAACACGCCTTTGATTCAGACAGGAGTGGAAGTGCAGTTGAAAACAAGCACTGTCATCC

CTGTGAACTTTACTGCGAGACTAAACTTGAAGAATAGCAACGTCAAAATTGAAACCACCCCATGGCAACAGGAGTATAAGCTGTTCTCTGCTAG

GTCACGGGCTTTTGCCTTCTCAAGAAATATTGAAGATTTGGCCGCAGCAAAAGTGACCCCAATGCATGCGTTTGGAATGGTAATCAGAAATTTC

AGATTAGCAAAGAATTCATCGGCAGACCACGAGCAAGTGATGGAGAGAGTGTTGCCTCTTACCATACCACGGGGATCTGTGTGCTCTGCTGAAG

ATACACCAGACATATCAAGCCCAACCGTCTACCAGGCTTGCGCGAAGGCCAACACGTTCGGATTTGAAGTTTGTTACAAAACCATTACAGAAAA

CATTGCCTTCGCCACCGACTCCCCGTTGTACAAAGTGTTTAGAGAGAAATCAATTGAAGTCGCAATCAAACCAGTTATTACACCAATCGCAATC

AAAAAGTTACAACTAGAACTCCAGGCGCAAACAGGGGACCAATTCATTGCAAAAGCAAGTCGCTTGATGAAGAAGAGTGACAGGATTGACACAG

AAACAACACAGCCTGAGGGTAAATTAGTCCTTCTGAAATTGAACAAACTCTTTCGTGCAAAAGATGAGCATCAGGCTAAAAGAGAACACAGATA

TACCATGAGCAGCTCAGCATCAAGCAGAAGGTCAACAGATTTCACCAGACAATCTGATGAAAATAAGAAAGAGCACACGAGCACTCGGCATCAA

TATGCACCAAAAGCAGAGAGCAGAGACGGCGATCATGGTAATCGTGGCAAGCACGGTCCTAATCAGGAATCACCGAGGCCAATGACACATCAAT

CGATCTCCTCTAGCTCATCAGCGGATCAGTCAAGGCATCGCCACAGCAAGCACACTGGACATGGGCTATTTAGCTCTTCAGCGAGAGCTGGCAC

CTCCAGCAGCTCATCATCTTGGCAGCGCAAGACAGTAAATAAATGGCCATCCACAAAGCATCGCACCAGCATCAGCGAAACATGTAAAAATGGC

AAATGTACCCTCAAATATAGCAAATCAAACTCAACCATTATGCATAGATCTGAGCTGGAAATTAGTCCCTGGGCCACCGACCCAAAATCAACAG

AACAGGGAGCAACCAATGCAAACATCTTCCAGCTTAATTTCAAGCCATCTTATGTTGACCTGTCACAAAACAAAGGGCAATTAAGCTTTGAGTT

ATCATCTGAATCCAGTTCGGAGTCCAGAATCAGATTCTCCAGCTCATCTTCATCTTCATCGCAGCAGTCCTTGTTCCCTGGAGTGCCAATATTC

TCATTGCTAATCCGAGCCGTAACGATCGATGACAAAGAGAAAGGCTACCAGGCAAAGGCGTACATGGACAGCTTAATGGAGCAGCAAGCAGTGC

AACTGTTTGTGGATGAACTTGAAGAAGGAGGCAGCTGGAGAGCTTGTGTTGACGCTGAGATGCCCAATGTGCACAGAGCAGTGGCAGTATTGAA

ATGGGGCAGAGATTGCCGAGACTACAAGATTGCAGCAAAAGCAACTACTGGTCATTTTGAGCATCATCCCGCCTTACTGGTCAAAGCACAGTGG

GACAAGATACCTCAATCCCTAAAAGAAACTGCAGAAGCGGTGGCTGACGAGCTAGCAGGGGTTGCCATCATGTTAGGGTTCTCCGAAAGGCATC

AGAAAAGTCCTGCTCATCGGCTTTGGGTGATTGCGTCTGCAACATCTCAACGGACGCTCGATGTTGTGGTTAAAACTCCAAAGCATATATTCAG

TCGCCGAGCCCTTCTCATCCCAGCACCATTACCTTTTGATGTAAGCTCCCCCTCAGTGCAACAAAGAGGATTACATGTCCTTACTGATTTACCT

GCCATGATTTCAGTAACATCCACAGCTGAGTGCACAGTTGTGCAAAACAAATTCACCCCATTTAGTGAGAACAGTTTTGAGTACCAAATGCCTG

AAGACTGTGCCCACGTATTAGTCCAAGACTGTACACCTGAACTGAAGTTCATAACACTGATAAGACGCAGTGCAGAATCGCTGTGTGTAGAGCT

GAGTCTGCCTTTCAGGATAGAAATCAATTCTACAATGATAGGAAACCTCCAACTGTTCATCAACGGAAGCAAGACGTCATTGGCAAGCCTTCCA

TCCTTGACCATTGAGAGAAGTGACAATGGGCTGATAATAAAAGCACCTCACGTCGGCCTTGAAAAGCTTTACTTTGATGGCAAAACAATCAAGG

TTGCCGTGACACCATGGATGGCCGGAAGCACGTGCGGCTTGTGCGGGCAAGGTGACTCCCAGGGGAGAAATGAGTATCAACAACCAAATAGACG

CAACACCAAGGAGATTCTCAAATTTGCGACCTCGTGGTTACTGCCAGGGGAAAACTGCAAAGACACTTGTAAACTGACGAAAAGAACTGTGAAA

CTAGAGAAACCGGTTAGCTTACACGGACAGGAATCCAAATGCTACTCAGTTGAACCAGTTTTACGCTGTCAGTTTATATGTTCACCAATCGAAA

CGGTCTCAGTTGCCTATGGCTTCCACTGCCTTACAGCCGATT

>frilled shark VTG2α

ATGAGGGCAATTATCTTCTTGTTGACTCTCGCCTTTGCGGAGAGTGAACATGACAAAAGATATGAACCCAGCTTCTCTGAAGGCAAGATGTATA

TTTACAAGTATGAAGGCGTGGTTCAGACTGGGATGCCGGAAAGAGGCTTGAACAGCGCTGGAGTGAAAATAAGCTCCAAAGTGATAATCAAGGC

AGTAGGACAACTGCAGCACATTCTTCAAATTGAAGATCCTCAGGTTCAAGAGCTCAATGGAATTTATCAAAAAGGCCGGTTCTCTACAGCCCAT

AAGCTGACAGAAAGACTGGCCCCGCAACTTACCAAACCCGTGAAGTTTGAGTACAACAAAGGCCGAGTGGGAAGTATTCAGGCTCCTGCGAACT

TGCCTGAAGATGTCCTGAACATCCACAGAGGAATCCTGAACGCTTTACAAATCACCATCAAGAAGTCGCAAAATTTCTACGATTTACAAGAGGC

TGGAATCGAAGGGATCTGTCACGCAAGGTATGTAATTCAAGAGGACAGGAGAAAGAACCGTGTGACTGTAACAAAAGCCAAAGACCTGACCAAC

TGTCAGGACAAGTTCGTGAAGCACACTGGTATGGCCTACAGCCAAATCTGCCCTCTCTCTCAGCAGAGAGGCAAGAATATACGTGCGTCTGCTA

CTTACACCTATGTCCTAAAACCTACAGCAGCTGGTGCAGTCCTTCAGGGAGCCACGGTTCGAGAGGTGCATCAGGCTACACCATTCCATGAACT

CGACGGAGTTATTAAGGTGGAGGCAAGACAAATCCTTATCCTGCAGCAGGTCACAACGGCATCGGCTAGTGTAGTAGCGGACTTGCAGAGTCGT

GGAGACTTGCAATACCGTAGTGATCGCAGCGTACTCCAGCATCCATTCAGGCTGATAAAGGACCAAAGTGTCATCACCCAGATAAAAGATACCT

TGAATCACATGGCCCAGCACAACGTGCAGGATATTCATGCTGATGCTCCATATAAATTCTTGCAACTTGTCCAACTTCTCCGTTTTGTTCAATA

CAAAGCTTTCTCAGACATTTGGAGACAGGTTGAAAAACAACCCGAACAGAGGCGTTGGTTCCTTGAGGCACTTCCTGCTGTAGCAACATATGAC

TCCCTGAGATTCCTCAAGAGCAAAATTGAAGAGGATCTCATCAACCGCGTTGAAACATTTCAGTTTTTAATTCTTGCAATGCACCAAGTCAAAA

CCGACCGCCAAGTTCTAAGTTTAGCTAAAGATATCCTTGATCTTACGCAAGTAAAACAATGCCAGTTTCTTCGCAGAGCTGCACACCTTGCATA

TGGTTCTCTGGTTTTCAAACACTGTGCAGATAAGTCAACTTGCCCCGACGATATACTGAAGCCACTCCATGACTTGCTCGCCGAGGCTAGCAGT

CGGCCCAACAGTAAGGACATTGTTCTCGGCCTCAAAGCGATCGGCAATGCAGGGCAACCATCGAGCATCAAGGCTATCACGAAAATGATACCTG

GATTTGGAATGATGGCTTCAAGTTTCCCACTGAAAGTCCGAGTTGATGCTATCATGGCACTGATTAACATTGCAAAGAAAGACTCGCGCAATGT

GCAACGAATCACTATGCAGATATTCTTCAATAAAAAGAATCAGCCTGAAGAACGAATGATGGCTTGTGAGGTACTGTTTGCTACCAAACCACCT

TTGACCTTGGTAGCCGCAGTGGCTAATTCACTGTTACAAGAGACCAGTTTGCAGGTGGCAAGTTTTACCTATACGTACTTGAGAACTCTGTCCA

GAAGTTCACTTCCATCCCTCAATTCGCTAGCTGCTGCCTGCAATCTAGCCCTGAATTTCTTGAGTGTCAAACTTGACCAGCTTGGTTACCGATT

CAGCAAAGTTTACAGAATGGATGCATTTAAGTACCAAATGATGGCGGGAGTATCTGCTAAAGCCCTTTTAATTAAGACTTCCAGCAGCATTGTT

CCAACAGCAGTATTGGCCAAAGTCAAAGGTCATGCTCTGGGAAGCTCAGCAGATCTTATAGAGGTTGGTTTTCGGGCAGAAGGTCTCCAGGAAG

TTATAATGAAAGTTCGTGCACCAAGCATTAGAAGAGCTGAGAGTAAAACTATACGACAGATCTTAAGTAAGATTATAAATTGGAAAGACTTGCC

AGAAGAGGAACCTTTGGCTTCAGCGTACATCAAACTGTTAGGGCAAGAGTTTGCTTTCGTTCAACTCAGAAAGGATGACCTTGAGCAAATTGGA

CAGACATTAACTGGTCCAATCAGCAGCCAATGGAAGAAATACATAGAGCAACTTAACAGTGGAGTCAATATCTATCCTGTGAAGGCGGTAGTGG

CAGCTGAGATGCGACAACTCACACCTACAACAGTTGGTCTGCCAATGGAACTGGGCATCGTTTCATCTGCTCTAGCAGCGTCCAAAGGAAACAT

TGAAGCCAGCTTCAAACCCCCCATTTTAAAATTTCCTGAGTTGTTAAATTCCAGGATTCAGTTGAAGGCCCAGCTCAACCCCAGTGTGTCCATA

CATACCATAGCATTTATGGGAATAAATAGTCCTTTCATCCAGTCAGGCATGGAACTCCATACAAATGTCCGTTTAACTATTCCTGTGGATGTAA

CTGCAAAGGTCACTTTAAAAGAGGGAAATTTGAAGATCGACAGTGCACCAGCTGAACAAGAGCACAGGATCCTATCTATGACTTCCCAGGTTTA

TGCTGTTTCAAGAAATGTCGAGAATTTGTCTGCAGAAAAAATGACCCCGATTTTACCTCTGACACACGAAAGTCACATTACAAGACAAAACTTT

CAGTCTTCATCCACGTCAGCTAGGGCAGCAGACGAGGGAATTGCTGCACATTCAGGCATGCTGCATGACCAAATTCTGAGCTCTGAAGACCAAC

AAAGGTCACGTGTCCCCAGTCGCTTGGCCTATCACACGTGTGTGCAAGCAGCTAAACTCGGGTTCCAAGCTTGCCTGGACGCCAAAATGGAAAA

TGCTCTCTCCTTCAAACATTGCCCATTGTACCAGTTGATTGGAGAGCATGTTTTAAATGTATCGATTGCACCAGTTAGTTCAGACGCACAAATT

GAAAAAATTCGGCTGGAAATACAGGCAGGATCCAAAGCACTGTCGAAGATGGTCCGACTCACAGACAAAGAGTCAAAGACGGAGAGAATTCATG

AAGACATGCGTCAAGGTGGAGTGAACCAAATCCTGAAGAAATCATCACAGACTAAACCAAACAATAATACTTGGATCGGCGGCTACTCGTCCAG

TTCAAGCGCGTATCAAGACTCAGGTAGGCCGTTCACCTCTCGAAGCTTTTCATCCTCAAGTGACCGGTCACACTCTCATCCGAAAAGAGGTGAT

GAGGAAGGCCAACGACAGAGAATAAGAGGAAACAGATACAGTGGTAGCAGCAGCAGCAGCAGCAGCAGTGCAGGATACAGCCCAAGCAAAC

ACATCCACAGAAGGACGTCCGGTAGGGAGATATCTTCTGAGAGAGAGAGAATAAACGTTATGAATTTTGAGTTCGAGTCAGCCGAAACAAGTCA

GTACACAGTCAGAAAGCAGACAGACCAAAGCAGTACCTCCATGTCAAGAGACACTTCAAAATTTATCATCAAGGAAAAACATATCTCTGGGTCA

AAGGAAAGGCGCATCAGCCGTGGCTCATCCATCCTTGGATCATCTGAACAGCGCTATTTGGTTGGCGACGCAGGCCCACCTATCGTGGTTGTCA

TTCTTCAATCTAAAAGGACTGATGACAGACTGCAAGGCTATCAGCTCACTGGATATGGGAAGATTTCATCTCAACTGCCTAGGGTGCACTTGCG

TTTGGTGGAACTGGACAGAAAAAGCAATTGGAGAATCTGTGCTGATGCGGCAATGCCCAGCTCACACAAATTATTGGCCTTAGCGAGATGGGGT

GAAAACTGCGAGAACTACAGGGCTTCCGTTAAGGTATCAAATGGTCAGCTTGCAAGCCACCCAGCCCTTAAGGTCAAAATCCAATGGTCCAAGA
TTCCTGAGTATCTGAAATATAATGCAAGATTCATTTGCAACTATATACCAGGATTGGCCTACAGCTTAGGATTTTCTCAAATGTACCAACGTAA
TCCTTCTCATCAGATTACAGCATTGGTGGCTGTAACAACTCCACGAACCATCGATGCAATCTTCAAACTGCCCAAGATGACAGCCTATTATCAG
GGTTTGCAGATCACCACAGAACTGCCCTTCCATGAAATAGGTGCACAATTGCAGGAAAGAGGTTTCGGCTGCATGACTGACATTCCGGTCCTGT
TTCTGACAATGAACCAACGTGAGTGTATTGCAGAAAATGAAATTGTTACGTCGTTTGACGGGGTACAGTTAAAATATCGGCTACCTAACGATTG
CTACCACATCCTCACCCAAGATTGTTCACAAGTTCCAAAGTTTATGTTATTGATGAAACGCGCTGAAATCGATAAGACAAAGAAGGCAATAAAA
TTGCTGCTGTCCTTGAATAATATAAGCATTGAAGCAATGCCTACGCAAAGTGGAATAAGACTGTTAGTTAATGGTGTAGAAAGACCTCTGGACC
AGCAAATTCCAAGTCTAAGTGATATTGTAACTATTCGGCAGAATGGCACCGGAATTACTTTAGAAGCTCCGTCAATCAATATCGACCTATTGTA
CTTTGATGGAGACAGAGTGCAGATCGTACTTGACCAGATGATGAGTAAGACATGTGGTATTTGTGGACTTAATAACGGTGAGAGAAAAATGATG
ATGCCAAATCAAGAGGAAGCCGGGAATGTTGAGGGTCTTTTCCAATCATGGATACGTTCAGGGAAATCCTGCAAAGATGACTGCAAAGTTGGAC
AACAATTTGTGGAACTAGGAAAGGTTACTGAAGTTGACGGGGTGCAAACCAAATGCTACTCAGTTGAACCAGTTCAACGGTGCCTAGCAGAATG
TTCACCAATTGAAACAGTCTCTTTAAACGTCGATTTCCACTGTGTACCAAGGGATTCCATAGTGTCTGACCTTACATTGTTCAATAAGAAGTCT
ACGGACATCAGACGGCAAGTGGATTCCCATAGTGATTGTTTGTGCAGGTGTACTGAAGCATAA

>frilled shark VTG2β

ATGAGGGGAATCATTTTCATGTTGATTGTATCCCTTGTGGGTAGTCAAAAGTTAAAGTATGAACCCAGTTTCACCGAAGGCATGATGCATGTTT
ACACGTATGAAGGTATTATTCTGACTGGACTTCCAGAAAGTGGTTTAAACAGAGCTGGTGTGAGAATATACTGTGGGCTGGAAATAGTTCCACT
GGTGCACAGCACCTTCCTTCTTAAGATCACAGACTTTCAGATTCAAGAGTACAATGGCATTTGGCCGAGGGAACCCTTCACCTCAGCACGTAAG
CTGACGCAGAAACTGGCTCCAGAGCTGACGAAGCCCGTGAAATTTGAATACAATAATGGCCGAGTCGGAAATATTTACGCCTCTGCAGACTTGC
CTGGAGATATTTTGAACATCCACAGAGGCATTCTCAACATTTTTCAAATCAATATGAAAAAGTCGCAAAATTTCTACGAGTTGCAAGAGGCTGG
AATTGAAGGTGTTTGCCTCACAAATTACATCATTCAGGAAAACAAGAAAGCCAACCGAATTACGGTCACCAAGTCGAAAGACTTGAACAACTGT
CAGGAGAAGGTCGTGACGTACAGTGGTACGGCCTACGCACGCCTCTGCCCTACCTGTCAACAGAGAGGCAGGAATATACGTGCGTCTGCTACTT
ACACCTACGTCCTAAAACCTACAGCAGTTGGTGCAATTCTTCAGAGAGCCACGGTTCGAGAAGTGCATCAGTTTACACCATTCCATGAACTTGA
CGGAACTGCTATAATGGAGGCAAGACAAAACCTCGTTCTGGTTAATACCAAGGCTGCAGTAATACATCTGTTGCAAGTTCAGTTTGTGGAACGA
GGAACTTTGAAATATCACTGTGACGAGGATTTATTTCGGAAACCGATACAATTAATGAAACACCAAAACGTGGAAAAGGCGATAATTGAAACCC
TGAAAAACTTGGCACTGCACAACCTGGAGAAAATCCATTCCAACGCCCCAGCTAAATTTCTGCAGCTTGTCCACCTTCTTCGTTCAGCACCAGA
TGAAACCATTGCTAACCTGTGGGCAAATGACAACAGAAACCAGTTCAGGCGCTGGATTATGTTTGCACTTCCTGCTGTAGGAACAACTGGTGCG
CTGAGATTTCTCAAAACCAAAATTCAGCAGATTGAAGTCACAAAGGCTGAGGCAGCTCGGGCTTTAGTAGTCGCAATGCATCAAATCACAGCTG
ACCTTCAAAGCCTATCGGTAGTCAGAGAACTACTCGTTATTGCTCACGTGCAACAGTTCTCCGTCCTTCGCCAGATTGTTTACCTTGGATACGG
CTCTATGGTTTTCAGATACTGTGCAGGGCAGTCATCTTGCCCCAGTGGTATACTTAAGCCGCTCCATGACATGCTCACTGAGGCTACTGCTCAG
GCCAATGAGGAGGACGTCGTTCTCAGCCTTAAAGCGATCGGTAATGCTGGGCAGCGAGAGAGCATCAAGCGGATTATTAAACTGTTACCTGGAT
TTGGGACAGCGGCTGAGCGCCTTCCACTGAAAATCCAAGTTGATGCTCTAATGGCGCTGCGTAACATTATAAGGAAAGAGCCAGGAAAGGTACA
AGCCATCACTATAAAGTTATTTATGAATCGAAGGAATCATCCTGAATTACGAATGTCTGCTTGTGCAGTATTTCTTTACACCGAGCCACCTTTG
AATTCTTTGTTAGTGCTAGCTAGTTCGCTGCTAAAGGAGACCAGTTTGCAAGTGGCAAGCTTTGCCTATTCACAGTTCAGATCTCTTGCGAGAA
GTTCACTCCCATCTCTCAATTCCCTGGCTGCTGGCTGCAATGTAGCGGCGAAACTCTTGAGTCCCAGCTTTGACAAACTTGGTTTCCAATTCAG
CCGGGTTTTTCATCCTGACCTCTTTAACTATAAGCTGATGGCAGGAGCATCTGCCAAAGTCATTCTAATTAACAATGCTGGCAGCCTCATCCCA
ACAGTAGCAGCAGCCAAACTCATGGGCCACGCTCTGGGAGCTTCCGCAGATCTTGTCGAGGTTGGCTTGCGGATGGAAGGTCTCCAGGAGGTCA
TAATGAAAAGCCGTGTATTAGCCAGGGGAGTTCCTGATATGAAACAAATTCAACGGATTATGAATATGTTTCCAGATGGGAAATCCTTGCCAGA

AAAAGTACCTCTCGCTTCAGCATACATGAAGCTATTTGGCCAAGAGATAGCTTTTGTTGAATTCAGAAAGGATGATATTCACAAAGCCATTAAA

TCAATCACTGGTGTACCAGACAAACACAGTACATTGAGGAAACTTGTCGACCGGCTCCGAAAACCGGTCGAATTGCGCCCTGACGGCGCATTAC

TGACGGTGGAGTTGAGGCGACTTGTACCTACGTGCCTGGGCCTGCCCATGGAACTGTCCTTGCATTCCGCTGCAGTCGCAAGGGCCACGCTCAA

TGTCGCAGCAAACGTCCCTTCTTCCATCTCTAACCTTTCTCAGTTGCTCAATGCCAACATTCAGCTGAAGTATCAGATCAACCCAAGTGTGGCC

GTATATACCCGAGCCGTCATGGGAATAAATACGCCCTTCATCCAGTCAGGCGTGGAACTCGAGGCAAAAATGCATTCAGCTTTGCCTGTAGATG

TATCTGCGAGGATAAATATCAAAGAAAGGAGTTTGAAGATTGAGAGTACACCGTCTCAAGAGGAGTGTCAGATAATATCTTTGAAATCTGAAGT

GTTTGTCGTTTCAAGAAATATTGAGAAATTGGCAGCAGCAAAATTGACACCGATTTTACCTGACATAAAAGAAGCTAGAATTACAAGTCAAAAA

TTCGTGTCACCGTGGCACAATCAGAAAAATGCACAGCTTTATTCAGGAATAGGAGCAGATGGAGCTGAAGGCCCTCAAGGACCACAGCTGCGAA

CACCACGGCCCTCTGTGTATAACACTTGTATGAGAACGACTAAATTTGGGTTTGAGGTTTGCCTGGATGCAAAAATGGAAAATGCTATTTTCGT

TCGACATAGTCCACTGTACAGGCTGATCGGAGTTCACACTGCCAAAGTATTAATCAGACCAGGTAAAGTCCATTCGGAGACAGAAATTGAAAAG

CTGGTGCTAGAAATACAGACAGGCCCCAAAGCAGGTTCAAAAATGATTCGGCTTCTGGAGGAAGAGGAACTCCTACATGAAAGAATTGATGGTC

GTTTAGTTCTGACAAAAGAATCCCGTTCACAGACTGGAAAGCAAAACCACACTTGGACCAGAAGCTCCTCATTCAGTTCCAGTTCCAGAAGTTC

CTCGGGAAGCAAGGTCACCTCTCGAAGATCTTCATCTTCAAGTGACCAATCGCTCAGCAGAGTTTACGAGAACGAAGACCGACAGGGTCGGGTG

TGCAGAAAGAAATTGAGAAACAACTGCACAAGTCAAGACTCTAGCTCTAGCATGTCATCCGCCAGTACATGGGCACAGGGAATAAACCAGGAGC

TTTTGGAACTTGAGTTCAGATCAGCCCGAAACAGTGAGTGCACATGCAGCCAACAATCCACTGGGACAAGGTCAAATTCCAGGTTAAGACACAG

ACAGAGCAGCAGCAGCAGCAGCAGCAGATCATCTGAGCAGCGTGATCTCATTGGAGACATAGATACTCCTGCACTCATCATCCTTGCTCAATCC

AGAAGGACTGACGGCAAGCAGCAAGGCTATCAGGTCACTGGGTCTGTGGAAAGTTCAGGGGGCAGGCCTAAAATTCACCTGCGCGCCGTCGAGC

TGGCAGAAGACAGCAGGTGGAAAATGTGTGTTGATGCTGCCATCCCTAAAGCACACAAAGCGATGATAATGTACAGGTGGGGTGAAAACTGTCA

GAGGTATAAGATGTCTTTTAAAGCATCGATGGGATATCTTGCAAATCACCCGGCTATGAAAATTAAAGCTGAGTGGTCCCAGATTCCTGATGCG

ATGATGTCCGCTGCAATAACGATTGGGCCAGGAGTTGCCTTTCTATTGGGATTTTCTAACAGATTGAAAAGTAATCCTTCTCATCAGATTACAA

CATTGGTCGCTCTAACATCTCCATGGACTATCGATACAATCGTTAAACTGCCCAGGTTTACAATCTATTATCAAGGCTTCGAACTCCCTTTGCC

AGTGCTCGTTCATGCAGTAGCCCCAATAGTACAGGCACGAGGATTCGAAAGCGTTGCTGAGATTCCAGAACTGTTTCTGACAATGAACCAACGG

GAGTGTATTGCAGAAAACGAGCGAGTTGTCACATTTGACAGCAACGAGTTAAAATATAAAGTACCTAATGATTGCCACTACGTTCTGACCAAAG

ATTGTTCACCTACTCCAAAATTTGTTTTATTGATCAGACGCGTGGATAATCAGCAGACAAAGAAGGCAATAAAGCTACTCATGTCAGTGCCCAA

TATCACCGTTGAAGCATATCCTACACAGGATGGAATAAAACTCTTGGTTGACAATGTAGAAACCACTCTGAGTAATCAAGCAAAAGTGATACAA

AATATTGTAACTTTTCGGCATAATGGCACTGGAATTACTTTAGAAGCTCCGCCAATCAATATCGACCTATTGTACTTTGATGGAGACAGAGTGC

AGATTGTACTTGACCAGATGATGAGTAAGACATGTGGTATTTGTGGACTTAATAACGGTGAGAGAAAAATGATGATGCCAATCAGAGGAGCCAA

AGATGTTGAGGGCCTCTTCCAATCGTGGATACGTTCAGGGAAATCCTGCAAAGGTGATCGCCCCGTGTATAGAACTCCCAGATTCGTAAGAAGC

CTGTCGACATGA

>spotless smooth-hound VTG1

ATGGTGAGGGGGATCATTTTTGCACTGGCCTTCGCTCTTGTGGGGAGCCAGCAATATGAGCCTTCTTTCAGTCAAGGCAAGACCTACATCTACC

AGTACGAAGGAATCGTTCTGACTGGCCTACCCGAAAATGGCTTGGCTAAGGGAGGTCTCAAAATAACCAGCAAAGTTCAGATTAGTTTAATTGA

TCAGAGGAAGCATCTCCTGAAGATCATCTCACCTCAAATTCAAGAGTACAGCGGGATATGGCCAAATGCTCAATATTTCCCAGCTCGGAAGCTG

ACTCGGAAGCTGAACGCACAGCTGAGCAAGCCCGTCGAGTTCGAATACAGCCGTGGCCGAGTGGGGAACCTATACGCCGATCCAGACCTGTCTG

AAAACATCCTCAACATCCACCGAGGCATCCTGAATATGCTGCAGATCAGTATCAAAAAGTCGCAGAATATTTATGAGTTGCAAGAGAATGGGGT

GGAAGGAATCTGCCACACGAGCTATGTAATTCAGGAAAACAAGAAGAGTGGAATTGTTACTATCACAAAATCCAAGGATCTGAACAAGTGCCAG

GAGAAAATCTCAGAGAACCAGGGTTCTGCTTATACTCAGCTATGTGAAACCTGCCAGCTGAAAGGTAAGAATCTGCGGAGTCTCTCCACCTACT

CGTACGCTATCAAACTTATAGAAAATGAGGCGGTGATTATCGAAGTGGTCAGTAAAGAGACACACCAGTTCACACCATTCAATGAACTTGATGG

CGCGGCCAACACGGAATCCAGGCAACACTTTGTCTTCTTGGAGAGCAAAAGTCAGTCGTCACCAAATCCAACCGAGCATCTGGATAGGCGGGGA

ACCCTGAAATACCAGTTTTCCAATGAACTGCTGCAGATCCCAATGCAACTGATCAGACCTTCAAATAATGACACTAATAAGATTGTCACAACAT

TGGAAAATCTGGTTCGGATGAACCGAGAGAGGGCTCACCCTGACGCTCCGCAGAAGTTCCTGCAGCTCATTCAGCTCCTACGCTCAGCAACTTT

GGAAAACCTTCAAAGCATTTGGGAAACCAGTGCACCCACATTAGATCACAGGCGCTGGATATGGGATACCTTACCGACTGCAGCAACCCCGGAA

GCAATTCAATTCATCCAAGCTAAAGTTGAACAGGGCGACCTCACGCAGCCGGAAGCGGCCAAGGTTCTGATTTTTGTGTTACATTCCATCAAAG

CCGACTGCCACGGTGTGGATAATGCAACGGTCCTTCTGTCCAGCCCCTACATGCAGAGACACCCCTTCCTTCGCAGGGTCACTTTGCTTGCCTA

TGGAACGTTGGTCAACAAATATTGCACGACTCTTCAAGTTTGCCCCGAAGAAGCTCTCCGGCCGCTCCACGAACTCGTTGTTGAGGCCGGGAGC

CAGGGCCACGAAGGCGAAACCATCCTTGGACTCAAAGCCATCGGCAACGCAGGACAGCCAGCCAGCCTGAAGCGCATCCAGAAACTCCTGCCGG

GGTTTGGCACTATGGCCAGCAGCGTTTCCAGCCGAGTCCACGGGGAGGCGGTGATGGCCTTGCGTAACATCGTCAAGAAGGAGCCACGCAAGGT

GCAAGCCATTACCGTTCAGTTATTCATGAACAAAAGGCTCCCCGCTGAATTACGAATGAAAGCTTTTGTCGTCCTGCTGGAAAGCAGGCCATCT

TTAGCTCTGATCGCAACAGTTGCCAACTCGCTGGTCAGCGAGGCCGACTTACAAGTGACCGGTTTTGCGTATTCCTACATGAAGTCTCTGGCAG

CATCTTCGGTACCAGAACTCCAGCCACTGGCTGCCAGTTGCAACATTGCCGTCAAACATTTGAACCAAATGTGCAACGCGCTCAGTTATCGGTA

CAGCAAAGGCTTGCATTTCGGAGCGTTTAAAGATAAGTTTCTGGCTGGAATCAATGCCAATGTTTACCTCATGAAGACGTCAGAGGGTATTCTA

CCAACCACCGCCATAGCCAACTTCCATCTTTATGGCCTTGGAGTCTCTTCGGATTTCCTAGAGATCGGCATCCAAGCAGAGGGACAGTGGAGCA

AAAGTCAGTCCTATCTGAGGGGACCGAGGAGTGAACGTCTAATAAGGAAGGTGCCTGGGTGGAAATCAATACCAATGATTAAGCCCTTCACAGT

CGCCTACATCAAGTTGTTTGGTCAGGAATTATCTTTCGTTGAATTTCACCAGAGCGATTTACACGAGCTAAAGAAAGAAAAGATGGAGAGATTC

CTGAAGAAATTCGCAGACATGCTTCAGACTGGAATGTCAATACGCTGGACCAAACCCTGGCTGGTATCCGAGATTCGGCACATTGTGCCAACGT

CACTTGGCCTGCCAATGGAGATGGCGTTTTATTACACTGTCGTTTCAGCAGTACAGGCCAAAGTGAAGCTTACTCTCGGTTCTTCCAACTTTAC

AATGGATCAGCTCCTGAACACCAGCATTCAGAGCGAATTTCAGTTCACCTCAAGTTCGGTCAAAGATGTTATTGCTATTATGGGGATAAACACC

CCTCTGATTCAGACAGGAATTGAAGTGCAGTTGAAGACAAGTACCGTGCTACCTATGAACTTCACGGCAAGAGCAAACTTGAAGAAGGGAAACA

TCAAAATTGAAACACCCCCTTGGCAACAGGGAGATCAGCTGTTCTCTGCTAGGTCACGAGCTTTTGCATTCGTAAGAAATATTGAAGATTTGTC

TGCAGAGAAAGTCACTCCACTGCTATCAAGAGATGGGTTTCGATTAATGAACAGAGAATTGAGTTTAGCAAAGAATTCAACAATGGATCAAAAG

GGAGTGCTGGAGAAAGTGTTACCTCTTGCTCTACCACGGGGATCTGTGTGTTCTGCTGAAGAGGCGCCAGATGTACCAAGCACAAAGGTCCAGC

AGGCTTGTGCAAGCTCCAACACATTCGGAGTCGAAATTTGTTACATGGCCAGTGTGGAAAGCGCCGTCTTTGCCAGTGACTCCCCACTGTACAA

AATGGTTGGAGATAAATCAATTGAAGTCACAATCAAACCAGTTTCCACACCAGTTGCAATCAAGAAGTCACAAATCGAATTGCAGTTGCACGGA

GGGGAGCAAATCAGTGCAAGAGTTCGTCACTTGGTGAGGAAGAGTAATGTGACAGACACGGATTTCTCAAACACATCTCACGAAGGCAAATTAG

CCCTTCTGAGAATGAAGAAAACCTATCCTAAAGATGGTCAGCATCAGGAAAGGCCGGAACACAAATATACCATGAGCAACGTGGCATCAAATTC

CCAGGCATTGGGTAGGAGGTCAACAAGCGTCACCAGAGAGTCAAGCAAAAATAAGAGAGATCACATGAAGAACAAGCACGGAGGTTCACCCATA

CCAGAGACCAGAGACACTGGGCACAGGGCTCAACACAGGTCTGATAAACGCAATAGGCAGGATGAACGCAACAAGCACGAGCAACCTATGTCCT

CAAGATTATTATCTTCAACTCAGTCAAAGGACAAGCATTATAGGCACACTCAGCGTGGATCATCAAAGCCAATGACTCATCATTCAACCTCCTC

TAGTTCATCTTCAGCTCAGTCAAGAGAGGAATATGGTTCATATCCTCAGAATAGAAGATTAAGACCAAAGGTTCATCAGTCATCATCACAGTCA

AGGAAAGACATTGGTGCAATTCTTCATGAGGGATCATTAAGGAAATTGAATAACAGTCAGTTCTCCAGCAGTTCATCTTCAGCCCAGTCGGTGC

GCAGAAAAGGAATCACTAAGTCTCCACATCACTCCATCAGCACCGAAAAGTGTGAGGGTGGCAACTGTAGGGACAGACAGCTCGGCAAACCTGC

CGCCCGGCACTCGATCAGAAACACCTCCTTGTCTTCAACTGCCTCCATTAGCCGGTCCAGGTCCCATTCATCCTCAGCCCAGTTGCATGCCAAG

TTTGGTGAAAGTGCTGAGCATGCGTCATCAAGCGCATCATCTTCGAGTGAATCAAGCTCTGGCCGCTCATCGTCGGAGCAGTCCAAACGTCGAC

GCAGCAAACACTCTAGACAACAGCAGTCTAGCTCTTCATCAATGGTTGACACTTCCAGTAGTTCCATGTTTTGGAACCACAGGACCGTAAATAC

AAGGCCATCCACAAAGCATCACACCAGCACCAGAAAATGTAAAAATGGTAAATGTATCTACGAGCATGCCAAATCACGCTCAACCACCATACAC

AACCATGAGACAGACCGCAGCACTTGGATCTTCAACTCAAGATCAGCAGAAAAGGAAGGAGTCAATGCAAACATCTTCCAGTTTCGCTTCAAGC

CATCTGAAATTAGCCTGTCACAAAACAGAGGTCGATTCAGCTTTGAGTCATCATCTGAATCCAGTTCGGGATCCAGAATGAGTTTCTCTAGCTC

ATCTTCATCCTCATCCTCATCTTCGTCAAAGCAGTCCTTGTCCCTCGAAGACTCTGTGTCACCAATATTCTCGATACTAACCCGAGCCATCACA

ATTGATAGCAAAGACAAAGGGTACCAGGCAAAGCTCTATGTGGACAAGTTCATGGAGAAGCGAGCATTACAAATGTATGTGGATGAGCTTCACG

AAGAAGACAGCTGGAGAGCTTGCATTGGTGCTGAGATGCCCAACGTGCACAGAGCAATGGCCTTATTGAAATGGGGCAAAAATTGCCGAGACTA

CAAGATCGCGGCAAAGGCAACGACTGGTCACTTTCAGCATCATCCTGCAGTACTGGTCAAAGCACAATGGGACAGGATACCTCAGTCCCTAAAA

GAGACTGCAGGAATCCTGGCTGACCAGCTGGCAGAGATTGCCTTCATGTTGGGATTCTCGGAGCGACATCAGAAGAGCCAAGCCCATCAGATTT

CAGTGATTGCTGCTGCAACATCTCAACAAACAATTGACATTGTCGTGAAAACTCCAAAGCATATATTTAGTCACCAGACTCTTCGCATCCCAGC

ACCATTACCTTTTAATGTGAACTCTCCCTCGGTGCAGCAAAGAGGTTTGCTGGTCTTTGCGGATTTACCTGCCATGATTTCTGCAAAATCCACA

GTTGAATGCACAGTTATACAAAACCAGTTCACCCCATTTACCAAGGACAGTTTTGAGTACCAGATGCCTGAAGGCTGTGCTCACGTGTTGGTCC

AAGACTGTACACCTGAACTAAAGTTCATCACACTAATAAGACGCAGTGCAGAATCGCTGTTTGTACAGCTGCATTTGCCTTCGAGTGAAATAGA

AATGCAATCGACAACAACAGGAAAAATCCAACTGTTTATCAACGGGACCAGGAAGTCAATCACAAGCCTTCCATTTACAGGCCCAAGTTCCTTG

GCCATCGAGAGATATGACAATGGGCTGAAGATAAAAGCACCAGAACTCGGTCTCGAAAAGCTCTTCTTCGATGGCAAAGAGATCAAGGTTGCCG

TGGTGCCATGGATGGCTGAGAGCACATGCGGATTGTGTGGGCGAAGCGATTCCCAGAGGAGGGATGAGTATCAACAACCAAATAAACGCAGCAC

CAGTGACATTCTCAAATTTGCTCACTCGTGGTTACTGCCAGGGGAAAACTGCAAAGATGCTTGTAAACTAATGAAAAGAACTGTGAAACTGGAG

AACCCCGTTAAGATACACGGTCAGGAATCTAAATGCTACACGATTGACCTGGCTTTACGCTGCCAGACTGAATGTTCACCAGTGAGAACAGCCC

CGGTTGTCTATGGCTTCCACTGCCTTCCAGCTGATTCTCAAGCGAATCCAAGCGACGAACAGCTGGTGTCGGCCAACTTTGGTCAGAAGAGTGA

AGACGTGACAGGCACAGTTGAAGCCCACACCACCTGTTCATGCCCTTCTCAATGCAGCTAA

> spotless smooth-hound VTG2α

ATGAAAGGCTTTATCTTCTTGTTGGCACTCACCTGTGCGGGGAGTGAAGATTTCAGCAGATATGATCCCAACTTCTCTCAAACCAAGATGCATA

TTTACAAGTATGATGGTGTGATTCTGACTGGGCTGCCAGAGAAAGGCTTGAATAGAGCTGGTATGAGAATAACCAGCAGAGTGAAGATCTGGGG

ACTAGGATCAAGCCAGTACCTTCTACAACTTGAAAATCCTCAACTTCAAGAGCTGAATGGAATTTGGCCCAAGGACCCATTTTCCTCACTCCGC

AAGTTGACAGAAAGATGGACCCCACACCTGACCAAACCAGTGAAATTTGTGTATTTCAAAGGCCGAGTGGGAAATATCTACACCTCAGAAAACT

TCCCTGAAGATATTTTAAACATCCACAGAGGAATCATCAACATTTTCCAAATCACCAACAAGAAATCCCAAAACTTCTATGATTTACAAGAGGC

TGGAATTGAAGGAATCTGCCATACAAGATATATAGTTCAAGAGGACAAGAGAAAGGAACGCCTGGTCATAACAAAATCTAAAGACTTGACCAAT

TGCCAGGAGAAGGTTTTGAAACAAACGGGTACAATCTACACGCAACTCTGTCCTTCCTGTCAGCAGAGAGGCAGGAATATCCGTGCATCTACTG

CTTCCACCATCGTCCTGAAACCTACAGCACTTGGTGCAATTGTTCAGGAAGCTAGGGTTCGAGAAGTGCATCAGTTTACACCGTTCCACGAACT

TGATGGGACTTTTCGATTGGAGGCAAGACAAAGCCTTATCTTGGAGAAGATCACATCAGCGCAGATGGAACAAATCCCAGACATGAAAAGTCAA

CGAAGTTTGCAGTACCGCAGTGAGAGAAATGTTCTCCAGCAGCCATTCAAATTGCTGAAGGACCAAAGTGTGATCACCCAGATTAAAGACACCT

TGAATCACATGGCGCAGCACAATGTGCAGGATATCCATATGGATGCTCCAATGAGACTCCTGCAGCTTGTGCAGCTTCTCCGTTTTGCTCCACA

CAGAGCCTTCTCTGAAATTTGGAACTGGGTTAAAACACAACCTGAACAAAGGCACTGGTTACTTGAGGCAATCCCCGCTGTCGGGACAATAGAA

ACGATGAAATTCATCAAGAGCAGAATTCAAGAAAGTGAAAAAATCCCCTCGGAAATATTTCAGGCTTTAATTCTTACATTGCACCAAGTCAAAA

CCGACCGCGATGTTCTTGCTGAGGCCAAAGAAATCATAGACCTAGACCAAGTTAAACGTTGTCAGCTGACTCGCAAACTTACAATTCTTGCATA

TGGTTCCATGGTCTTCAGACACTGCGCAGAGAAGCCAACTTGTCCCGACGACATATTGAAACCCATCCACGCTTTACTGATTGACGCCAGCAGT

CGGCCCAATGACGAGGACATCGTCCTGGGTCTCAAAGCGATCGGCAATGCGGGACAGCCAGCCAGCATCAAGAATGTCGTAAAGCTATTGCCTG

GATTCGGCATAGCCGCTCCTAATTTCCCACTGAAAGTCCGGGTTGATGCTATCATGGCGCTATGGAACATTGCCAAGAAAGACCCAAAGACTGT

ACAACGTCTCGCTGTACAGACGTTCCTCAACCGAAAGAACCATCCCGAAGAGCGAATGATGGCCTGTGCGGTTCTGTTCGCTACCAAGCCACCT

CTAACTTTAATATCTATGGTAGCGAACTCACTGCTCACCGAGACCAGTCTGCAGGTGGCCAGTTTCACCTATTCGCATATTAGAGCTCTGTCTC

GAAGTTCACTCCCATCTATCAATTCGATGGCTGCTGCCTGCAATCTTGCACTGCACATCTTAAGTCCCAAACTTGAGCAACTTGGCCATCGTTT

CAGCAAAGTTTACCGTGTGGACACATTTATCTACCGGATGATGGCAGGAGCATCTGCTAAAGCCCTTTTCTTTAAGACTTCCAGCTGCATCATT

CCCACAGCAATGCTGGCCAAAGTCAGAGGTCATGCTCTGGGAGGCTCCTCAGACCTGATAGAGTTTGGCTTACGGGCAGAAGGTCTCCAGGAGG

TCATGATGAAGGATCGTGCAGGAGACCTTAGAAAAACTGACAGTAGATCTATTCGGCGCATCTTAAGCAAGTTTATAAACTGGAAGGAAGTGCC

AGAAGAGAGACCTTTGGCTTCTGCCTACCTCAAACTGTTTGGACAAGAATTGGCGTTCGCTCAACTGAGAAAGGAAGACCTTGATATAATTAAA

CAGAAAATTTCTAGCCCAGTCGACAGTTACGTGAACAAACTTAAAGATGGAATCAGTTTCACTCCTTCCAAGGCACTGATGGCAGTTGAGATGC

GTCATGTTGTACCTAGTGTTGTTGGTCTGCCAATGGAACTGGGCATCACTTCAACAGCTGTAGCACTCTCCAATATAAAAGTTGATGCCCGCTC

TGACCCTCCCATTTCTAAACTTCCTCAGTTCTTCACTTCCAGGATTCAGCTGAATGCTCGCCTCAACCCAAGTGTGTTCATACATATCAGGGTA

TTCATCGGAATCAGTATGCCTCACTTCCAGTCAGGTGTGGAACTCCGAGTGAATGTCCGTTTACTTATTCCCGTGAGCATAACTGCAAAGATTA

ACTTAAAAGAGGGGAACTTCAGGATTGACACTTCCCCAGCCGATAAGGAGAGCAGGATTCTATCCATGACTTCGCAGGTGTATGCAGTTTCAAG

AAATGTTGAAGATTTGTTTGCGGAAAGAATAACCCCGATTTTACCTTCGACTCCAGAGAGACGCATTTCAACACAACGTTTTAGATCTTCAGCA

GGGTCATCCCCTGGCACAAGCGCTAGAATTGCCTCACATCTGTCTCCAGAAACACTGTCTGACCAAGTGCCATGTTCTGAAGATGAGCAGAAGC

CGCATGTTCCCAATCGCTCATCCTATCGCTCATGTGCCACAGTAACGGAATTCGGCTTCAAAGCTTGTGTGGACGCCAGAATGGAAAACGCTCT

TTCCATCAAACATAGCCCATTGTACCGACTGATTGGCGAACACACTTTTAACGTGTCGATTGCACCAGTGAGTTCAGACCCAGAAATTGAAAAG

ATTGTGCTGGAAATACAAGCAGGATCCAAAGCATCTTCGAAACTGATGCGACTTTCCGACAAACAGTTAGGACCAGACAGAATTCGCAGCAGTG

CCGAACGCCTGAGGCTGATGACAAGAGGTTCAAAGCAGAGAATGTACAACACCACTTCGTCCAGTTCCAGCTCCAGCTCCCGCAGCTCGTCGCG

ACAGTCAGGAAGATTCAGCACTCGAAGCAGACCCCATTCAAGTAGCTCATCGGAATATACTCGGGGCAGAGCTTCTGCAAGAGCCTTCCCCAGT

GGAACAAGAGTCAGCAAGAGGAAAGGAAGCATGAGTAGCAGCAGCAGCAGCAGCAGTGGCAGCAAGAGAAGCTTCAGGCACTCCCAAAAAT

ACAGAAGCAGACACATCAGTGGACCTTTGCACAGTGGCAGGGTATTGAGTAGTGGGTTTCAGACAGTAAGCCGGCAGCTCTTGGATCTTGCGTT

CAGGCCATTGCAAGAAAGCCGGTACACAGTCCGAAGGACATCAGCAGGAATTATCCGTGCTTCAACACAGCGCTCTGGATCAAGCCGAATTACC

AAGATAGGACGTGATTCTGGCTCAAGGGCAAGTCATGCCAGCCATGGTTCATCCATCCTTCGATCATCTGAGCAGCGCTATTTGATCGGCAGAG

CGGGTGCACCTTCCTTGGTTGTCCTCCTTCGATCTCGAAGGACCGACGGCACACAGCGAGGCTACCAGCTGACTGGATACGGGAGGATGGATAC

TCGGCTGCCTAGTGTACACTTGCGTGTGGTGGAACTGGATGAGAGGAGCAATTGGAGAATCTGTGCCGACGCAGCAATGCCTAGTTGGCACAAA

GTATTGACACTGGCGAGATGGGGTGAAAACTGTGAGAGGTACAGAGTCTCCGTTAAGGTATCCAATGGACAGCTTGCAACTCACCCAGCAATTA

AGGTCAAAATGCAATGGTCCAGGATAAACGAGATCCTGAAATATAATGCCAAAATGATTGGGGACTATATTCCAGGATTGGCTTATGCGTTAGG

ATTCTCTCAAACCTACAGACGTAATCCTTCACGGCAGATTACAGTACTGGTAGCTCTAACATCTCCAAGCACAATTGATACAATCATCAAACTG

CCCAAGATGACAGCCTATTATCAGGGTTGGCAGATCCCCACATCATTGCCCATGCACTCAATAAGTGTGCGGGTGCAGCAGAAAGGATTCAGCA

GCATAGCGGAAATTCCAGACCTGTTTCTGACAATCAACCAACGTCAGTGCACTGTAGAAAATGAGCTGGTTCGGTCCTTTGATGAGGACGAATT

GAAGCATTCATTTACCAATAGGTGCCACTATGTCCTGGCCCAAGACTGTTCAGCATCTCCAAGATTTATACTGATGATGAGACGTGCTGAAATT

GATCCGAGTCAGAAGGAAATAAAACTGGTGCTGGCTTCGAATAATGCGATTATTGAAGCAATTCCTACACAAAGTGGAATAAAGTTTTTAGTTA

ATGGTGCAGAGAGAGACTTTAACCAACAGATTCCAGAGGTACCTGCAGTTCTCGTTCAGAAGGAAGGTGCCGGAATTATTTTCGAAGCATCATC

AATCAATATTGACCGATTGTACTTCGATGGAAAGAGAGTGGAGATTGTACTCAATCAGATGATGAGTAAGACATGTGGTATTTGTGGACAAAAT

AATGGTGAGCACAAAATGATGAAGCCCAATCAAGAGGAAGCCAGAGATGTTGAAGATCTTTTTGAATCGTGGACATCTCCAGGGCAATCCTGTA

CAGATGACTGCAAAGTTGGACGAGAATTTGTGCAATTTGGAAAATTAGTCGAATTTGAAGGACTGGAATCCAAATGTTTCTCGGTTGAACCAGT

TCAACGGTGCCTGGAAGGATGTTCACCCATTGAAACACGCTCTCAGATGGTCAACTTCCATTGTGTGTCATCCGATTTCACTGTGAGGGACAAT

ACAATGTTCAGTAGGAAGTCTCCAGACGTGCGACTCTCAGTAGATTCCCATAGCGACTGCATGTGCAAGTGTGCATAA

> spotless smooth-hound VTG2β

ATGAGGGCTATCATTTTCATGTTGACTGTGTCCCTCGTGGTTACTCTTCCTGGCAGTCAAAAGTTAAAGTATGAACCCAGTTTCACCGAAGGCA
TGATGGATGTTTACGAGTATGAAGGTATTATTCTAACTGGGCTGCCAGAAAGAGGTTTAAACAGAGCTGGTGTGAGAATTAACTGTGCAGTGAA
TGTTATTCCTCTGGGGCAAGACACCTACCTCCTTAAGATCGCACACCCTCAGATTCAGGAGTACAACGGCATTTGGCCAATTGATTCCTTCACC
TCAGCACGGAAACTTTCGCAGAAACTGGCTCCAGAGCTGATGAAACCGGTGAAGTTTGAGTACAGCAAAGGCCGTGTGGGAAAGATCCACGCCC
CTGCAGACTTGCAGGAAGATGTCCTGAACATCCACAGAGGAATCCTCAACATTTTCCAAATCACCATGAAGAAGTCACAAAATTTCTATGGGTT
GCAGGAGGCTGGAATTGAAGGTGTTTGCCTCACAAATTACATCGTTCAGGAAAACAAGAAAGCTCAACGAATTACTATCACCAAATCAAAAGAC
TTGAACAATTGTCAGGAGAAGGTTATGATGTACACTGGTTCCGTGTATGCAGATCTATGCCCTGTCTGCCAGCAGAGAAGTAGGAATATCCGTG
CATCTGCTACTTCCACCCATGTCCTGAAACCTACAGCATCTGGTGCAACCCTTCAGGAAGCCAGGGTTCGAGAAGTGCACCAGTTTACACCGTT
CCATGAACGTGAGGGAGTCGCTGTATTGGAAGCAAGGCAACATCTCAGGCTGGTTACTATCAAGGCGGCAGTAATACATGAGCTGCAACTTAAG
TTTGTTGAACGAGGAACATTGAAATATCGCTTTGATGAAAATATACTTCACAAACCAATAAAATTAATGAAACCCCAGAATTTGGAAAAACTGA
TCCTAGAAACCTTGAAAAACTTGGAATTGCACAATCAGGAGAAGGTGCATTCTGACATGCCCGCTAAATTTCTACAGCTTGTACAACTCCTTCG
TTCCACAACAGAGGAGACCATTGCTTCCGTGTGGAGAAATTCCTACAGCAACCAGTTCCGGCGTTGGATTCTGTTTGCACTTCCTGCTGTCGGA
ACAACCAGTGCACTGAGATTTCTCAAAATCAAACTTCAGAATTTGGACGTCACTATGGCTGATGCAGCTCAGGCTCTGGGTGTTGCAATGCATC
AAATCACAGCCAACCTTCAAAGCCTAGCCATGGTCAGAGAACTATTTCGAATGCCTCATGTGCAGCAGTTTGCCATTCTTCGCCGGATTGTTCA
CCTTGGATATGGCTCGATGGTTTTCAGATATTGTGCAAAGCAAGCCATATGCCCTGAAACTCTCCTGAAGCCGCTCCACGACTTGCTTTCTGCA
GCTACTGCCCAGGCCAACGAGGAAGACATTGTTCTGGGCCTCAAAGCGATCGGCAATGCAGGGCAGCCAGCCAGCGTCAAGAATATCATGAAAC
TATTACCTGGATTCGGCACCGCAGCTGCCAGTATCCCACTGAAACTCCAGGTCGATGCTCTCATGGCTCTGAGGAATATTGCAAAGAAAGACCC
AGGAAAGGTACAAGCCATCACTATTCAGCTATTCATGAATCGAGGAAATCACCCTGAGCTCCGGATGTCTGCCTGTGCAATTTTCCTGCGCACC
AAGCCATCTTTGAACTCACTGTTAGTCCTCTCAAATTCCCTGTTAAAGGAGCCCAGTTTGCAGGTGGCAAGTTTTGCTTATTCGCAATTCAGAT
CTCTCGCAAGGAGTTCACTCCCTTCTCTCAGTTCCCTCGCAGCTGGCTGCAGTATGGCTGCAAAACTCCTAAGTCCCAGATTTGACCGACTTGG
CTTCCGATTCAGCCAAGTATTTCACCCTGACCTATTCTGCTACAAGCTGATGTCAGGAGTATCTGCTAAAGCCCTCGTAATGAACAATGCTGGG
AGCCTCATCCCAACATTAGCAGCGGGCAGAGTCCAAGGTCACGCTTTGGGATCTTCCGCCAATCTTGCAGAGGTTGGATTCCGAATGGAAGGTC
TCCAGGAGGTCATAACGAAAAGCCGTGCATCAGTCAGAGCGGCTCCTGATATGAAACAAATTCAACGGATTTTAAATGGGTTTCCAGACTGGAA
ATCCTTGCCAGAGAAAGTGCCACTAGCTTCAGCATACATGAAACTATTTGACCAGGAGATAGCTTTCGTGGAGTTCCGAAAGGATGATATTCAC
AAAGCCATTCAGTCAGTCATGGACGCACAAGGAAAACACAGTGTATTGAGAAACCTCGTAAACCGGCTCCAGAAACCGGTTGAACTGCATCCTG
CAGCAGCCTTACTGACAGCTGAGCTCCGACGCTTTGTACCCACATGTGTCGGTCTGCCCATGGAACTGACCTTCCTTTCTGCCGCTGTGGCAAA
AGCCAATCTCAACGTTGAAGCAAAGATTCCTTCCTCCATCTCTTCCTTTTCTCAATTGCTCAAGGCCAACATTCAGATGAAGGCTCAGATAAAC
CCAAGTGTGGCTTTATACAGCAAAGCCTTCATGGGAATAACGGCCAGCATCATTCAATCCGGTTTGGAATTCGAGGTAAAAATCCATTCGGCTT
TCCCTGTGGACATATCTGCAAATATAAATGTTCAAGAAAGGAATCTGAAGATTGAGAGCGCAGCACTTCAAGAGGAAAACCGAATCATATCGTT
CACGTCAGAGACATTCGCTGTTTCAAGAAATATAGAGAATCTATCTGCAGCAAAATTAACTCCAATTGTACCTGAGGCTAAAGAACCAAGCATT
ACAAAGCAAAAATTCAAGTCATCTGGACGTAGTCAATCAAATCCGGAGCTTTGTTCAGGAGTAATAACAGATGAAGCTGAATGTTATGATGAAG
AACAGCAACTTGCACCGAGTCCCTCTGTGATGAATGTTTGTATGGGGATGACGACATTTGGCTTTGACCTGTGTCTTGATGCAAAGACGGCCAG
TGCTGTTTACATTCGCCACGGGCCACTGCACAGATTGATGGGACTGCACACTGCCAAAGTTTCAATCAGGCCAGTCCAGTCAGAGACAAAAATT
GAAAGGTTATTGTTCGAAGTACAAACAGGCCCCAAAGCGGATTCCAAAATGATCCAACCACTGGGGAAAGAGGAACTATTACCTGAAAGAATTC
AGAGACGTACAGTCCTGTTCGAGGAGTTTCGCTCACAGACTAGAATGAAAAACCAGACTAGGACCAGCAGCTCCTCATCCAGTTCACTTTCCAA
AAGCTCCTCAAGAAGCAGGGCTACCTCTCGAAGATCTTCACTGTCGAGTGACCGAGTACTCAAAGGACACCACATCGCTGACCAGAAGCAAGAT
CAACCATTTCGGCTATCCAGAAATCGAAGCAACAGTGCAAGTCCTGATTCCAGTTCCAGCTCCAGCAGATACATGTTCTCCGACAACACATGGG
GAATAAACCAACAGCTCATGGATATTGAGTTCAAATCAGTCAGAAGCAGTGAGCAGACCAGAAGACGCCTATTGTCCAGTAACAAGCCAAGCTT

CAGAATTAGACACAGACAGAGTAGCTCGAGAAGCAGACAATCTTCCGAGCAGCGTGATCTTATCAGAGACATTGGTGCTCCGTCACTCATCATC

CTTGCTCGTGCTAGGAGGACTGATGGAAAACAGCAAGGATATCAGCTCACTGGATCTGTGCAAAGCTCAGGTGGCAGACCTAAAATGCACCTGC

GTATTGTCGATCTGAAGGAAGACAGCACGTGGAAAATGTGCGTGGATGCTGCCATCCCGAAAGCACACAAAGCAATGATGATGTGCAGATGGGG

TGAAAACTGTCAGACTTACAAGATGTCTTATAAAGCATCGATGGGATATCTTGCAAATCATCCAGCCTTGAAAATTAAGACTCAGTGGTCGGAA

ATCCCTCATCTGATGATAGCTGGCGGAAGGATGGTCGAGTCTGCAGCTGCTTACTTATTAGGATTTTCCAATAAATTTGAAGTTAATCCTTCTC

ACCAGATCACACAACTGATAGCCCTCACATCACCGCGGACCATTGATACAATTGTTAAACTACCCAGGTTTACAATGTATTACCAAGGCTTCGA

GCTCCCTATGCCAGTGCACATCCAAACAATGGCCCCAATCGTACGAACGCGAGGATTCAAAGGCATCACGGAAATCACTCGACTGCTTCTGACG

ACAAACCAACGGGAATGTATTGCTGAGAAAGAGCGAGTTGTTACATTCGATAGTAATGAACTAACATATAAAATACTTAATGATTGCCACTACA

TTCTGACCAAAGACTGTTCACCTGCTCCAAAGTTTGTTCTGTTGATGCGCCGTGCCAAGAGTCAGCAGGGAAAGAAAGCAATCAAGCTGCTGAT

ATCAGTGCCCCGTGTCGTAATTGAAGCATATCCCACACCAGATGGAGTAAATCTCTTGGTGAATGATGTTGAAACCAAGCTAAGTAATCAAGGA

AAGATTATTCAAATGTACAGTTCAGTCCACACACCATCCAAGTTACAACCAGCGGCCAGCAGAGGACTTAAAGACCTTGTAACCATTCAGCAGA

ATGCGACTGGAATTTTATTAGAAGCACCCTCAATCAATATTGACCAACTGTTCTTTGATGGAGACAGAGTGCAGATTGTACTCAATCAGATGAT

GAGTAAGACATGTGGTATTTGTGGACTAAATAATGGTGAGCAAAAAATGATGAAGCCCAATCAAGAGGAAGCCAGAGATGTTGAAGATCTTTTT

GAATCGTGGACATCTCCAGGGCAATCCTGTACAGATGACTGCAAAGTTGGACGAGAATTTGTGCAATTTGGAAAATTAGTCGAATTTGAAGGAC

TGGAATCCAAATGTTTCTCGGTTGAACCAGTTCAACGGTGCCTGGAAGGATGTTCACCCATTGAAACACGCTCTCAGATGGTCAACTTCCATTG

TGTGTCATCCGATTTCACTGTGAGGGACAATACAATGTTCAGTAGGAAGTCTCCAGACGTGCGACTCTCAGTAGATTCCCATAGCGACTGCATG

TGCAAGTGTGCATAA

>frilled shark VLDLRc1

ATGATTCCTTTTCGTGTGGTTTTACTGCTGCTAGCAGTGTGTTCATATCAGTGCAGACATATAAATGGCTCAAAAATATCTTGTGAGGCATCAC

AATTTCAGTGCAGAAATGGACGCTGCATCCCCTCAGTATGGAAATGTGATGGAGATGATGATTGTGCTGATGGAAGTGATGAAAGTACCTGTGC

TAAGAAGACCTGTGGTGGTTCTGACTTTGTGTGCCAAAATGGACAATGTTTGCCTAGTAGGTGGCAGTGTGATGGTGATGCTGACTGTGAAGAT

GGATCTGATGAAAGTCCAGAAGTCTGTCACATGAGAACCTGTCGTGCCAACGAAGTCAGCTGTAGCCCTGGATCTACGCAATGTATTCCTATGC

CCTGGAGATGTGATGGAGAAAGGGATTGTGATGATGGTGGTGACGAGGAGAATTGTGGTGCCCTTACTTGCAGTACACTGGAATTTACTTGCTC

TAGTGGCAGATGCATTTCCAAAACTTTTGTCTGCAATGGTGAAGATGACTGTGGTGATGGAAGTGATGAAAAAGGCTGTACTCCACCTACCTGT

GGCCCACATGAATTTCAGTGCAATAATTCAGAATGTATCCCACTGCAGTGGCTATGCGACTATGATATCGACTGCACTGACCAGTCTGATGAAT

CTCCAGAACACTGTGGCCGCACCCTTCCTCCCCTCATAAAATGCTCTGCTGGCGAGATCCAGTGTGATTCAGGTGAATGCATCCACCGCAGATG

GTACTGTGATGGAGATGCTGACTGCAAAGATGGAAGTGATGAAGTAAATTGCCCTCCTCGGACTTGTAGACCAGATAACGTCAGATGTGGTGAT

GGCAGCTGCATCCATGGAAGCAGGCAATGTAATGGGTTTAGAGACTGTCCTGATGGCACTGATGAGCTTAACTGTAGCAATGTTTCAGAGTGTA

CAGGGCCAACTAACTTCAAGTGTCATAGTGGAGAATGCATAGATATGACTCAAGTGTGTAATCAGCAGAAGGATTGCAGGGACTGGAGTGATGA

GCCTCTCAAAGAATGTGATTTGAATGAATGTTTAGTGAACAATGGAGGTTGCTCTCATATCTGCAGAGATCTTGTCATTGGTTATGAATGTGAT

TGCCCAGCTGGCTTCAAGTTGGTTGACAGAAAAACATGTGGAGATGTTGATGAATGCCAAAATCCTGGGATATGTAGTCAAATTTGTATCAACC

TAAAAGGAGGCTATAAATGTGAATGTTACAAAGGATATCAAATGGATCCAACTAATGGAGTTTGTAAGGCAGTAGGTAAAGAGCCCTATTTAAT

GTTCACTAATCGCCGTGACATCAGGCAAATAGGACTAGAGCACAAAGAGTATACTCAAGTAGTTGAGCAGCTAAGAAATGCTGTGGCACTGGAT

GCTGATATTGCAACCCAAAGAATCTTCTGGGCTGACCTGGGACAGCGGGCAATTTTCAGTACATTCGTGGATCAATTTTCAGCTGGCCATTCCA

GAATTGTTAATGATGTGCAAATCCCTGTGGGAATTGCTGTGGACTGGATTTACAAGAACATCTACTGGACTGATCTGGGTTTTAAAACTCTATC

TGTAGCTACATTTGATGGAACCAAAAAGTTGACCCTCTTTGACACTGGTCTAAGAGAACCAGCTTCTATAGCTGTTGATCCACTAACTGGGTTT

GTTTACTGGTCAGATTGGGGTGAACCAGCAAAGATAGAAAAAGCAGGAATGAATGGTGTTGATCGCCAACTCCTGGTTACCAGAGATATTCAAT

GGCCAAATGGAATTGCACTTGATCTTGTGAAAAGCCGCCTCTATTGGGTTGATTCAAAGATGCACACATTGTCCAGTGTGAACCTAAGTGGACA
AGATAGAAGAAGGGTGCTTTTGTCACCAGACTTCCTTGCTCATCCTTTTGCTGTTACTGTATTTGAAGACCATGTATTCTGGACTGATGGAGTG
AATGAAGCTATCTATGGAGCCAACAAGTTCACAGGAGCAGATGTGGTCCTTTTAGCCTCCAACCTTAACGAACCTCATGATATCATAGTTTATC
ACGAACTAGTACAGCCATCAGGCAAAAACTGGTGCAATGAGAAGCTGAAGAATGGAGACTGTGAATACATGTGTCTGCCTGCTCCTCAAATTAA
CAGCCATTCACCAAAATATACCTGCCTATGTCCATCTGGAATAGAGCTAGAGCAGGATGGTCAACTGTGTCAAATGGGAACTGGACATGCTCAC
ATTCGCCCATCTACATCTCGAGCACTTTCTGCTCCCCATGTAACTATGTCAACAGTCTTACCTGGCTCTAGAGATGTAAATGGCAGTGTTCCAC
TTCAAGTGAGCCAGTTTGGGAAAGGATCAGCAGCTGTTTGGATTATCCTTCCTATTTTGCTTTTGGCAATAGTCTGTCTGGCTGGTTACATTGC
ACGACGCAACTGGCAAAACAAGAACACAAAAAGTATGAATTTTGATAATCCAGTCTACTTGAAAACTACTGAGGATGACTTGAACATTGATCTC
AACAGACAAAGCCAATCAGTTGGTCATACCTACCCTGCAATCTCAGTTGTCAACACAGAAGATGATTCAGCTTGA

>spotless smooth-hound VLDLRc1

ATGATTCCTTTCCGTGCGGTTTTGCCGCTGCTAATTGCAGTGTGCTCCTATCAGTGCAGCTATGTAAATGGTTCAAAAACATCTTGTGAGTCAT
CACAGTTTCAGTGTAGAAATGGACGCTGTATTCCCTCTGTATGGAAATGTGATGGAGATGATGATTGCTCTGATGGAAGTGATGAAAGTACCTG
TGCTAAGAAGACTTGTGGTGGTTCTGACTTTGTGTGCCATGATGGACATTGTGTGCCTAGTAGGTGGCAGTGTGATGGTGATGCTGATTGTGAA
GATGGATCAGATGAAAGCCCAGAAGTCTGTCACATGAGAACTTGTCGGGTCAATGAAGTCAACTGTAGCCCTAGATCTACTCAATGTATTCCTA
TACCCTGGAGATGTGATGGTGAAACAGATTGTGATGATGGTGGAGATGAGGCAAATTGTGATGCTCTCACTTGCAGTGCACTAGAATTTACATG
CACCAGTGGCAGATGCATTTCAAAGACATTTGTCTGCAATGGTGAAGATGATTGTGGTGATGGAAGTGATGAAAAAGACTGTGCACCACCAACC
TGTGGCCCACATGAATTTCAGTGCAATAGTTCAGAATGTATCCCTCTGCAATGGGTGTGTGACAGTGATATTGACTGCACTGATCACTCTGATG
AATCTCCAGAACATTGTGGACGCACCCTTCCTCCCCTCATAAAATGCTCTGCTGCTGAGATCAGATGTGATTCAGGTGAATGCATCCACCGCAG
ATGGTTCTGTGATGGTGAACCTGACTGCAAAGATGAAAGTGATGAAGCAAATTGTCCTCCTCAAACTTGCAGACCAGATCATTTCCGATGTAGT
GATGGTGGCTGCATCCATGGAAGCAGACAATGTAATGGATTCAGAGATTGTACTGATGGCACTGATGAGCTTAACTGCAGAAACATTACAGAGT
GTACTGGTCCAACAAACTTCAAATGTCATAGTGGAGAATGTATTGACATGACTCAAGTGTGTAATCAGCAGCAGGATTGCACAGACTGGAGTGA
TGAACCCCTTAAAGAATGTAACTTGAATGAATGCTTGGAGAACAATGGAGAATGCTCCCACATCTGCAGAGATCTTGTCCTTGGTTATGAATGT
GACTGCCCTGCTGGTTTCCAGCTGATTGACAGGAAAACATGTGGAGATGTTGACGAATGTCAAAATCCTGGGGTTTGCAGTCAAAAATGTATCA
ACTTGAAAGGAGGCTATAAATGTGAATGCTATGATGGATATCAAATGGATCCCACAAATGGAGTTTGCAAGGCAATAGGTAAAGAACCCTATTT
AATGTTCACTAATCGTCGTGATATCCGGAAAATAGGACTGGAGCACAAGGAGTATACGGAAGTAGTTGAGCAGCTAAGGAATACTGTAGCCCTG
GATGCAGATATTGCAGCACAAAGAATTTTCTGGGCTGACTTGGGACAACGGGCAATTTTCAGCACTTCTGTAGATCCACGGAGTTCAGACAACC
ACTCCAGGATTGTTGGTGACCTACGAATCCCTGTGGGTATTGCTGTTGACTGGATTTACAAGAACATTTACTGGACTGATCTGGGGTTTAAAAC
TCTATCTGTGGCTACCTTTGATGGAACCAAAAAGAAGATCCTCTTTGACACTGATCTAAGGGAACCAGCTTCTGTAGCTGTTGATCCACTAACT
GGTTTTGTTTACTGGTCAGACTGGGGTGAACCAGCAAAGATAGAAAAAGCAGGAATGAATGGTGTTGACCGACAGCTCCTTGTTACCAGAAATA
TTCAATGGCCCAATGGCATTACACTTGATCTTGTGAAAAGCCGCCTCTATTGGGTTGACTCAAAAATGCACACATTATCCAGTGTGAACCTAAG
TGGACAAGATAGAAGAAGGGTGCTTTTGTCACATGAGTTTCTTGCTCATCCTTTTGCTGTTACAGTATTTGAAGACCGTGTATTCTGGACTGAT
GGAGAAAATGAAGCTATTTATGGAGCCAACAAATTCACAGGACTAGATGTAGTCCTTCTTGCTTCAAACCTTAATGAACCTCAAGATATCATAG
TTTACCATGAACTTGTACAGCCTTCAGGCAAAAACTGGTGCAATGAAAGAGTGAAGAATGGAGGCTGTGAACACATGTGTCTACCTGCTCCTCA
AATTAACAGCCATTCACCAAAGTACACCTGTGTATGCCTATCTGGAATGGAGCTAGCACAAGATGGCCAACAATGTATAATGGGAACTGCGAAC
ACTAGGCATACACCCATTCTCTCCCCCACAGCTCATCTCATAATGTCCACTATCCGGCCTAGAGATGTAAATACAAGTGTTCCACTTCAAGTGA
GCCAGTTTGGGAAAGGATCAACAGCTGTCTGGATCATCCTTCCTGTTTTGCTGCTGGTGATAGCCTGTGTGGCTGGTTATATTTCATGGCGCAA

CTGGCAAAACAAGAACACAAAAAGCATGAACTTTGATAATCCTGTCTACTTGAAAACTACAGAGGATGACTTGAATATTGATCTCAACAGACAA

AGTCAAGCAGTTGGTCATACCTACCCTGCAATCTCGATTGTCAACACAGAAGATGACTCAGCTTGA

Supplementary data 2.—Manually curated peptide sequences containing of the VTG genes analyzed in this study. See Supplementary table S1 for the other sequences used for the phylogenetic analysis in this study.

>tuatara VTG1

MKGLILALALTLVGEPDFSTNKVYTYNYESVILTGLQERGLSKAGIRIKSKVEISGIGSKLYLIRIHSLEASEYNGVWPREPFVRSSKLTQSLT

AQLNYPVKFEYSNSHVGNLMAPSALSEEALNIIRGILNMLELTMKKTQNLYNLQEAGIGGVCNTTYVIEENRKANQISVTKSKDLNSCDVKAKL

VTGAAYVTSCKACQKRNKNFRATATYNYKIKSTQEEALIVQADVLEVHQFTPTNELNGGHLLVEARQKLVLNDVAKRSPQVPSMEFQKRGSLRY

HFGNELLQLPIQLLKARNLESQIVENLQYLVEHTREQVPSDAPLKFLRLIQSFRRTNAETFDTIFKQYSSRPAYRRFILDAIPAVATHDAMRFL

RQKIERKELTDLEAIQALLMGLHFCTPSRDVIDEVMILRKINPHTDTYLHRIAYLGYSSLVQRHCSISDTCPDSALQPLREYLTEVLGKSNEED

IVLAIKALGNIGHTAIIKPLMKVMPGYTSVANYLSVRVQGIVVMALRNLGKKNPQQVQEITLEIFLNHKLHPTVRIIAAMVLLETKPALPILMT

LANAMLKEPGQVASFVYSHLRDLGRSTAPDLQVVASACRMAARILSPKLDRPGYRFSRVFRFNMFRESLMAGMSAKFFILNEASSIVPTFVMSK

IRAHFLGNIADVIEVGLGAQRLQEMLARSHVHDSDSDSSFDMSEILKQLSDFKSMPADKPIVSGYMKMFGQEVIFGGLGKNSMQSLLQALYGPE

EKIPLIKQLIKNLQTGLGTQWTKALMSAEVRRIVPTCTGFPMETSLYHASVTNIAGNVQAHITPAPSSSFRLADIISANVKLRSRMTFSMVKDM

IFVMGINTRLVQASLETHAKVTVNLPVNVAATINIKEKNLKLEIPPCNQETEIFTLRSKTYAVTRNIEEAPAAKMTPVLLPEAVPDMMKLSFDS

DLSSSGESDKIKERLSAEISPGNSYSAGQSSHHTAQVAHVYCFNGSTFGCKPCTEWKGISAEFIKNVPTYYLIGEHALKLTFKPVHTEASIEKI

QISIQEGAQAAAKMVRLVTFEDPKTELAASKQAVKKVKKILADPDEQRKSYTKQAPSSSSSSSSSDSSTSSSKSSSDSDRQITETGDKINLRK

AETSLEYSSKTDQKGHVKDQRSKENSKTKPAKKFQPFGQSSSSSSSSSSSSSSSSSSEEAKGRSHGKKHGTLSPADRINSDETKRGLHDPKHP

KPPRKHQSSSSSSSSSSSNSNSDSSTSSDSSSSRSSHSSSSSSSSSEPAKRGHHDEKKHAKGASKSDSKTQKVHSKSPFNSQSSEDSDWVTIYH

TSFLNITNFLGDLLPPGLTIVARAVRSDNKNQGYQATAYLNYKGNKVNMQLVMVQLADTNWKACADAALLSFFIKARLRWGKECRQYKIEASAT

TGQLAQNPAVQVKMEWTKLPSLIRKTTMQYVPGVALLFGFSEAQQHNPSNALVLRAVATSLHTIDTIIKAPGVTFYYQGFSTPVALPFGPSSPY

QSREITAWNFLSEMSFLIARQDECKCAICKVNEENFKTFDGQWYKTSFNKNCKLLVAQDCNTRPKFMIVTRKVNPRLPREVYINTASDNVKIYK

NETTIVVEARTQGLTNLTYDGTTLQVTVASWMRGKTCGICGNNDGEKRNDLLLPSRRQAHCSTQFVHSWVLPEKDCSEGCKLQRRFVKLRRQSK

VSGRETTCYSTEPILQCMDGFTATERTSIKLGFHCFPTDSVMSLSQWEKSNDRKTASEDVVEDVDAEIACTCPGRCT

>tuatara VTG2

MRGIILALVLTLVGKFICPVEPAFSNSKTYVVYNYEGLLLSGMQEKGLARSGLRLTCKLEISGMSQNAHLLKIRSPQLEEYNGIWPRDPFTRNSK

LTQTVTSCLTQFFRFDYNSGRVGNIYAPENTPLICVNIVRGILNMLQMTIKKSQNVYDLQEVGIGGVCHTRYVIQEDRKNSRVTIIKTKDLNNC

LDKVEKNIGMTLSLHAFLCFQKGKMVRGTASFTYKLKYADAGAVITQAESQQVYQVSPFNEPNGVAVMEARQELSLLDVKSEHVTPPESQLQNQ

GSLRFHFPAIMPQMPVQLIKTKNPEQRIAETLQHLVQNNQQDIHADAPYKFLELVQLCRVASADNLEAAWKQFADKPRYRRWLLSAVPASGTIE

AMKFVKLRFHNGDLNAFEEILVAVATFHLARINEHIIPELVSSSRIQRSPFLQQLALLAYGSFVDKVCSRSSSCPNEALQVLHDFATEAANKGH

EENMVLALKAMGNAGQTASIKRIAKFLSQSSDYSVHVQASAILALRKIGRIDPKKVQEILLQIYVSRSLPPKIRMMACVVIFETRPALPLITTM

ANVALKETNLQVASFVYSHMKALAKTTFPHLYNLSAACNIAIKLLSPKLDRLSYRYSKVIHMGVYYSVGEYRSGAIGRIFLMNSPKTMFPSAII

SSLGINYAGAYAQLAEVGLRAEGLTEIIRKQNIPFAEYSTYKKIKEIGKQLMGFKEVPSETTMLSTYFRFFGQELGYFQLNKGFIQQLVKVAFE

PADRHSAMKKIVNYFQNGIAGQLTQPLGIVEVRHIVPCVLGLPLETSLYATFLAHVSANVDMKISPSLDDNFRPSQLLEANVQLHANIHPRVGT

IGVNTQNFQSTVEIQGKARVRMPMKFDVKLDMKDKSFKFETTPLRQEIELLMIRTKVYAISRNVRELDSPKRTSLLPETAASDILKTQFKSEER

TFRDSICSKMPQFSFQICFSNSVFNARFLSATLLYRLIGVHEMRVMLKPVRTASPIDKIQLEIQAGSRAASKIIQLVRPEEDDDDDDDSSPDDE

IQAKLKKILGIEKMFKVPVLFFDLMLQVGNRTQHHKKQSVKKPQTTQLRKDTTARDSSSSSPSSSSSSSGAGKNNRGKSRDSSSSSSSSSDRSSS

SSSSSSSSDSSDSSSSSSDSSKSNSGSSSSDSSSSDSSSSSSSSSAPGRPETRHRSPSHAKDSSSSSSSSSSSDSSSDSRGSSSSSNSEYAKAKV

TLPCHFLGDSRPPLLAIIFRSLRSDKKMAGFQLVLYTDVFSPKPRIQVFVTNLTDSSKWKLCVDASFLSSHKAAAYLKWGRDCQDYKISAEAVS

GQFGDHPAMQLKLDWPKVPSAVRSIARLYTLVPGAAYFFGFTEKMRSNPSRQTRFIIALTSARTYDVVIKLPDVSTLYESAFRLPLSLPVGPRI

PASELQSPAWNFFSEAPSAVLENLKARCSVSQDKITTFNEVKFDYSMPANCYHILVQYCSPELKFLVMMKNAEESADLKAINVKLGKSGAYMLI

HNEKKGLSLKAAEYGIDKLYYNGHTIKVRVQVALWMAGKTCGICGKNDAETEQEFQTPSGYFAKDAVSFAQSWVLSEGPCTACKLQRSMVKLEK

AIQLEGEDSKCYSVEPVLRCVKGCAATKTTPVAVGFHCLPTGTTLRLVEGQMKLDQKSEDMEETVDANTACSCEELQC

>Tuatara VTG3

MREITLALIFTLVGKFFSSNTNRLLKGKEPGFSNSKTYIYSYEGFILTGLQEKGLARSGLRLTSKLEISRISQNDHILKVRSPRLEEYNGIWPR

DPFTRSSKLTQIVAPCFTQVFKFEYNKGHVGNIHAPEDTSVMCVNKVRGILNMLQMTMKKAQNVYDLQEVGDIGGVCHTRYIVQEDRKNNRITV

SKAKDFDNFQDRAVRIGMAYIRPCPTCKARNIRGTAAFTYRLKYADAGALISQAESQQVYQISPFNEPDGVAVTEARQELNLIDIRSSQVRTSE

VQLQNHGSLCFHFAEEQLQMPVHLIKTKNPGSQVVETLQRLIQQNQQGAQADDPAGFLELIELSRVMTQESLESVWKQFSDKHPHREWLLGAVS

AAGTIDTFMFLKQRLRNEDINDLESALILTFAIHLTKADDRTLDLLTSYRIQNSPTLHKLACLAYGSMVNRHCSLSLSCPIQVLQPLHNLATEA

ASKGQEENIVLALKAMGNAGEPASIKRILKFLPVFSPAAKAFPIRIHVEAVLALRKMARKDPAKVREILFQIFMDRSLSPEVRMVACVVFFEAK

PALPLVTAMTHSLLMESNLQVASFAYSHIKALSLSWRIPQLYNLSAACSIALKLLSPRLDRLSYRYSKAIQVGDYYRAGAIGRAYLMNSPNTLF

PSAFIMKLRGYYANSASDIVEVGLRAEGLTEIIRKQNIPFAEYSTYKKIKEIGKLLGWKELPSEKPLVSAFLKVYGQDIVFGDITKELIQQATK

VWLEQAASHTLVKKMINAIQQGVAGKWTQPIFAGELRHIIPSCVGIPLEFSLYATGLIQVAANVVKTSPSLSGDFRPSQLFDVNMQVGAEINPH

TVATMGINTPHFQSGLEFHAEFHANTPLKFDASISMKDKNMKLETTPLQQETELLAVRHKAFAVSRNIAEIDSEKKTPVLPDGTVSDILQEHFK

SSEKTSQEGTGRRSYAQVFCGILPSLGCQTCLNMKLQGAASIKNTYLHKVVGKHEINLVLKPINKIQLEIQAGVRAASKIIQLVSSEEEEDGDS

SPDDEVQTKLKKILGIENVFKVQLDLQVGNRSSQQHFNKQSAKKDKTTPAHIHRDPKARYPSSSSSSSSSPSSSSSSSSSSSSGNKNRNKDTE

VKKKKTAQREQSNSDSSSSSSSSSSSSSSSDSWDSSSSSSGRRGSSSSDSESARKQVIQSDSHHCNDYFLGDSTPPVLAVILRTIRNDRRLAGFQ

FVLYSDLHSLRPRLQVFVSNITAASKWKICADASILHSRKAGSVKWGHNCQDYKIAAKIATGQFAGHPAVQVKLQWPRVPLRVRMPARWLYTFV

PGIAYMLRFFQREQNNPSHQATLMVALTSPRSSDVVLKLPDVTIYDSGIRLPVPLPLGPRISVSAQHSHAWSLFSEVPSAVFENLKGQHFSSQN

TITTFNEVEFNYSMPANCYHVLAQDCSPELKFLVMMKNAEESADLKAINVKLGRQEIDMYPSNGLVKLMVNGVETPAESLTYFILLDTSLMIRS

ESEGLSLAASDYGIDKLYFDGRTFKVQVAFWMAGKTCGICGKYDAEIEQEYQMPSGYLAKDAVSFAQSWILSEGPCCKLQRTLVTSEKTAGFEK

VASKCYSVEPVLRCVEGCSATSAVPVSVGFHCVPSDSTLHEAEIRLDQKPEDLEDVVNAHTACSCEQLQC

>small-eyed rabbitfish VTG1

MRGIILALCLVIVGKEPNFISRRTYTYKYEGVILTGLPEKDLARGGLKIICKVQISSIRQNIHLLKIISPQIQEYSGIWPRAPFLPAWKLTRRL

TADLNKPVKFEYNHGRVGNIYAPADLPENILNIYRGILNLLQITIKKTQNIYNLQEMGVEGICHANYLLQEDKKAGQITITKSKDLNNCQEKAS

KTMGIPFTELCESCQLKGKNLRSFSTYTYTMKTKQGGTQIVEVNSHETHQFTPFNELEGGVLLEARQHLIFLDSTSQSLPTTSYHMENRGTLIY

QFSNELLQMPMHLFKTSNNDTQIAEVLENLVQKNQEKAHSDSPSKFLQLIQLLRSATYQSIQTIWKKKAARSEYRRWILDTLPSVGTIEALTFV

KTKIEEGALTQFEAAHVLLFTLHSITPDCHGVDNATVLLSSPYIQRTAFLRRITLLAYGSLVKKYCGTVQVCSEETLQPLHKLLVEASSKSNEE

DIILSLKAIGNAGHPASIKRIQKFLPGFGTGAATFPARIQREAIMALRNIANLQPRKVEEITIQLFMNSKNHPEVRMMAFVVFFETKPSMASVA

IITDSLIKETNLHVASFTYSFMKALAESSLQDLHSVATACNIAIKRLSPKCDQLSYRHSKALHFDTFKDKLLAGLEADFYLINNGASILPTSAI

AKFKVYGLGGAANLLEVGIQAEGLKEVIQKSSPPDRKQSDNSQRIMKRLSGWKPLPTIKPLAVAYFKLFGQEVAFVELNENDIQEALKLFNNPV

KKNALLMKLIKQLQQGLTTQWSKPLLATEARHIVATGIGLPMDLSLHYSILTAISVNAKAQFTPNPSSNVTAAKLLNSTIQLNVQVTSAVKDII

AIMGINTQLIQTGVQMKVKTRTIIPVDFTAEVNLKDKNINIETSPLQQENYLVSARSQTFAFSRNIEDLAAAKITPILPTAAEIAMINRNFGIA

RNTSQDSIVMQARESPFMAQQGTVCSAEEALNKRKPTEHKVCVQGSTFGFEVCYQIKAENTAFIRDSPLHKTIGENAFQVSIRPVATTPPIKKI

QIEIQAGEKAGAKVIRSIRKDNMRQSDDSEDTELRGKMSLLNKLRNSKEQIQFLGDSMPPIFVILAQAIMSDNKKMGYQTTAYIDRMAAHPLMQ

LFVDELEGEGKWRACVEAEMPNEHRAGVLKWGKDCKNYMIVAKASTSQYEHHPAMQFKVQWDRIPHYLKHSSEMVAEYLPGVAFMLGFSEKHQQ

NPSYQLSMTAAATSPRTIDLFIETPKVILSRRAIAIPVALPVDVKSPSLQPSGFHILRELPLMLFSKDECTVMENNVTPFTENSFEYEMPGDCL

HILVQDCTSELKFIIMIKRATESPNSLSLILRLPSGVIGIDSTAAGDLRLFINGNEMSITSLPLPESITFDVDNDKVNIEAAELGLEKLYFDGK

RIKVASWMTGRTCGLCGRGDSKTRNKYNLPNGQSTKDVVKFAQSWLLQGERCSDACKLQKKIVKREKQIKLPGQESNCFSIEPVLHCRPGCSPI

KTVPVSIGFHCLPEDSFITDLADHRLDNLDQKSEDLQEVIEAHTACSCASDCTRADI

>small-eyed rabbitfish VTG2-1

MRGIIFLLILTIVGKPNFSEKHTYVYMYEGVVLTGLPEKGLNRAGVRINAKVKISALSQRNYLMQIIDPQIQELNGVWPNVPSSTASKLTQKLA

ADLTKPITFEYSNGRVGNINAPEDLPENILNIQRGILNMLQITVKNTQNVYELQEGIQGICNTKYVLQEDRKTNQITVTKSKDLSNCQERVQNQ

LGLAYALPCPACQKNFRSSVVLTHILKPKVSGAIIVEARTREIHQFTPFHELDGTAMMEAKQNLILDDIKVELVREPQERLQNRGSLKYKCTTN

LLQKPIQLLKYQSLDAQIIDTVRHMGQYNDHEVHFDAPERFLELIQLLRTASFEENVLTLFPKSRRWFLDALPAAGTADTLRLLKRKIQSSEIK

TIEAAQALIFAMQQLKADRQSMPIAKVNQIRQSPILRKIVLLGYGSVVYRFCAERRSCPEDVLQPLHDLLADAGSRSHEQEIVLGLKAVGNAGQ

STSIKRIQKFLPGFGSAASSLPLKVQVDAVASLRNIARRDPRKVQDITIEIFMNRRNHPEVRMVACVVLFSTKPSLTLVTAVADSLLKETSLQV

ASFTYSHMKALSRSSVHVNRAASCNVAVKLLSPRLDRLGIRYSKVFHIDMFRYNLMTGASARLLLLNNAGSTIPSAILGKIRGHGLGGSMDLVE

VGVRAEGLQEALMINRVPLSKRMNTEQMLRILKQLSGWKGLPEEVPLMSAYVRLFGQELAFAEIRRDDIQQATQVQTLSTSAQNQLKSSMEQLA

RGIKVRPAKALLVAEIRHVMPTGLGLPMEVSLISSAVAVAKVNPITRISQLLSSTIHISTTIIPSLAIVTKAAMGINAPFMQAGMELQAKVNIK

QPINVIARFNVKEQNLKIETKPYAEERRVISLKSQVFVTSINIEDLSSAKRTPILPSANEPSLTAQDFQLSPEIISQETSFSEEQRRIPRASTY

HTCALTTKLGLKVCLDARMKSSVFIKNCLLYRLIGQHAVNVTVHSESEIERIVLEIQTGSKAVSKRIRLTDQEDQQSGRIQEERSSTRMSMRSF

PGGNPSSGTQRRQSSSSSSILRSSRQVCFLLNSKVPGCLRTETICSPINHFKMYLFVTLGRIPMRFSSSSSSSRQTQSSRTQDRRQMSSSTSSS

SSSRRTWSDQVTILAGPSHWQAIKLITPTKYNNNNLHLCSTTLYTHTHTHTINSLYTIFCEALRDVSASCSATVKHTWTLLCVRDTIEMQVVAV

VLTTRSAGSPILTILLRAVSTDRKQQGYQITGFAEVSDRQPRFHLRVVELAEDSRWKICADGIMPHGNKAMLLRWGQNCQDYKVSAKASIGQLA

RNPALKIKVQWSRIPDWLKSSGRMVGGSLPGIAYLSGMSQRYQSNPSRQITVLFALTTPRTIDTIIKVYKMTAYYLGAQIPLAVPVSAIAPRIK

ARGFKSITEIPDIFLTVNQRNYYFYSCLAEPGKVQTFDNQTLKYQIPNDCYYVLTQDCSSVPRFVLLMKRAKDQTRKWIKLILSNPNRVIEAYP

EGQDIKLKMNNVELPISSLPRVEQGEIQTNGTGILLVASELNLDSVYFDGNKVQVSVNQMNKRTCGICGYNDGERKMIMPNHNQTQSVDVFFQS

WLCSGTTCQDCKVRQEFVEVDSSLSFEGRESRCSPIELVQRCLPGCAPTERVPVSVAFHCVPSSKFFADAFLHTVLYSFWRMLSSFDVRRSVDS

HSDCIE

>small-eyed rabbitfish VTG2-2

MRGIIFVLALVLAGKISAPRFDERKTYVYQYEGIILTGLPENGLNQAGLKITCGVQISRLTQRIHLLNITVPQIQELNGVWPNVPLSAANKLTE

SLAADLTKPVKFEYSNGRVGNINAAEGLPENILNIHKGILNMLQITIKNTQNVYELQETGIEGICHTKYVLQENRKANHIFVTKSKDLDNCQER

ALKQIGMAYTMPCPTCKNLRSMATFTYTLKPKESGSLIVQIESREIHQFTPFHELDGTAVMEAKQHFTLNDIIAAKVSEPQVDFQSYGTLKHHW

DSDILQKPIQLIKYQTLETQVIETLNHLALNNQEQLHSDAPNQFLQLVQLLRTANIEQENVFTLFLKCRHWILDALPAVGTADALRFLKHKIQY

SEMKATEVAQALILAMQQLKADRQSVSVAKVAHVQHSSILRQIVILGYGSVVMRYCAVHQTCPSDALQPLHNLLIEADRHSHEHDIVLSLRAIG

NAGQPSSIKYIKKYLLDFGSAATHLPLNIQVDAVSTLRNIAMKDPRKVQDITVKIFMNRKNHPEVRIMACAVLFSTKPSLTLVTAVADSLLKET

SLQVASFTYSYMRVLSRLTIHVNRAASCNLVIKLLSSRLDKLGSRYSRVFRMDMFRNLMTGASAEMLLINNAGNAIPSAILGKIHGYALGSSVN

LVEVGLRAEGLQGALLRNHVTLSGKPNIEKIQRILKMLAGWKALPEEVPLVSAYIRLLGQEIAFVEFKKDDIREATQVDTRGSKALTGLRKFVD

RLRNQIGLHVSVALLAAEIRHVIATSVGLPMELSFHSSVVTVATANVQARISPSYISSIFQLLNANIQLKTTINPSVVIYTKAFMGIIAPFIQA

GVELQAKVCMDHPVNVVVRINLKERNLKIESKPAQKENRIISFKSQVYAVSRNIENLAATKMTPILPVNTNTSQAFVAQWSSEIISKETEQRKR

SQVARTSAYKICSRISELGIEFCLDASIEDAACIRLSPLYTMIGEHAFTILVRPESEVETIVLEIQTGSKAASKVIRLMQWENQQTVRIQRKYR

VLSSLCDIPKRITEYIVKLIDCIFCGQNFLKMLFDLQSGRRNTSIGSSPSSSSRRSYHFTRSERRSSRRSLDTMRGASPDDQGLERRSNRLQSS

SSSSSRLISSSKNRSSDRRRMSDRRRSSDRRRSSDRRRSSDRRRSSDRRRSSDRRKSSDRRRMSDRRESSDRRGSSDRRRSSDRRRMSDRRESS

DRRGSSDRRGSSDRRGSSDRRGSSDRRESSDRRGSSDRRGSSDPILTILLRAVRIDRKQQGYQITGFTEVSEHQPRVHLRLVELTEDSRWKICA

DGIMPHGHKAMVLLRWGQNCQDYEVSVKASTGEFSSHPAIKVKLQWSRIPDWLKSIAYLSGMSQRYQSNPSRQITMLVILKTPQSIDSVIKVPK

MTVYYQGAQFPLAIPISAVSPWIKARGFRSVMEIPDLLLTVNQRCTAEQNKVLSFDNDQLEYQISNNCYYVLTQDCSSAPKFVLLMKRAKIQSK

KSIKLLMSNPNLVIEAYPENQDIKLLIDNVECPVSSLPRTEQGIVQIRTNGTGIILEAKHIQLDLLYFDGVKVQVVVNQMNKQTCGICGHNDGE

RKMIMPNHNQTQSVDIFFQSWLCSGTTCQDECKVQSTFVELESPLRFGGRESRCSPIEPVQRCLPGCQPTEKVPVSVAYHCEPTGKFVAYFAFS

ADTYMGQLLSFDLHHSVDSHSDCAEA

>small-eyed rabbitfish VTG2-3

MRGIIFLLGLTLVGKSQPNFSERQTYVYKYDGVVLTGLPENGLNRAGVKISARVQISALAQTNYLMMIKDPQIQELNGIAPSVPLPTTSKLSQK

LAADLTRPVRFEYINGRVGRIDAPETLPDNILNIHRGILNLLQITIKKTQNVYELQEVGIEGICLTRYILQQDRKINQIIVTKSKDLSNCQDRA

IQQIGVAYALPCPTCQQRGKNLQGTVAITQLLKPTKSSGALIIEARSQELHQFTPFHERDGSAILEAKQNLILDDIRAEAVPVPQVKLMPRGSL

KYTTDHSVLQKPIQLLKYDNLEAQIVNILRYLAQYNQQEVRMDAPDKFLQLIQLLRLASFETIALTLFPRCRQWLLEALPAAGTADTLRLLKRK

IQSSEIRTIEAAQALVLAMQQLKADRQSMPIELLAIDQIRQSPILRKIVLLGYGSVVYRFCAERQSCPEDVLQPLHDLLADAGSRSHEQEIILG

LKAVGNAGQSTSIKRIQKFLPGFGSAASSLPLKVQVDAVASLRNIARRDPRKAQEITMQIFMNRRNHPEVRMMACAVLFSTQPSLVLVAVVADS

LLQETSLQVASFTYSHMKALSRSSLPSLNSVAAACNVAVKFLSPKLDRLGVRYSKVFRTDMFRNLMTGAAAKVLLMNTAGSAIPTSILAKVRGH

ALGSSVDLAEVGLRIEGLQEALLRNRVTFSGRLNIEKIQRILKMLSGWKSLPEEVPLASSYFKLFGQEIAFVELRRDDIQQVTQVPDLQNQLKR

YAEQLQRGIKARPSKALLLAEVRRVVPTGLGLPMEISLITSAVGVAKINVQAQTTPSSLSRISELVSANIQLDVTPSVVTFTKAVMGINTRFIQ

AGVELQTRVRISQPINVAAKINIKERNLKIESKPSVEERRMSSQVFAVSRNIEELSAAKMTPILPITSEASITKQEFPSTSTYYTCVKTTKFGL

AVCLDARMKSTAFIRNCLLYRLIGQHAVNVTVKPVESEIEKIVLEIQTGSKAASKVIRLDKMDDNLQRPERIPMRFSSSSSSSRQTQSSRTQDR

RQMSSSTSSSSSSSRRTWSDQVTILAGPSHWQAIKLITPTKYNNNNLHLCSTTLYTHTHTHTINSLYTIFCEALRDVSASCSATVKHTWTLLCVR

DTIEMQVVAVVLTTRSAGSPILTILLRAVRIDRKQQGYQITGYAEVSDDQPRVHLRVVELAEDSRWKICADGIMPHGHKAMVLLRWGENCQSYK

MAVKASTGRLASHPAIKVKLQWSRIPEMLTTSGIMVGESLPGIAYALGWSQSYQSNPSRQITLLVALTSPRTIDTIVKVPKMTVSYQGAQIPIA

VTISAIPSTIRARGFKFITEIPDILMKVNQRNYLFHFHLHYSSIPELLDLSRSTLKYQIPNDCHYVLAQDCSQSPSFVLLMKRAKDQTRKSIKL

VLSKPNRVIEAQPEGQGIKLLIDGVQCPMSSLPRVEQGEIQTNGTGILLQATEINLDLLYFDGNKVQVVVNQMYKRTCGICGHNDGERKMIMPN

LEKTESVDVFFQSWLCAGESCKDCKVRREYVELEELVSYEGVESRCYSVEPVQRCFSGCSPTEKVSVPVSFHCVSSGKVLYHMQGLLLSLLLFS

LNLPLLKHIFHISTASSMDRFQEMSFDKKSADLRRYVDSHSDCA

> small-eyed rabbitfish VLDLRc1

MLRTCGVNEISCGSASLQCIPSPWRCDGEMDCDNGGDEQNCGQLTCDVLEFTCSSGRCISRTFVCNGEDDCGDESDEQECAPPTCGPHEFHCNS

SECIPLRWVCDSDIDCMDQSDESSDHCGHTIPPLVKCSTSEVQCGSGECIHRRWYCDGDADCKDESDETNCPPRTCRPDYFRCGDGSCIHGSRQ

CNQFRDCIDGSDEISCQNITECTGPTNFKCQTGECVDMTLVCNKQRDCRDWSDEPLKECGLNECLENNGGCSHVCRDLVIGYECHCPPGFKLVD

SKTCGDIDECQNPGICSQICLNLKGGYKCECRAGYQMDPANGVCKAIGKEPYLIFTNRRDIRKLGLEHKEYTQVVEQLRNAVALDADIVAQRIF

WADVGQRAIFSVSMDRNGRAIETSRVAEVQMPVGIAVDWIYKHIYWTDMETKTITVATFDGTKMKILFDSDLREPASVALDPLSGFIYWSDWGE
PAKIEKAGMNGVDRQLLVSTNIQWPNGIALDLVKNRLYWVDSKLHTLSSVDLNGGDRRRVLLSQRFLAHPFAVAVFEDQAFWTDGENEAIYGAN
KFTGEDVKLLASNLNEPQDIIIYHELVQAPGKNWCNSSLPNGGCDYLCLPAPQINSHSPKYTCVCPSGDELQKDGHHCRTDSNSSAKVSLVNQS
GKAPTAAWVILPILLLAIAGVAGYLSWRNWQSKNLKSMNFDNPVYLKTTEDDLNIDLNRPGQSVGHTYPAISIVKTEDDMA

>small-eyed rabbitfish VLDLRc3

MDHVFWIDGQNEALYGASKLTGENVVILATNLKEPRDIIVYHELIQPPGTNWCNEGVKNGGCEYLCLPAPQINSLSLKYSCVCPPGMQLKADGQ
MCTNDSSRCHVNEFSCGPRSIRCIPVSWKCDGGKDCDDGSDEDDCAQPTCNPLEFTCSSGQCVSKTYVCNGEDDCDDGSDEQGCRTPTCEPHEF
QCNNLECIPLSWVCDAKHDCSDESDESASFCVRTLSPVTCSPHDFQCSSGECTHEYWRCDGDTDCKDGSDEVNCPTQTCRPDYFRCDDGHCIRG
GRKCDEFKDCNDGSDEVNCKNVSECKEPNNFRCQSGECIDITKVCNHIQDCRDWSDEPFNGCKVNECLVNNGGCSDICKDLVIDYECDCPPGFE
LVDGKNCRDIDECQNPGTCSQICINSKGSYKCECRAGYRMDPAYGLCKAVGKEPYLIFTNRHDIRKLGLHHKEYTQVAVQLRNAVALDADIAAH
RIFWADLGEQAIFSMSMNKWEGTAGILRPVKVAQMPVGIAVDWIYKHIYWTDRGTKTISVATFDGTKSKMLFDTDLREPASVAVDPITGFIYWS
DLGEQAAIEKAGMNGGHRQVLVNTNIQWPNGIALDLVKSRLYWVDSKLHMVSSVDLNGQVRIDFFSQFLVHPYAVALYEDRIFWSDGENNTIYE
ANKFTGADVFVLASSLKEPRGIIVYHELMQPSGRNWCSESLQNGGCEFLCLPAPQINIHSPKYTCVCPSGRNPDKGEPCGTAKATCGASDFRCN
NGQCVLGKWRCDGKPDCKDGSDENPELCHTSSCNINEIACGPGSLHCIPVSWKCDGGKDCDDGSDEENCAPFTCSPLEFTCSSGQCISKTFVCN
GEDDCFDGSDEHNCAPVVCGPHEFQCSNSECIPFNWVCDDNADCIDESDESPAFCGHTLPPHVTCSLSEVQCASGECLHQQWYCDGDTDCEDGS
DEVDCPPKTCRPDHFRCGDGSCISQNKKCNRFQDCVDGSDEVSCENALECTGPTMFECGSGECIDFNLVCNRRQDCRDWSDEHLKTCNVNECLV
SNGGCSHICIDLVIGYECDCPIGFQLVDERNCDDIDECLNPGTCSQICINSKGSYKCECQAGFHMDSANETCKAVGKEPTLIFTNRHDIRKLGL
HHKEYTQVAIHMRNVVALDADVEAQMIFWADVGEHAIFRLSMAKWEGVSKIVDAQMPVGIAVDWIYKHIYWTDRDAKTISVATFDGTKMTILFD
TDLTEPASVAVDPLSGFVYWSDCGEPAKIEKSGMNGVDRQVLVTKEIQCPNGIALDLVKSRLYWVDSKLHTLSSVDLNGHVRRTVIQSKDSLVH
PNAVSVFEDQVFWIDGQNGTIYGANKYTGEDLVVLASNLKEPQDLIVYHELIQPIGINWCNKSLKNGDCEYLCLPAPQIAGHSLKYACVCPSGM
HLDDGQRCRLDIRACPFNTLSCDPGFAHCIPVAWKCDGEKDCENGSDEENCAQSTCSPLEFMCSSGRCVSKTFVCNGEDDCADGSDEQGCSTPI
CGPHDFQCKNSECIPLTWVCDDNTDCTDQSDESLENCGHTLPPIMCSFSETQCGSGECVNRSWYCDGDADCKDGSDEVNCPPRTCRPNQFTCTD
GTCIQEDWKCNEMRDCVDGSDEVNCANAAECLGPSDFKCQNGECIDVAQVCNQQLDCHDWSDEPSYCNLNECLVDNGGCSHVCRDLPIGSECDC
PSGFKLIDGKTCGDIDECLSPGTCSQICINLKGSYKCDCHTGYLVDPATGLCKAAGKEPFLIFTNHHDIRKLGLHHNEYTQLAVQLRNAVALDA
DIAAQRIFWADLTERAIFSMSMDKQSTSGILKVISGVQIHVGLAVDWIYKHIYWTSLNIRAISMATFDGTKVKTLFDTDLREPASIAVDPISGF
IYWSDCGEPATIEKSGMNGGGGRQLLVTKGIQRPNDITLDLVKSRLYWVDSKMHVLSSVDLNGQDRRTIVQSKEFLTHPFGVAIFEDHVFWTDW
EKKSVYGANKFTGEDIVNLASNLEEPQDIIIYHELTQPPGENWCNMHFTNGGCEYLCLPAPRIHGYSPKYTCVCPPGMQLEHDEKHCRTVKSTC
TISDFMCHNGQCVPERWQCDGNTDCTDGSDESPEVCHMKKCPINEISCGPGSLQCIPVAWKCNGEIDCETGSDERDCGHLTCSPVEFTCSSGRC
VSKTFVCNGEDDCGDGSDEQGCAASPCGLHEFQCNNSECIPVTWVCDHNADCTDQSDESPGHCGYTLPPPMLCSSSEMQCDSGECIHRQWYCDG
DADCKDGSDEIDCPPRICKPDQFRCNDGSCIIGSSQCNGIGDCTDGSDEVNCVKAQVCRPDQFRCGDGICIVGSMQCNGFGDCADGSDEVNCEL
TVAKCTGPANFKCLSGECINMTQVCNQHQDCKDWSDELLKKCYINECLDNNGGCSDICWDLVIGYECECPSGFELVETKMCEDVDECQTPEICS
QICTNIKGSYICECHAGYHMDQTDGVCKTLGKEPFLIFTNRHDIRKLGLHHKVYTQVAVQLRNAVALDADIAAQRIFWADLGEQAIFSVSTDEQ
EGEVGISRVVDVETPVGIAVDWVYKHIYWTDRGTKTISVATFDGTKKMILIDTGLREPASVTVDPATGFIYWSDWGEPAAIEKAGLNGGDRQLL
VNKKIQWPNGIALDYVKNRLYWVDSKLHTLSSVDLDGQNRRTVLHSPQFLAHPSAVSLFEDRAFWIDEESKAIYASNKFTGEDVVILAFNLNEP
RDIIVFHEQVQLSGKNGCTNFKNGSCEYLCLPAPLFNGLSVKYTCVCPSGMELYNGLQCRTASLQYSAPLISTTGQTTAIPTSGGTSTELASNL
PTVLPNSRDVNPGASVHKSRNSTVTGWVVLIMMLVAVVGVAGYLKWRSWKSRTHQSMNFENPVFHRDDVVEEK

References

Blum M et al. 2021. The InterPro protein families and domains database: 20 years on. Nucleic Acids Research. 49:D344–D354. doi: 10.1093/nar/gkaa977.
Brodie R, Roper RL, Upton C. 2004. JDotter: a Java interface to multiple dotplots generated by dotter. Bioinformatics. 20:279–281. doi: 10.1093/bioinformatics/btg406.
Chana-Munoz A et al. 2017. Multi-tissue RNA-seq and transcriptome characterisation of the spiny dogfish shark (Squalus acanthias) provides a molecular tool for biological research and reveals new genes involved in osmoregulation. PLoS ONE. 12:e0182756. doi: 10.1371/journal.pone.0182756.
Fonseca E et al. 2020. Cartilaginous fishes offer unique insights into the evolution of the nuclear receptor gene repertoire in gnathostomes. General and Comparative Endocrinology. 295:113527. doi: 10.1016/j.ygcen.2020.113527.
Hara Y et al. 2018. Shark genomes provide insights into elasmobranch evolution and the origin of vertebrates. Nat Ecol Evol. 2:1761–1771. doi: 10.1038/s41559-018-0673-5.
Katoh K, Standley DM. 2013. MAFFT Multiple Sequence Alignment Software Version 7: Improvements in Performance and Usability. Molecular Biology and Evolution. 30:772–780. doi: 10.1093/molbev/mst010.
Nishimura O et al. 2022. Squalomix: shark and ray genome analysis consortium and its data sharing platform. F1000Res. 11:1077. doi: 10.12688/f1000research.123591.1.
Simakov O et al. 2015. Hemichordate genomes and deuterostome origins. Nature. 527:459–465. doi: 10.1038/nature16150.
Yamaguchi K et al. 2020. Inference of a genome-wide protein-coding gene set of the inshore hagfish *Eptatretus burgeri*. Genomics doi: 10.1101/2020.07.24.218818.
